# Yield of clinically reportable genetic variants in unselected cerebral palsy by whole genome sequencing

**DOI:** 10.1038/s41525-021-00238-0

**Published:** 2021-09-16

**Authors:** C. L. van Eyk, D. L. Webber, A. E. Minoche, L. A. Pérez-Jurado, M. A. Corbett, A. E. Gardner, J. G. Berry, K. Harper, A. H. MacLennan, J. Gecz

**Affiliations:** 1 Robinson Research Institute, Faculty of Health and Medical Sciences, University of Adelaide, Adelaide, SA Australia; 2 Adelaide Medical School, Faculty of Health and Medical Sciences, University of Adelaide, Adelaide, SA Australia; 3 Kinghorn Centre for Clinical Genomics, Garvin Institute of Medical Research, Sydney, NSW Australia; 4 South Australian Health and Medical Research Institute, Adelaide, SA Australia; 5 Hospital del Mar Research Institute (IMIM), Universitat Pompeu Fabra and CIBERER, Barcelona, Spain

**Keywords:** Neurodevelopmental disorders, Genetic testing, Neonatal brain damage, Molecular medicine, Medical genomics

## Abstract

Cerebral palsy (CP) is the most common cause of childhood physical disability, with incidence between 1/500 and 1/700 births in the developed world. Despite increasing evidence for a major contribution of genetics to CP aetiology, genetic testing is currently not performed systematically. We assessed the diagnostic rate of genome sequencing (GS) in a clinically unselected cohort of 150 singleton CP patients, with CP confirmed at >4 years of age. Clinical grade GS was performed on the proband and variants were filtered, and classified according to American College of Medical Genetics and Genomics–Association for Molecular Pathology (ACMG-AMP) guidelines. Variants classified as pathogenic or likely pathogenic (P/LP) were further assessed for their contribution to CP. In total, 24.7% of individuals carried a P/LP variant(s) causing or increasing risk of CP, with 4.7% resolved by copy number variant analysis and 20% carrying single nucleotide or indel variants. A further 34.7% carried one or more rare, high impact variants of uncertain significance (VUS) in variation intolerant genes. Variants were identified in a heterogeneous group of genes, including genes associated with hereditary spastic paraplegia, clotting and thrombophilic disorders, small vessel disease, and other neurodevelopmental disorders. Approximately 1/2 of individuals were classified as likely to benefit from changed clinical management as a result of genetic findings. In addition, no significant association between genetic findings and clinical factors was detectable in this cohort, suggesting that systematic sequencing of CP will be required to avoid missed diagnoses.

## Introduction

Cerebral palsy (CP) is the most common physical disability in childhood, with estimated incidence of ~1/500 live births worldwide^1^. It is a diagnosis based on clinical signs rather than aetiology, and is characterised as a permanent and non-progressive disorder of movement or posture, resulting from damage or abnormality of the developing brain^2^. Clinically, CP encompasses a highly heterogeneous group of disorders frequently co-morbid with intellectual disability, epilepsy, autism and visual and hearing impairment. A number of pre and perinatal risk factors have been described for CP, including multiple birth, prematurity, placental pathology, intrauterine infection and intrauterine growth restriction^3^. Less than 10% of CP can be objectively demonstrated to be due to acute perinatal asphyxia^4,5^, with many cases of CP likely the result of long-standing intrauterine pathology rather than a single event during late labour or birth.

Recent exome sequencing and copy number variant array studies have demonstrated that between 10 and 31% of CP cases have a genetic cause^6–14^, with a heterogeneous underlying genetic landscape. Nevertheless, the cohorts included in genetic studies have been diverse and reporting of clinical characteristics of cohorts has been variable^15^, leading to some scepticism amongst the medical community about the validity of these findings^16^. Prior genomic analyses (exome sequencing, DNA microarray, or gene panel^7,11,14^) of more than 300 cases from the Australian CP Biobank cohort suggest that genetic aetiology is not limited to individuals without other risk factors, or with other neurodevelopmental comorbidities like intellectual disability, epilepsy or autism. Therefore genomic investigations of select CP cohorts likely miss a component of CP aetiology. We performed whole genome sequencing (GS) on a cohort of 150 singleton CP cases and assessed the burden of pathogenic/likely pathogenic (P/LP) single nucleotide variants and indels, as well as copy number variants, with the aim of comprehensively assessing the clinically reportable genetic burden of CP in an aetiologically unselected cohort.

## Results

**Fig. 1:**
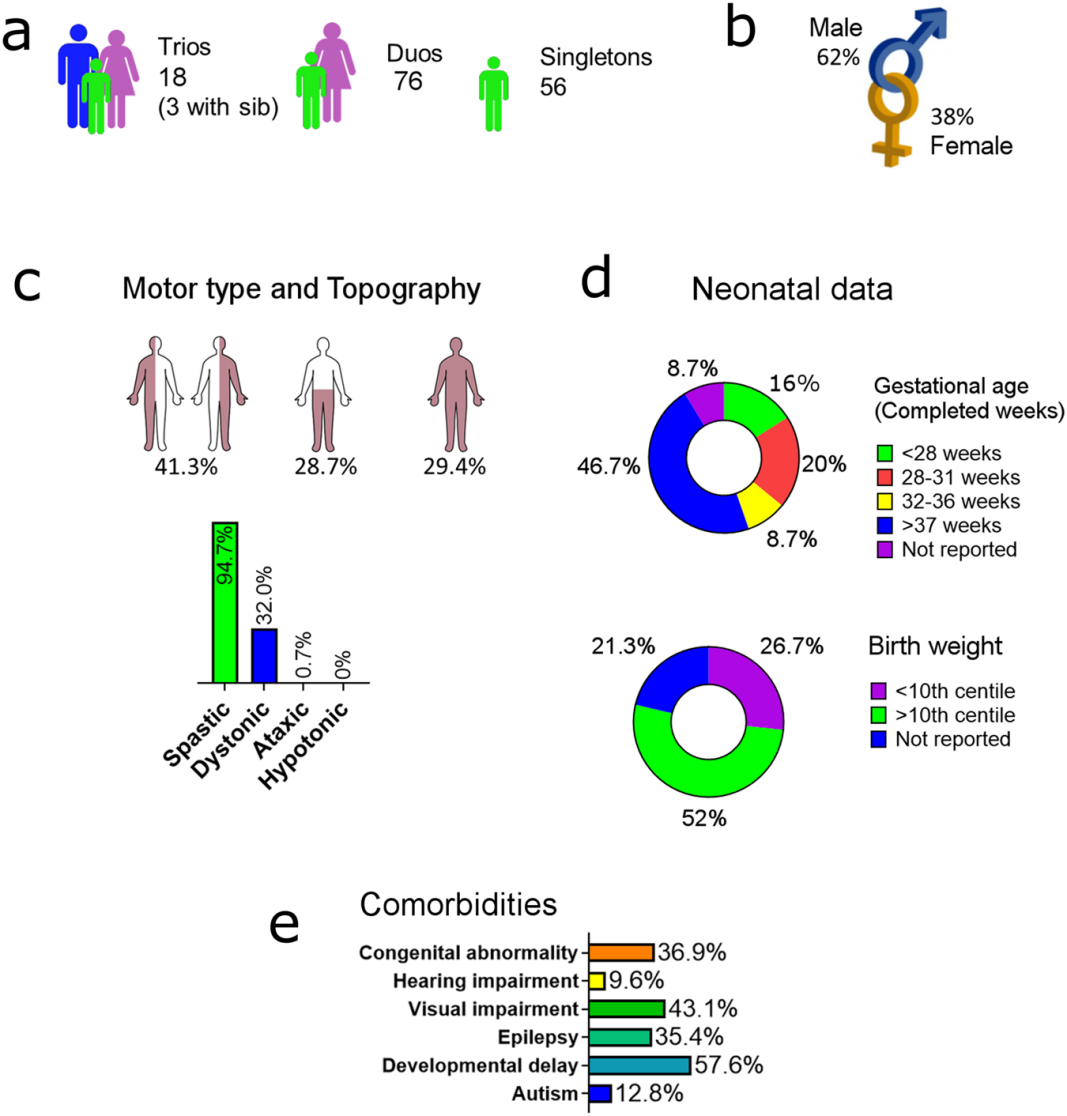
Summary of clinical characteristics of the study cohort. Clinical descriptions for each individual in the cohort are in Supplementary Table 1. **a** Availability of parental samples. **b** Sex. **c** *Motor type and Topography* (*n* = 150): Cases were classified according to topography categories: hemiplegia, diplegia or triplegia/quadriplegia. For motor type, cases with any report of dyskinetic/dystonic motor type are reported as dystonic. **d** *Neonatal data:* Gestational age was confirmed by case note review (*n* = 137). Personalised birth weight centiles for this cohort were calculated using the Gestation Related Optimal Weight (GROW) software^67^ which accounts for individual physiological pregnancy variables (*n* = 118). **e** *Comorbidities:* Comorbidities were reported by parents and confirmed by clinical review where possible. Developmental delay reported in this cohort may include intellectual delays, as well as motor and other developmental delays. Data available: Autism *n* = 113, Developmental delay *n* = 130, Epilepsy *n* = 124, Visual impairment *n* = 116, Hearing impairment *n* = 110, Congenital abnormality *n* = 117.

We performed PCR-free whole GS on singleton cases, achieving mean coverage of 34.64× per base (range 27.47–48.58), with a mean of 94.35% of bases achieving at least 20× coverage (range 87.71–97.73) (Supplementary Fig. 1). Single nucleotide variants and copy number variants were called from these data and filtered and prioritised variants were validated by an orthogonal method (see methods). Inheritance pattern of variants was also determined where parental DNA was available. Of 150 cases sequenced in this study, 18 individuals were from complete parent–child trios, 76 individuals were from parent–child duos (62 maternal and 13 paternal) and 56 were singletons. Of the 18 trios, three trios also had DNA available for at least one sibling. Clinical characteristics of the cohort are summarised in Fig. 1, with detailed clinical description for each individual in Supplementary Table 1.

### Yield of clinically significant variants

We prioritised rare, predicted deleterious variants, as well as variants previously reported as pathogenic/likely pathogenic (P/LP) in ClinVar for follow-up (complete list of prioritised variants, Supplementary Table 2). Prioritised variants were further classified according to ACMG criteria to P/LP variants (Methods). In total, ACMG P/LP variants predicted to contribute to CP were identified in 37/150 individuals (24.7% of the cohort) (Tables 1 and 2, Supplementary Table 3). Of these 37 cases, 7 individuals (4.7% of the total cohort) carried P/LP copy number variants and 30 cases (20% of the total cohort) P/LP SNVs or indels.

**Table 1:** Summary of P/LP variants identified by whole genome sequencing.

Case ID	Gene	Variant/Inheritance	Pop Freq	CADD	ACMG class.	Clinical significance
Individuals with Pathogenic/Likely pathogenic variants considered causative for CP						
P169	*GRIN2B*	NM_000834.3: c.1739T > A: p.F580Y. Het, inheritance unknown	0	29.8	Likely pathogenic	Likely pathogenic
P176	*ARHGAP31*	NM_020754.2: c.1699del: p.P567Rfs^*^28. Het, maternal	0	.	Likely pathogenic	Likely pathogenic
P178^a^	*MFN2*	NM_001127660.1: c.2220 G > A: p.W740^*^. Het, paternal	0	54	Likely pathogenic	Likely pathogenic
P185	*CLCN2*	NM_001171088.3: c.1598 G > A: p.R533Q. Het, inheritance unknown	2.00E−04	24	Likely pathogenic	Likely pathogenic
	*CACNA1C*	NM_000719.7: c.3568 G > T: p.V1190L. Het, inheritance unknown	1.22E−05	27.6	Likely pathogenic	
P205	*CFTR*	NM_000492.4: c.1520-22del: p.P508del. Hom	7.17E−03	21.3	Pathogenic	Pathogenic, diagnosed CF.
	*CLCN1*	NM_000083.3: c.2680 C > T: p.R894^*^. Het, not maternal	3.18E−03	35	Pathogenic	
P217	*SPAST*	Chr2:32347645_32354557del. Compound het, paternal (Supplementary Fig. 6)	0	.	Pathogenic	Pathogenic
		NM_014946.4: c.131 C > T: p.S44L. Compound het, maternal	4.54E−03	21.2	SPG4 modifier	Modifier
P225^a^	*SPAST*	NM_014946.3:c.1625A > G: p.D542G. Het, maternal, sibs share	4.13E−04	21.2	Likely pathogenic	Likely pathogenic
P233	*SYNE2*	NM_015180.4:c.16153 C > T: p.Q5385^*^. Het, inheritance unknown	0	44	Pathogenic	Pathogenic
P236	*TUBB4A*	NM_006087.4: c.1228 G > A: p.E410K. De novo	0	27.5	Pathogenic	Pathogenic
P708	*KLHL3*	NM_001257195.1: c.1446 G > A: p.W482^*^. Het, not maternal	0	38	Pathogenic	Likely pathogenic
P712^a^	*NF1*	Chr17:28992701_30408700del. Het, inheritance unknown	0	.	Pathogenic	Pathogenic
P715	*TTN*	NM_001267550.2:c.50473 C > T:pQ16825^*^. Het, maternal	0	61	Pathogenic	Likely pathogenic
P750^b^	*VCX3A, STS, PNPLA4*	ChrX:6451301_8138000del. Hemi, inheritance unknown (Supplementary Fig. 5)	0	.	Pathogenic	Pathogenic
P754	*KIDINS220*	NM_020738.2: c.4497del: p.R1499Sfs^c^9. Het, not maternal.	0	.	Likely pathogenic	Likely pathogenic
P756	*CACNA1A*	NM_001127222.2:c.7249 G > T: p.E2417^*^. Het, not maternal	0	40	Likely pathogenic	Likely pathogenic
P759	*SETX*	NM_015046.7: c.5821_5830del: p. A1941Lfs^*^6. Het, not maternal.	0	.	Pathogenic	Likely pathogenic
P763	*CPA6*	NM_020361.5: c.799 G > A: p.G267R. Het, inheritance unknown	2.06E−03	29.1	Likely pathogenic	Likely pathogenic
		NM_020361.5: c.619 C > G: p.Q207E. Het, inheritance unknown	1.43E−03	26.2	Likely pathogenic	
	*STRADA*	NM_001003787.4: c.95-2 A > C splicing. Het, inheritance unknown	8.23E−06	25.8	Likely pathogenic	Uncertain significance
P784	*GNB1*	NM_002074.5: c.239 T > C: p.I80T. Het, inheritance unknown	3.98E−06	25.6	Likely pathogenic	Pathogenic
P792	*COL4A1*	NM_001845.4: c.1258 G > A: p.G420R. Het, inheritance unknown	0	18.3	Likely pathogenic	Likely pathogenic
P910	*TUBA1A*	NM_006009.3: c.50 G > A: p.G17D. Het, inheritance unknown	0	29.2	Likely pathogenic	Pathogenic
P911	*COL4A2*	NM_001846.2: c.3625 G > A: p.G1209R. Het, paternal	0	24.7	Likely pathogenic	Likely pathogenic
P931	*ALDH3A2*	NM_000382.3: c.941_943delinsGGGCTAAAAGTACTGTTGGGG:p.A314_P315delinsGAKSTVGA. Hom, IBD	0	.	Pathogenic	Pathogenic
P939	*PDGFRB*	NM_002609.3: c.2083 C > T: p.R695C. Het, maternal	1.13E−04	32	Likely pathogenic	Both likely pathogenic
	*PROC*	NM_000312.3: c.226 G > A: p.V76M. Het, not maternal	4.96E−05	21.2	Likely Pathogenic	
P965	*COL4A1*	NM_001845.4: c.4114 G > C: p.G1372R. Het, inheritance unknown	0	23.9	Pathogenic	Pathogenic
P972	*MT‐TL1*	NC_012920.1: m.3243 A > G. Heteroplasmy 58%, Low level detectable in maternal sample	.	.	Pathogenic	Pathogenic
P980	*22q11.2 dup*	Chr22:18873001_21469900dup. Validated by array (Supplementary Fig. 3)	0	.	Likely pathogenic	Pathogenic
P1110	*COL4A2*	NM_001846.2: c.957 + 2 T > C Splicing. Het, not maternal	0	25	Likely pathogenic	Likely pathogenic
P1138	*COL4A2*	NM_001846.2: c.4049 G > A: p.G1350D. Het, maternal	0	25.6	Likely pathogenic	Likely pathogenic

Detailed interpretations of variants are shown in Supplementary Table 3. Forty-nine individuals had variants classified as pathogenic or likely pathogenic by ACMG criteria. For twenty-eight of these individuals, the clinically reportable variant was considered likely to be causative for cerebral palsy, while a further nine individuals carried variants considered to be risk factors for cerebral palsy (Table 2). Pop. Freq, population frequency of variant in genome aggregation database (gnomAD) or Medical Genome Reference Bank (MGRB).

*CADD* combined annotation dependent depletion scaled score (phred-like), *CF* cystic fibrosis, *del* deletion, *dup* duplication, *hemi* hemizygous, *het* heterozygous, *hom* homozygous, *IBD* identity by descent, *SPG4* Spastic paraplegia 4, *translation termination codon.

^a^Additional high impact candidate variants identified in this individual (see Supplementary Table 4).

^b^Additional compound heterozygous variants identified in this individual (see Supplementary Table 6).

**Table 2:** Summary of risk variants, variants of uncertain significance and incidental findings identified by whole genome sequencing.

Case ID	Gene	Variant/Inheritance	Pop Freq	CADD	ACMG class.	Clinical significance
*Individuals with P/LP variants considered risk factors for CP*						
P165	*TRIM32, ASTN2*	Chr9:119311659_119462832del. Het, inheritance unknown (Supplementary Fig. 2)	0	.	Likely pathogenic	Risk factor
	*HTT*	NM_002111.8: c.7731 G > A: p.W2577^*^. Het, inheritance unknown	0	52	Pathogenic	Uncertain significance
P199^a^	*F8*	NM_000132.3: c.5146 C > A: p.H1716N. Inheritance unconfirmed, father haemophilia A	0	25.5	Likely pathogenic	Risk factor
P214	*15q11-q13 dup*	Chr15:22722801_26749200dup. Likely maternal origin by methylation (Supplementary Fig. 4), inheritance unknown	0	.	Pathogenic	Risk factor
P710	*F2*	NM_000506.5: c.^*^97 G > A (G20210A, rs1799963). Het, inheritance unknown	8.44E−03	.	Likely pathogenic	Risk factor
P747	*F2*	NM_001311257: c.^*^97 G > A (G20210A, rs1799963). Het, inheritance unknown	8.44E−03	.	Likely pathogenic	Risk factor
P752	*NKX2-6*	NM_001136271.3: c.455dup: p.Q153Afs^*^207. Het, maternal	3.15E−05	30	Pathogenic	Risk factor
P779	*F2*	NM_000506.3: c.598 G > A: p.E200K. Het, inheritance unknown	1.20E−03	0	Risk factor	Risk factor
P795	*F2*	NM_001311257: c.^*^97 G > A (G20210A, rs1799963). Het, inheritance unknown	8.44E−03	.	Likely pathogenic	Risk factor
P1147	*Chr1q21.1 deletion*	Chr1:145382601_145616000del. Het, inheritance unknown (Supplementary Fig. 7)	0	.	Pathogenic	Risk factor
*Individuals with P/LP variants of uncertain clinical significance for CP*						
P182	*GALC*	NM_000153.4: c.1592 G > A: p.R531H. Het, paternal	3.24E−05	29.4	Pathogenic	Uncertain significance
		NM_000153.4: c.334 A > G: p.T112A. Het, not paternal	2.50E−03	23	Likely pathogenic	
P188	*COL4A4*	NM_000092.5: c.4720 C > T: p.Q1574^*^. Het, inheritance unknown	0	46	Pathogenic	Uncertain significance
P228^a^	*ASTN2*	NM_014010.4: c.2317 C > T: p.Q773^*^. Het, paternal	0	46	Pathogenic	Uncertain significance
P232	*EGFR*	NM_005228.3: c.925 C > T: p.R309^*^. De novo, not shared by 3 siblings	0	38	Pathogenic	Uncertain significance
P703	*SPG7*	NM_003119.2: c.1045 G > A: p.G349S. Het, inheritance unknown	8.23E−04	26.7	Likely pathogenic	Uncertain significance
P760	*PNPLA6*	NM_001166114.2: c.3058_3061dup: p.R1021Qfs^*^38. Het, inheritance unknown	1.25E−04	35	Pathogenic	Uncertain significance, likely in *cis* by long-read sequencing
		NM_001166114.2: c.1144del: p.A383Pfs^*^11. Het, inheritance unknown	0	.	Pathogenic	
P778	*VWF*	NM_000552.4: c.2561 G > A: p.R854Q. Het, inheritance unknown	3.47E−03	33	Pathogenic	Uncertain significance
P1106	*PIEZO2*	NM_022068.2: c.1444del: p. R482Efs^*^16. Het, not maternal, not shared by sibling	0	.	Likely pathogenic	Uncertain significance
P1132	*BUB1B*	NM_001211.5: c.1526 C > A: p.S509^*^. Het, maternal	0	36	Pathogenic	Uncertain significance
*Individuals with P/LP variants considered incidental findings for CP*						
P741	*MITF*	NM_006722.2: c.1018 C > T: p.R340C. Het, inheritance unknown	0	33	Likely pathogenic	Incidental finding
P746	*ABCA4*	NM_000350.2: c.6316 C > T: p.R2106C. Het, inheritance unknown	1.31E−04	33	Pathogenic	Incidental finding
		NM_000350.2: c.5282 C > G: p.P1761R. Het, inheritance unknown	0	26.5	Likely pathogenic	Incidental finding
P749^a^	*GJB2*	NM_004004.5: c.95 G > A: p.R32H. Het, paternal	3.99E−06	25.3	Pathogenic	Incidental finding
P802	*EFEMP1*	NM_001039348.3: c.1033 C > T: p.R345W. Het, not maternal	0	25.9	Pathogenic	Incidental finding

Detailed interpretations of variants are shown in Supplementary Table 3. Nine individuals carried variants considered to be risk factors for cerebral palsy, with a further ten individuals carrying at least one variant classified as likely pathogenic/pathogenic by ACMG criteria, but with uncertain clinical significance in the individual. A further four individuals carried ACMG pathogenic/likely pathogenic variants which were considered incidental findings likely contributing to other components of the clinical phenotype, but without a link to CP. Pop. Freq, population frequency of variant in genome aggregation database (gnomAD) or Medical Genome Reference Bank (MGRB).

*CADD* combined annotation dependent depletion scaled score (phred-like), *del* deletion, *dup* duplication, *het* heterozygous, *translation termination codon.

^a^Additional high impact candidate variants identified in this individual (see Supplementary Table 4).

An additional four individuals carried variants which were classified as ACMG P/LP and of likely clinical significance based on the reported clinical presentation, but considered unlikely to explain the CP phenotype (Table 2, incidental finding). A further nine individuals carried variants classified as ACMG P/LP but with uncertain contribution to the clinical presentation in the individual (Table 2, uncertain significance). These variants included: a paternally inherited stop-gain variant in *ASTN2*, copy number variants of which are a risk factor for NDDs including autism^17^; a maternally inherited pathogenic variant in *BUB1B*, variants in which are associated with premature chromatid separation trait and increased spontaneous abortions in female carriers; compound heterozygous *GALC* variants (p.R508H/p.T89A) in a girl lost to further follow up, reported with spastic diplegia, progressive dystonia and ataxia, with early imaging demonstrating abnormalities in both putamina, and metabolic testing showing GALC enzyme activity in blood just within normal range (2.1, normal range 2.0–30 pmol/min/mg protein); a novel heterozygous frameshift variant in *PIEZO2*, loss-of-function variants in which have been associated with autosomal recessive disorder Distal Arthrogryposis with impaired proprioception and touch (MIM 617146); a heterozygous pathogenic *COL4A4* variant in a girl with reported oligohydramnios and IUGR, also lost to follow-up; and a novel de novo truncating variant in *EGFR* which has not been associated previously with neurodevelopmental phenotypes. In addition, we identified two individuals carrying known pathogenic *BRCA1* variants which were reported to families as incidental findings (P710, P751).

Of the 37 cases with a variant interpreted as causing CP, 11 cases (7.3% of the cohort) harboured a missense variant in a gene associated with an autosomal dominant disorder (*COL4A1* and *COL4A2* (two individuals each), *F2, GRIN2B, CLCN2, SPAST, TUBB4A, GNB1, TUBA1A)* and a further 13 (8.7% of the total cohort) carried predicted loss-of-function variants in genes associated with autosomal dominant disorders (*ARHGAP31*, *CACNA1A*, *MFN2*, *SYNE2*, *KLHL3*, *TTN, NKX2-6, SETX, KIDINS220, COL4A2, F2* (three individuals)), making autosomal dominant disorders (inherited or de novo) the predominant finding in this cohort, accounting for 64.9% of diagnoses. There was no significant enrichment for P/LP variants causing CP in complete parent–child trios (7/18) compared to the whole cohort (37/150) (hypergeometric distribution: 1.58-fold enrichment, cumulative probability *p* value = 0.12), with only 1/18 trio cases having a confirmed de novo P/LP variant. Enrichment analysis with the gene-set enrichment tool ShinyGO v0.61^18^ ranked HPO terms “Autosomal Dominant Inheritance” and “Variable Expressivity” as the first and third most highly enriched terms associated with the list of diagnostic genes (*p* = 5.3E−16 and *p* = 1.6E−07 respectively), suggesting that inherited pathogenic variants may have an under-appreciated contribution to CP causation.

Autosomal recessive disorders were a relatively rare cause of CP in this cohort, with one individual carrying two heterozygous variants in *CPA6* previously reported in one child with familial temporal lobe epilepsy^19^ but with family samples unavailable for segregation, and one child identified with a homozygous in-frame deletion in *ALDH3A2* resulting from a first cousin union (Table 1). In addition, one individual demonstrated heteroplasmy for *MTTL1* 3243 A > G (estimated 58% frequency in blood) which is the most common mtDNA mutation associated with Mitochondrial Encephalomyopathy, Lactic Acidosis, and Stroke-like episodes (MELAS, MIM 540000). Three individuals displayed likely blended phenotypes, with P/LP variants identified in more than one gene (*CLCN2* and *CACNA1C*, *CFTR* and *CLCN1, PDGFRB* and *PROC)*. In addition, a female obligate *F8* mutation carrier was found to have complete skewing of X-inactivation on a background of prematurity and maternal pre-eclampsia. XCI skewing has been recently demonstrated to correlate with F8 activity in heterozygous carriers of pathogenic variants^20^. A novel likely pathogenic SNV in *F8* was identified in this girl (p.H1716N), however we were unable to obtain paternal DNA to confirm inheritance. Case note review for this patient identified mildly increased activated partial thromboplastin time, with low-normal F8 levels at 5 years of age.

### Yield of variants of uncertain significance in variation intolerant genes

After filtering data for variants with frequency <1.0 × 10^−4^ in gnomAD and CADD score >20, we prioritised variants based on in silico predictions of pathogenicity and previous association with neurodevelopmental disorders (see methods). We also considered rare loss-of-function variation in highly intolerant genes without known disease association, since these may represent novel candidates for CP aetiology. An additional 52/150 individuals (34.7%) with no other ACMG P/LP variant explaining the clinical phenotype carried rare, high impact variants in 58 variation intolerant genes (Supplementary Table 4). We defined these as variants in genes with missense *Z* score >2 in gnomAD, or observed/expected ratio <0.5 for loss-of-function variants. Of these 58 genes, 41/54 genes located on autosomes are predicted likely or very likely to be associated with dominant disorders according to machine learning tool DOMINO^21^. Variants in six genes were identified in two or more individuals each: *CAMTA1* (three individuals), loss-of-function mutations in which cause Cerebellar ataxia, non-progressive, with mental retardation (MIM 614756); *PIEZO2* (three individuals) which is associated with a range of clinically overlapping disorders including Marden–Walker Syndrome (MIM 248700) and Arthrogryposis, distal, type 3 and type 5 (MIM 114300 and 108145); *NOTCH3*, mutations in which cause Cerebral Arteriopathy with subcortical infarcts and leukoencephalopathy (CADASIL, MIM 125310); *SPTBN2*, mutations in which cause Spinocerebellar ataxia 5 (MIM 600224); *CHD8*, which is recurrently mutated in autism cohorts^22^ and has been associated with a range of neurodevelopmental phenotypes;^23^ and *ROCK2*, disruption of which causes IUGR and foetal death in a mouse model^24^. A further five individuals who carried a variant classified as ACMG P/LP carried at least one additional high impact variant in a variation intolerant gene (Tables 1 and 2).

### Risk factor profiles in individuals with clinically significant genetic variants

**Fig. 2:**
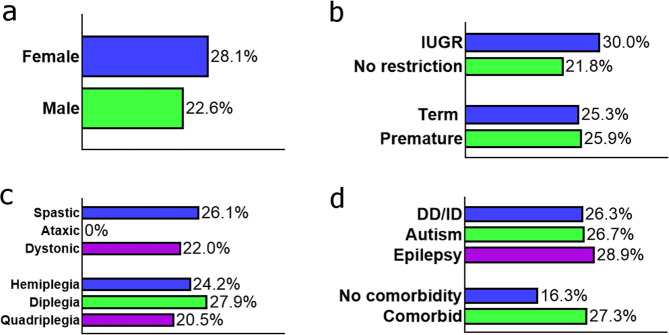
Percentage of cases with a clinically reportable variant by clinical characteristic. For each clinical characteristic, only individuals for whom data were available are shown, with percentages calculated based on individuals having a variant interpreted as P/LP and causative or a risk factor for CP. *Sample sizes*: **a** *Sex***:** Male, *n* = 93, Female, *n* = 57; **b** *Neonatal data***:** Premature, *n* = 54; Term, *n* = 83; Intrauterine growth restriction (IUGR), *n* = 78; No growth restriction, *n* = 40; **c** *Motor type and topography:* Quadriplegia/triplegia, *n* = 44; Diplegia, *n* = 43; Hemiplegia, *n* = 62; Dystonic, *n* = 41; Ataxic, *n* = 1; Spastic, *n* = 142; **d** *Comorbidities:* Co-morbid, *n* = 88; No comorbidity, 49; Autism, *n* = 15, Developmental delay, *n* = 76, Epilepsy, *n* = 45.

We sought to assess the yield of clinically significant genetic variants (Tables 1 and 2) in individuals with differing underlying risk factor profiles, in order to identify clinical indicators for genetic testing. A summary of the percentage of cases with a clinically reportable variant by clinical characteristic can be found in Fig. 2. Testing each variable independently and as part of all possible combinations in a logistic model found no single risk factor or combination of risk factors with significant probability of predicting genetic diagnosis in this cohort (Adjusted odds ratios from logistic regression: sex 0.67 (95% CI 0.29–1.58), gestation 0.97 (95% CI 0.90–1.04), IUGR 1.00 (95% CI 0.99–1.01)). We similarly tested for any association between these risk factors and identification of a variant in an intolerant gene and still found no significant association (data not shown). We also considered the type of brain imaging findings reported in cases with a genetic diagnosis compared to the whole cohort (Supplementary Fig. 9; Classifications: no specific pathology, cerebral infarction, periventricular leukomalacia/porencephaly without hydrocephalus, intraventricular haemorrhage, major abnormality, infection, hypoxic ischaemic encephalopathy, imaging not available). We found no significant difference in distribution of brain imaging findings in cases with a genetic diagnosis compared to the whole of cohort (chi-square statistic: 13.78, *p* = 0.088), however this analysis was limited by the small sample size, as well as inconsistencies in timing and type of imaging performed across the cohort.

### Association between genetic diagnosis and neurodevelopmental comorbidities

Data for comorbidities was available for 137/150 individuals in this cohort. No single comorbidity (autism, developmental delay/intellectual impairment, epilepsy) was independently associated with an increased likelihood of a genetic diagnosis in this cohort (chi-square statistic: autism 2.25, *p* = 0.32, developmental delay 2.93, *p* = 0.23, epilepsy 0.36, *p* = 0.83). We performed logistic regression to test the influence of the number of comorbidities in an individual (coded as a continuous trait, with 0 for no comorbidity, up to 3 for all of autism, developmental delay and epilepsy) on the probability of finding a genetic diagnosis in this cohort. Yield of P/LP varied from 8/49 (16.3%) of cases with no reported comorbidities, up to 2/7 (28.6%) of cases with three reported comorbidities, however there was no significant increase in probability of finding a P/LP variant with increasing number of comorbidities detectable in this cohort (OR 1.23, CI 0.80–1.89, *p* = 0.35). Probability of having either a P/LP variant (Tables 1 and 2, clinical interpretation P/LP, risk factor) or variant in an intolerant gene (Supplementary Table 4) increased with increasing number of comorbidities (OR 1.68 CI 1.11–2.54, *p* *=* 0.02). Together with the observation that many rare variants identified in variation intolerant genes were inherited from an apparently unaffected parent (Supplementary Table 4), this suggests that inherited, rare deleterious genetic variation may contribute to CP aetiology.

### Variants associated with stroke risk and heart defects are a frequent finding in cerebral palsy

Along with six individuals carrying P/LP variants in genes involved in hemostatic pathways (PROC, F2 (four cases) and F8), we identified a further five individuals with P/LP genetic variants in type IV collagens *COL4A1* (two cases) and *COL4A2* (three cases), variants in which cause autosomal dominant Brain Small Vessel Disease (MIM 175780) and susceptibility to intracerebral haemorrhage^25^. Four of these variants impacted glycine residues in the Gly-X-Y repeats in the triple helical domain of the COL4A1/2 proteins, while the final variant (COL4A2 c.957 + 2 T > C) is predicted to result in loss of the wild type splice donor site of exon 16, and therefore likely also impacts on the downstream triple helical domain.

An additional three individuals were found to harbour pathogenic variants in genes associated with vascular abnormalities or congenital heart defects (*TTN*, *NKX2-6* and *ARHGAP31*). We identified a novel, high-impact, maternally inherited stop-gain variant located at the I-band/A-band junction of *TTN* in a boy with complex congenital heart disease, epilepsy, mild intellectual impairment and behavioural problems with spastic/dystonic hemiplegia as a sequela of a stroke at 3 weeks of age following cardiac surgery. Loss-of-function variants in *TTN* have been associated with congenital heart disease and cardiac arrhythmia, which increases risk of stroke^26^. We also considered this variant as an incidental finding in the mother of the proband.

Another individual was identified who carried a rare, maternally inherited frameshift variant in *NKX2-6* (p.Q153Afs*207) and was reported with spastic hemiplegia with no documented comorbidities. A nonsense variant affecting the adjacent amino acid (p.K152*) has been reported in homozygous state to cause conotruncal heart malformations^27^, while a missense mutation in the preceding amino acid (p.F151L) was reported to segregate with persistent truncus arteriosus in one family^28^ highlighting an essential role for the homeodomain of this cardiac transcription factor. Heterozygous missense and nonsense mutations within the homeodomain have also been associated with both congenital heart disease^29^ and autosomal dominant predisposition to atrial fibrillation^30^ suggesting that mutations in *NKX2-6* are a risk factor for thromboembolism.

We also identified one individual with a maternally inherited frameshift variant in *ARHGAP31* (p.P567Rfs*28). Loss-of-function variants in *ARHGAP31* cause Adams Oliver syndrome (AOS, MIM 100300) which is defined by aplasia cutis congenita of the scalp vertex and terminal transverse limb defects, but is also frequently associated with diverse vascular anomalies, congenital heart defects and neurological sequelae. Variable penetrance and expressivity has been described within families, including reports of cases presenting with stroke in childhood^31^ and also some unaffected obligate carriers^32,33^. We were unable to re-contact the family to determine whether scalp or limb defects were present in the proband or mother. Taken together, we interpret this variant as pathogenic, based on reported loss-of-function variants in the same protein regions causing AOS, with an atypical presentation in the proband primarily characterised by neurological sequelae (seizures, intraventricular haemorrhage resulting in hemiplegia, porencephalic cyst, global developmental delay and mild autism) resulting from a vascular anomaly on a background of premature birth.

### Variants in hereditary spastic paraplegia genes are a major contributor to cerebral palsy aetiology

We reviewed genes associated with Childhood onset Hereditary Spastic Paraplegia (HSP) in PanelApp (https://panelapp.genomicsengland.co.uk/panels/568/ HSP, childhood onset, Panel version 2.18, green rating). Of 37 cases with a P/LP variant of clinical significance, 5 cases (13.5%) were resolved by a variant or variants in an HSP gene. These genes are *SPAST* (two cases), *TUBB4A*, *KIDINS220* and *ALDH3A2*. All five individuals met criteria for a diagnosis of CP at 4 years of age, providing a clear basis for inclusion of HSP genes in any diagnostic gene panel for CP.

A further five individuals carried a reported P/LP variant in a gene responsible for autosomal recessive HSP (*SPG7, SPG11, PNPLA6*), with either no second variant identified, or variants determined to be in *cis* (Supplementary Table 5). Some individuals with apparently dominant inheritance of *SPG7* variants causing HSP or Amyotrophic lateral sclerosis have been described previously^34,35^, including individuals carrying the *SPG7* p.G349S and p.A510V variants identified in this cohort, however this association remains controversial since both variants are relatively frequent in population databases (gnomAD frequencies 8.23E−04 and 2.90E−03 respectively).

### Clinical utility of genetic testing in cerebral palsy

We assessed clinical utility of genetic findings in this cohort by identifying cases where this knowledge would be likely to lead to altered care pathways for the participant (Supplementary Table 7). Of 37 individuals with a variant or variants classified as causing or contributing to CP risk, we identified 20 individuals (54.1%) who may be eligible for a clinical trial, have access to an approved drug, or for whom a change in management would be considered as a result of their molecular diagnosis.

## Discussion

In this study, we sought to assess the diagnostic yield in a highly clinically heterogeneous cohort of individuals with a formal diagnosis of CP. Approximately 1/4 individuals in this unselected, genetically naïve cohort have a P/LP variant of clinical significance according to ACMG-AMP guidelines. Genetic findings include variants causing a diverse range of genetic disorders with spasticity or dystonia as a component of the described clinical presentation, mutations in genes in the coagulation cascade, and genetic disorders for which CP represents an expansion of the known clinical spectrum. Considering cases by aetiological groups, we classified genetic causes of CP into three broad subtypes: variants predisposing to stroke, variants associated with HSPs and variants associated with other NDDs (Table 3). Considering cases by these aetiological classifications, we found no significant difference in distribution of brain imaging findings in any of these groups compared to the whole cohort (chi-square statistic stroke/cardiovascular 2.53, *p* = 0.96; HSP 14.52, *p* = 0.07; Neurodevelopmental disorder 10.45, *p* = 0.24).

**Table 3:** Aetiological sub-types of genetic cerebral palsy identified in this study.

Aetiology	Causes	Associated genes
Stroke/cardiovascular	Thrombophilic pathway Small vessel disease Congenital heart defects Vascular abnormalities	*F8, F2, PROC/PDGFRB*, *COL4A1, COL4A2*, *TTN, NKX2-6, ARHGAP31*, *KLHL3, NF1, CLCN1/CFTR, CLCN2/CACNA1C*
Hereditary spastic paraplegias	Diverse genetic causes	*SPAST, TUBB4A, KIDINS220, ALDH3A2*
Neurodevelopmental disorders	Brain malformations Ion channel defects Metabolic defects CNVs with NDD risk	*GNB1, TUBA1A, GRIN2B, CPA6, MT-TL1, MFN2, SYNE2, SETX, CACNA1A*, *9q33.1 del, 15q11-q13 dup*, *Xp22.3 del, 22q11.2 dup*, *1q21.1 del*

Presence of comorbidities (autism, developmental delay/intellectual impairment, epilepsy) was not predictive of a genetic diagnosis in this cohort, either individually or in combination. A recent retrospective analysis of genetic findings in 1526 patients with CP reported a significant enrichment for genetic findings in individuals with intellectual disability (ID) plus CP, compared to CP without ID, as well as an increasing diagnostic rate with increasing number of comorbidities^36^. The majority of this cohort (1345 cases) were sequenced following referral for clinical sequencing, with 92% of this group also having ID or developmental delay reported, and therefore were already suspected of having a genetic diagnosis at the time of sequencing. Sequencing of larger unselected cohorts will be required to determine whether this association holds true for a clinically representative sample of CP. We also found no association between risk factors: sex, gestational age, birth weight centile, or any combination of these risk factors and likelihood of genetic diagnosis. While our cohort size is not adequate to detect small differences in risk factor profile, these data suggest that, in practice, selection of cases for genetic sequencing based on the presence or absence of measurable risk factors will miss a proportion of genetic diagnoses.

P/LP variants in genes typically associated with HSP were a frequent finding in this cohort (13.5% of diagnoses, *SPAST* (two cases), *TUBB4A*, *KIDINS220* and *ALDH3A2*), confirming previous observations of the clinical overlap between early onset HSP and CP^12,37,38^ and adding to the growing list of HSP genes contributing to the genetic landscape of CP^36,39^. These findings also underline the value of genetic testing in families where other family members may be at risk of a later onset disorder. None of the families in our cohort had a history of spastic paraplegia and the clinical phenotype of all cases was considered to be stable at 4 years of age when inclusion on Australian CP registers is typically confirmed. For two cases, a suspicion of HSP was suggested on review by a paediatric neurologist. For one of these cases (*TUBB4A* p.E410K) clinical review was performed to determine eligibility for selective dorsal rhizotomy surgery at 4 years, 6 months of age. For the other (SPAST del ex7-8/p.Ser44Leu), suspicion of HSP was suggested at 12 years of age based on gait. For both families, the diagnosis of HSP had important clinical implications: with a decision not to undergo surgical intervention resulting for the former, and cascade testing of other family members for the latter. Suspicion of HSP or progressive features were not noted for the remaining three cases at last review at age 6 years (P225), 15 years (P931) and 16 years (P754), highlighting the difficulty in differential diagnosis of HSP and CP in the absence of marked early degeneration. Early genomic testing has the capacity to provide precision diagnosis and prognosis much earlier than longitudinal follow-up, facilitating earlier implementation of appropriate interventions.

Fourteen of the thirty-seven individuals with a genetic diagnosis (37.8%) were found to carry variants in genes associated with stroke risk, confirming this to be a major mechanism in CP. Of particular note, five individuals (3.3% of the total cohort) had P/LP variants in *COL4A1/COL4A2*. Collagen Type IV proteins are a major structural component of basement membranes, providing strength and support to tissues, including blood vessels, as well as playing roles in development and cell signalling^40^. Variants in *COL4A1* and *COL4A2* have been reported in patients diagnosed with CP^41–44^, however genetic testing was generally only performed upon evidence of recurrent stroke or evolution of multi-systemic clinical phenotypes. Together with exome sequencing and targeted sequencing^7,14^, a total of seven individuals with P/LP variants in *COL4A1/2* have been identified in the Australian CP Biobank (individuals tested: *n* = 333 individuals for *COL4A2* and *n* = 604 for *COL4A1*), with a range of clinical signs, including hydrocephalus with antenatal IVH, porencephalic cysts, epileptic encephalopathy, periventricular leukomalacia, and schizencephaly with polymicrogyria and associated periventricular gliosis. Genetic diagnosis in these families is of benefit not only for surveillance of index cases for recurrent stroke, but to other carrier family members who are also at higher risk of cerebrovascular disease. Prenatal genetic testing can also be offered for subsequent pregnancies, since delivery by caesarean section can reduce the risk of perinatal haemorrhage.

In this cohort, a number of variants in known disease genes were found to be inherited from an apparently unaffected parent. We validated variants in parental samples using at least one orthogonal method and did not observe properties suggestive of mosaicism, however we cannot rule out mosaicism in parents for variants validated by Sanger sequencing. Pathogenic variation in genes with variable penetrance, age-of-onset or expressivity may also contribute to the genetic burden in CP, with other risk factors, including pregnancy and birth complications, contributing to the poor developmental outcome in the proband and culminating in an early onset or more severe disorder. For example, variants in *COL4A1* have been suggested to play a role in susceptibility to intraventricular haemorrhage in the presence of other risk factors^45^, resulting in variable severity, onset or incomplete penetrance in some families. Furthermore, maternal polymorphisms in *COL4A1/COL4A2*^46^ as well as genes associated with thrombophilia^47^ have been associated with pregnancy complications including pre-eclampsia, IUGR and placental abruption, suggesting that maternal carrier status for some variants may in itself contribute to intrauterine environmental stresses and therefore be associated with increasing severity of pathology. Determining the contribution of inherited variants of variable penetrance to CP aetiology will require much larger cohort sizes to achieve sufficient statistical power to demonstrate causality, particularly for more common risk alleles with smaller effect size. This phenomenon has been reported in autism, where availability of many thousands of cases has revealed a contribution of both rare transmitted variants^48^ and common polymorphisms^49^ to autism risk.

Despite recruiting participants with a confirmed diagnosis of CP, we have uncovered many surprising genetic diagnoses. These cases represent the genetic heterogeneity in CP cohorts, and therefore are informative for differential diagnosis, particularly where they represent an atypical presentation or a phenotypic expansion for a genetic disorder. Our data show that at least 1/4 of individuals in unselected CP cohorts would benefit from precise clinical genetic diagnoses. Moreover, ~1/8 of the total cohort have findings which may make them eligible for clinical trials, indicate use of already approved drugs, or lead to a change in clinical management. In addition, we have identified a number of cases where there is likely to be previously unsuspected familial risk. This is very likely to be an underestimation of the true diagnostic rate in this cohort, since the unavailability of both parental samples for many cases limited interpretation of VUS. All P/LP variants identified in this analysis would likely have also been detected by the combination of exome sequencing and chromosomal microarray, which are readily available for clinical testing. The value of our GS dataset will be fully realised in enabling identification of novel genetic variation (e.g. deep intronic variants, complex structural variants/rearrangements, repeat sequences), particularly with additional ‘omics analyses such as RNA sequencing and epigenetics aiding interpretation, with the potential to further increase the diagnostic yield. Our study strongly supports implementing genetic testing as a diagnostic and prognostic tool early in patient care when a CP diagnosis is given or suspected.

## Methods

### Study samples

Study samples were obtained from the DNA Biobank of the Australian Collaborative CP Research Group and informed, written consent was given, either by the participant or their guardian, for the use of their sample in CP research. Ethical approval for the Biobank and genetic studies of CP were given by the Adelaide Women’s and Children’s Health Network (WCHN) Human Research Ethics Committee (records HREC/12/WCHN/61 and HREC/15/WCHN/148). Mean age at recruitment was 8.4 years (2.0–18.2 years), with mean age at time of sequencing being 13.8 years (4.3–23.9). 146/150 cases were formally diagnosed according to international consensus criteria^50^ and included on a state based Australian CP Register. CP diagnosis for the remaining 4/150 cases was confirmed by the treating neurologist or paediatric rehabilitation specialist at >4 years of age. All cases in this cohort met internationally accepted inclusion criteria for diagnosis of CP^50^, including their condition being considered permanent and non-progressive at >4 years of age. Cases were not clinically selected for sequencing, but are a convenience cohort mostly recruited through Botox clinics around Australia. Clinical information was obtained by completion of a questionnaire by the participant or their guardian and clinical review of patient records.

### DNA extraction

Genomic DNA was extracted from patient-derived Lymphoblastoid cell lines at Genetic Repositories Australia (GRA, Sydney, Australia), or extracted from blood or saliva samples at the Australian Genomics Research Facility or in the research laboratories of the Neurogenetics Research Group at the University of Adelaide. Commercially available kits were used for DNA extraction in all cases. DNA integrity and quantity were verified by gel electrophoresis and Qubit dsDNA assay (Life Technologies).

### Whole genome sequencing

Clinical grade PCR-free whole GS was performed as a service by the Kinghorn Centre for Clinical Genomics (KCCG, Garvan Institute of Medical Research, Sydney, Australia). Libraries for sequencing were prepared using the KAPA HyperPrep Kit according to manufacturer’s instructions, with one sample loaded per flow cell lane. Flow cells were loaded onto an Illumina HiSeq X sequencer and 2 × 150 bp paired-end sequencing was performed.

### Single nucleotide variant and indel calling

Single nucleotide variants and indels were called at KCCG as a service using their NATA accredited variant calling pipeline (ISO15189). Briefly, raw data was converted to FastQ file format using Illumina’s bcl2fastq (2.16.0). Paired reads were aligned to the hs37d5 reference genome using BWA-MEM (V0.7.10-r789), followed by Novosort (V1.03.01) to create coordinated-sorted, duplicate marked BAM files. Samtools (V1.1) was used to calculate high level summary statistics. Variant calling was performed using GATK tools (V3.3). Reads that overlap known indels were realigned, using GATK IndelRealigner and base quality scores were recalibrated using GATK BaseRecalibrator. Variants were called for each sample using GATK HaplotypeCaller and gVCF files for each sample were jointly-called with other samples in the same sequencing batch (minimum 40 samples/batch), using GATK GenotypeGVCFs. Variants were then recalibrated using GATK Variant Quality Score Recalibrator, which uses sets of known true and false variants to determine whether there is evidence for a variant at a particular site. Variants ranked as PASS following recalibration were included for filtering and prioritisation.

### Single nucleotide variant and indel filtering

Jointly-called VCFs were annotated using ANNOVAR (database version 2019Mar23). Three alternate strategies were employed to filter for rare deleterious variants (Filter 1), compound heterozygous or autosomal recessive variants (Filter 2) and known pathogenic variants (Filter 3). Variants with allele count >2 in the same sequencing batch were excluded (AF ≤ 0.025). Prioritised variants are listed in Supplementary Tables 2, 5 and 6.

#### Filter 1 (rare deleterious)

All exonic (non-synonymous, stopgain, indel and frameshift variants) and splicing variants as annotated by ANNOVAR were filtered for variant frequency <1.0 × 10^−4^ in gnomAD and ExAC v3 and CADD^51^ Scaled score >20 (where available). Variants were then prioritised for follow-up based on additional evidence (1) ≥ 3 of the following criteria met (where available): SIFT^52^ = D, PolyPhen2 HVAR^53^ = D/P, Mutation Taster^54^ = A/D, MetaSVM^55^ = D, GERP + +RS^56^ > 4; (2) membership in one or more of the following gene panels: PanelApp DDG2P v2.9 (paediatric disorders panel) and in-house epilepsy, intellectual disability and CP gene panels.

#### Filter 2 (autosomal recessive)

All exonic (non-synonymous, stopgain, indel and frameshift variants) and splicing variants as annotated by ANNOVAR were filtered for variant frequency <1.0 × 10^−2^ in gnomAD and ExAC v3 and the resulting variant list was filtered for possible compound heterozygous, autosomal recessive or di-genic variants.

#### Filter 3: (known pathogenic)

All variants, including non-coding variants, were annotated with ClinVar database (version 20170905) and filtered for terms “likely pathogenic” and “pathogenic”. ClinVar records were then manually reviewed to assess available evidence for pathogenicity and relevance to CP and any other reported clinical phenotypes. Where a single pathogenic or likely pathogenic (P/LP) variant in a gene associated with an autosomal recessive genetic disorder was identified, we also looked for additional variants in the gene (including coding, non-coding and copy number variants). The effect of putative splicing variants was predicted using SpliceAI^57^ and Human Splicing Finder^58^.

### SNVs and indel validation

Single nucleotide variants and Indels were validated by Sanger sequencing using BigDye terminator chemistry 3.1 (ABI) and analysed using a 3730xl genetic analyser (Applied Biosystems, Foster City, CA, USA). Primers are listed in Supplementary Table 8. Sequencing data were analysed using DNASTAR Lasergene 10 Seqman Pro8 (DNASTAR, Inc. Madison, WI, USA). Where parental DNA was unavailable, phasing of potential compound heterozygous variants was determined by long-read sequencing of amplicons on an Oxford Nanopore MinION Mk1B (described below).

### Copy number variant (CNV) calling

CNV calling was performed using ClinSV^59^, a copy number variant integration, annotation, prioritisation, and visualisation framework which integrates depth of coverage based variant calls from CNVnator^60^ with split-read and discordant pair based variant calls from Lumpy^61^. Variant calls are annotated with population allele frequencies from the Australian Medical Genomes Reference Bank^62^ (MGRB). Variants with allele frequency of <1% in MGRB (referred to as rare), passing the variant detection threshold and impacting genes were subjected to further filtering.

### Copy number variant filtering

Rare, gene affecting variants which passed the variant detection threshold were prioritised by the following criteria: impacting coding region of gene, overlapping OMIM genes, overlapping candidate gene lists PanelApp DDG2P v2.9 (paediatric disorders panel) and our epilepsy, intellectual disability and CP gene panels. Variants were visually inspected in IGV, prior to validation by at least one orthogonal method. Copy number variants were validated by: PCR and Sanger sequencing for small deletions and insertions (Supplementary Fig. 6), or DNA microarray, quantitative PCR (qPCR), or Multiplex Ligation-dependent Probe Amplification (MLPA) assay for larger genetic changes (described below).

### Classification of variants

Prioritised variants were classified according to recommendations of the ACMG-AMP^63^ into categories: ‘pathogenic’, ‘likely pathogenic’, ‘uncertain significance’, ‘likely benign’ and ‘benign’. Evidence used for variant classification included population frequency, in silico predictions of functional impact, functional data (where the specific variant has been previously reported), inheritance pattern and clinical fit with previously reported phenotypes. Since the majority of cases in this cohort did not have both parents available for validation of inheritance pattern, the weight of evidence in other categories required to meet the threshold for classification as P/LP was greater. Variants inherited from an apparently unaffected parent were only classified as P/LP where evidence for incomplete penetrance/variable expressivity of other variants in the gene have been reported (see Tables 1 and 2), with variants lacking this evidence classified as VUS.

### Phasing variants by nanopore long-read sequencing

To confirm phasing of candidate compound heterozygous variants, PCR products from long-range PCR were sequenced by Nanopore long-read sequencing. Sequencing library was prepared using the ligation sequencing kit (Oxford Nanopore Technologies LSK-109) with barcoding (Oxford Nanopore Technologies NBD-114) with 100 fmol of PCR amplicon. Libraries were run on a MinION Mk1C using the FLO-FLG001 flow cell with pore version R9.4.1. Reads were processed in real-time with MinKNOW v20.10.3 running Guppy v4.2.2 high accuracy basecaller. Reads in fastq format were mapped to the GRCh38 build of the human genome (version GCA_000001405.15_GRCh38_no_alt_analysis_set available from: ftp://ftp.ncbi.nlm.nih.gov/genomes/all/GCA/000/001/405/GCA_000001405.15_GRCh38/seqs_for_alignment_pipelines.ucsc_ids/GCA_000001405.15_GRCh38_no_alt_analysis_set.fna.gz) with minimap2 (v2.1) using default settings for Oxford Nanopore long reads. The resulting BAM file was sorted with samtools sort v.1.9 and the aligned reads viewed in IGV.

### Validation of copy number variants by DNA microarray

DNA microarray was performed on an Illumina Infinium CytoSNP-850K v1.2 BeadChip. Library preparation, hybridisation, scanning and data acquisition were performed according to the manufacturer’s protocols, as a service by the Australian Genome Research Facility (Melbourne, Australia). Detection of CNV calls was carried out with PennCNV v1.05 using default parameters for autosomal and X-linked analyses respectively^64^ and independently with the cnvPartition 3.2.0 plugin for GenomeStudio 2.0 Software (Illumina) with default parameters. CNV calls were visualised using GenomeViewer in GenomeStudio 2.0. P/LP copy number variants validated by SNP array are shown in Supplementary Figs. 3–5 and Supplementary Fig. 7.

### Validation of copy number variants by quantitative PCR (qPCR)

A total of 30 ng genomic DNA was used per reaction with Power SYBR^TM^ Green PCR Master Mix (Applied Biosystems) on a Stepone Plus real time system (Applied Biosystems) according to manufacturer’s recommended cycling conditions. To determine PCR efficiency, a fivefold dilution series of a single genomic DNA sample from 200 to 12.5 ng per reaction was used to generate a standard curve for each primer pair. All reactions were run in quadruplicate. Amplification was linear across the dilution series, with minimum amplification efficiency achieved being 94%. Copy number of target sequence was determined by calculating relative quantity (RQ) of each target using the equation 2^−ΔΔCT^ as described in^65^. ∆Ct was calculated for each sample relative to reference locus Ribonuclease P RNA Component H1 (RPPH1) (Forward: 5′-TACCTCACCTCAGCCATTGAAC-3′; Reverse: 5′-GTCAGACTGGGCAGGAGATG-3′), with ∆∆Ct determined by normalisation to a calibrator sample. Final copy number for each target is given by 2 × RQ. Primer sequences for qPCR are listed in Supplementary Table 9 and copy number variants validated by qPCR are shown in Supplementary Figs. 2 and 8.

### Validation of copy number variants by MLPA

MLPA probes were designed following manufacturer guidelines (MRC-Holland, Netherlands, www.mrcholland.com, CSM.TECH-001, v04) and experiments performed as described in MLPA^®^ General Protocol MDP v-007. Total probe length in each assay ranged from 96 to 124 nt, with probe lengths differing by a minimum of 3 nt. Each half probe consists of a target specific hybridising sequence and a universal primer specific sequence and was 5′ phosphorylated to facilitate ligation. Phage M13 genome position 3-99 (AC# V00604) was used as a stuffer sequence to achieve the target total probe length^66^. Capillary electrophoresis was performed on an Applied Biosystems 3730 using the LIZ 500 size standard, which can be detected using the GS500-200 channel. Peaks were visualised by generating a manual sheet set in Coffalyser digitalMLPA 201211.1315 (downloadable from www.mrcholland.com). To calculate copy number, intra-sample normalisation was performed by normalising target probe peak height to reference (control) probe peak height in each sample. The intra-sample ratio of target probe in test samples was then normalised to the median intra-sample ratio of target probe in reference samples (five total). Probe sequences for MLPA are listed in Supplementary Table 10 and copy number variants validated by MLPA are shown in Supplementary Fig. 8.

### MethylCapture sequencing

MethylCapture sequencing was performed as a service by Novogene (Hong Kong). Target capture, bisulphite treatment and PCR indexing were performed with the SureSelectXT Human Methyl-Seq Target Enrichment System (Agilent) using 3 ug genomic DNA, as per manufacturer’s directions. Multiplexed libraries were sequenced on a HiSeq2500 (paired end 150 bp reads), with minimum 15 GB raw data generated per sample. Following sequencing, reads were trimmed with TrimGalore v0.4.5 before mapping with bwameth v2.0.1. Picard Tools v2.18.5 was used to merge reads and mark duplicates and MethylDackel v0.2.0 was used to extract strand bias measurements, call methylation status and calculate proportion of methylated/unmethylated at each site. Mapping quality cut-off was set at 30, with minimum read depth 10×, ignoring marked duplicates and accounting for strand biases identified in the previous step.

### X chromosome inactivation analysis

X Chromosome inactivation was tested by DNA digestion with the methylation sensitive restriction enzyme *Hpa*II, and PCR amplification of digested and undigested samples at the highly polymorphic AR locus. Briefly, 1 µg genomic DNA was digested with 25 U *Hpa*II for 16 h at 37 °C before enzyme inactivation (20 min at 80 °C). Mock digestions were simultaneously performed in buffer without enzyme. Digested and undigested DNA samples were purified (QIAquick spin columns, Qiagen, Valencia, CA) and 20 ng of purified DNA was used in PCR with AR primers, with the reverse primer being FAM fluorescently labelled (Forward 5′-TCCAGAATCTGTTCCAGAGCGTGC-3′ and Reverse 5′-GCTGTGAAGGTTGCTGTTCCTCAT-3′). Fragments from both digested and undigested samples were sized and quantified using an ABI 3730, with a 50 cm array and POP-7 polymer. Fragment Analysis was done using the LIZ500 size standard.

### Statistical analysis of comorbidities and environmental risk factors

Statistical analyses were performed in R (version 4.0.3). For each analysis, we used presence of either a P/LP variant or candidate variant in intolerant gene as the dependent variable. Association of individual comorbidities (autism, epilepsy, developmental delay) was tested by the chi-square test. Number of comorbidities in each individual was coded as 0 for no comorbidity, up to 3 for all of autism, developmental delay and epilepsy, and logistic regression performed to test for an association between number of comorbidities and genetic result. Multivariable logistic regression was performed for risk factors sex, gestational age and birth weight centile, to test for an association between any individual risk factor or combination of risk factors with genetic result.

### Reporting summary

Further information on research design is available in the Nature Research Reporting Summary linked to this article.

## Supplementary information

Supplementary Information

Reporting Summary

## Data Availability

Clinically relevant variants reported in this study have been submitted to ClinVar (SCV001737571-SCV001737616). In accordance with informed consent signed by participants in the Adelaide Cerebral Palsy Biobank, sharing of supporting data (including whole genome sequencing, Nanopore long-read sequencing, DNA microarray, and MethylCapture sequencing) is subject to ethical review by the Adelaide Women’s and Children’s Health Network (WCHN) Human Research Ethics Committee. Requests for data can be made by contacting the corresponding author (J.G.).

## Code availability

No custom code was used for data collection or analysis. The following commercial or open source software were used in analysis: bcl2fastq v2.16.0, BWA-MEM v0.7.10-r789, Novosort v1.03.01, Samtools v1.1, GATK tools v3.3, ClinSV 0.9, R v4.0.3, MinKNOW v20.10.3, Guppy v4.2.2, minimap2 v2.1, Samtools sort v.1.9, IGV v2.7.2, PennCNV v1.05, cnvPartition 3.2.0 (Illumina), GenomeStudio 2.0 Software (Illumina), GraphPad Prism 9, ANNOVAR (2019Mar23), DNASTAR Lasergene 10, Seqman Pro8, TrimGalore v0.4.5, bwa-meth v2.0.1, Picard Tools v2.18.5, MethylDackel v0.2.0, Coffalyser digitalMLPA v.140721.1958.
