# Supplementary material for: Yield of clinically reportable genetic variants in unselected cerebral palsy by whole genome sequencing
Source: NPJ Genom Med. 2021 Sep 16;6:74. doi: 10.1038/s41525-021-00238-0 (PMC8445947; doi:10.1038/s41525-021-00238-0)
Supplement: Supplementary file 1 — Supplementary Information [file 41525_2021_238_MOESM1_ESM.pdf]

Supplementary Information

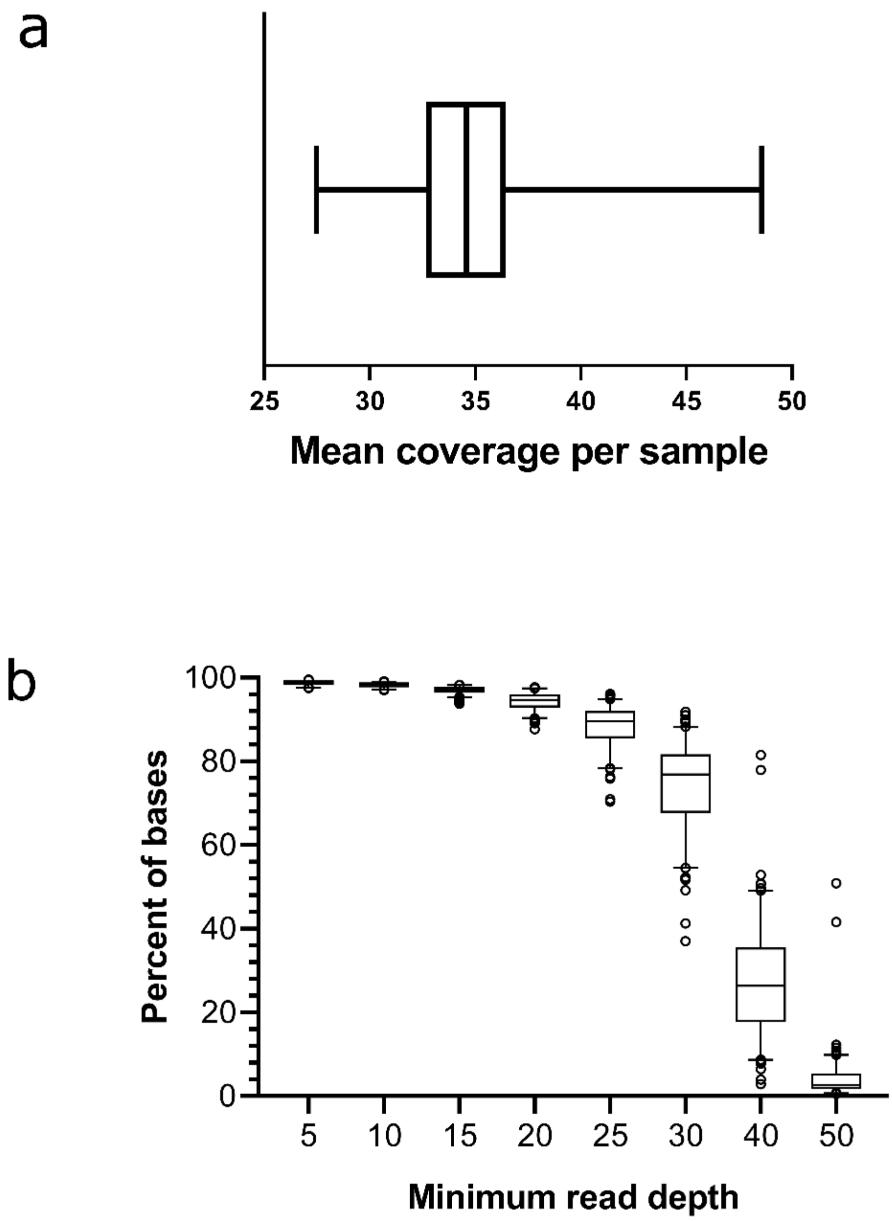

Supplementary Figure 1: Coverage achieved for whole genome sequencing: a) Mean coverage for all samples. Centre line is mean, box limit is 25th-75th percentile, error bars show range minimum to maximum. **b)** Percentage of bases covered at minimum read depths. Error bars are 5th-95th percentiles, centre line is median, box limit is 25th-75th percentile.

Supplementary Table 1: Clinical summaries for 150 individuals sequenced in this study.

Case ID	Clinical summary
**P162**	Female. Born 40/40. Left-sided hemiplegia; spasticity and dystonia.
**P163**	Male. Born 40/40. Hemiplegia; severe spasticity and dystonia of right arm. Hypertonia on elbow flexors, some with pronator. Ulnar deviation of the wrist. Global developmental delay; asthma. MRI - PVL just adjacent to the left lateral ventricle.
**P165**	Male. Spastic hemiplegia. Speech and language delay. Brother has auditory processing disorder and ADHD type symptoms. MRI - Area of abnormality in the left periventricular region with appearances of gliosis to the left ventricle, with otherwise normal findings. Homozygous for *MTHFR* variant with normal homocysteine level, borderline low protein C level, likely lupus anticoagulant present.
**P169**	Male. Born 25/40. Left-sided hemiplegia. Intracranial bleeding; secondary hydrocephalus; VP shunt; pulmonary haemorrhage; jaundice. Epilepsy - abnormal EEG - episodic altered conscious state with prominent autonomic features; esotropia - bilateral eyes (alternating); GORD; asthma.
**P176**	Male. Born 24/40. IVH grade IV resulting in left hemiplegia; porencephalic cyst. Left hand with increased tone; left ankle/foot orthotic; independent walker. Seizures; autism (mild); speech limited; global developmental delay.
**P178**	Male. Born 30/40 following normal pregnancy. Spastic diplegia, mild tremor in hands. Inguinal hernia as a baby. Moderate-severe sensorineural hearing loss; possible auditory processing disorder, otherwise developmentally normal; anxiety.
**P180**	Male. Born 32/40; IUGR; jaundice at birth. Mother had severe pre-eclampsia. Spastic quadriplegia. Sagittal craniosynostosis, (cousin and father also); microcephaly. Afebrile seizures from 7 months, epilepsy diagnosed; GORD; upper airways obstruction necessitating adenoidectomy at 1.5 years; global developmental delay; strabismus; poor weight gain; sleep myoclonus; lethargy.
**P182**	Female. Spastic diplegia with progressive dystonia and ataxia. Initial MRI - abnormality in both putamina, has slightly increased lactate. Diagnosed with Leigh's encephalopathy at 2 years of age.
**P183**	Female. Born 40/40 at home. Spastic hemiplegia. Right hamstring lengthening and transfer and right calf recession. CT - periventricular punctate calcification surrounding the left frontal horn of the left lateral ventricle.
**P185**	Female. Born 42/40 following induction. IUGR. Spastic hemiplegia. Hypothermia after birth. MRI - area of encephalomalacia involving the cortex and adjacent cortical white matter of the posterior frontal lobe in the precentral area. Recurrent bronchiolitis then chest infections from 4 mths old; diagnosed as pseudo-hypoaldosteronism without genetic cause identified; complex partial epilepsy with absence seizures and drop attacks began at 15 months; protein C deficiency; GORD.
**P187**	Male. Born 27/40. Spastic diplegia. Dev. Delay. MRI - Abnormally high signal in the white matter abutting the lateral ventricles bilaterally most marked in the occipital regions with minor lateral wall irregularity and dilatation. Underwent bilateral femoral osteotomies as well as soft tissue procedures.
**P188**	Female. Born 37/40, induced due to oligohydramnios and IUGR. Spastic quadriplegia with some evidence of dyskinesia. Hypoglycaemic day 2 and lactic acidosis on initial cord bloods; apnoeic episodes/seizures day 9; stiff abnormal flexion/extension and fine tremor; difficulties maintaining midline orientation. No evidence of encephalopathy until onset of apnoea and seizures. Day 9 MRI - extensive injury of fronto-parietal white matter basal ganglia and thalamic nuclei; symmetric hypoxic brain injury. Moderate hearing deficits; squint and cortical visual impairment; cranial nerve palsies; asthma; GORD, vomiting and slow weight gain, feeding difficulties; lactose intolerant; recurrent otitis media and tonsillitis necessitating tonsillectomy, adenoidectomy and grommets.

Case ID	Clinical summary
**P189**	Female. Born 30/40. IUGR. Unremarkable perinatal history. Evolving spastic diplegia with dystonia which has decreased with maturity. MRI - extensive bilateral PVL white matter signal changes involving posterofrontal, parietal and occipital regions, without apparent volume loss. Mother has medically managed Graves' disease.
**P191**	Female. Born 41/40. Spastic/dystonic diplegia. Neonatal seizures, resolved but later developed epilepsy. HIE grade II, born with cord around neck. Unexpected flat baby on delivery. Astigmatism. Sibling 1 with ADHD (inattentive type), ASD, astigmatism. Sibling 2 with astigmatism. Both parents with visual problems.
**P192**	Male. Born 29/40. Mild spastic diplegia. IVH on day 1 of life, grade 1. Respiratory distress; neopuff support. Changes of white matter injury of prematurity/PVL. No restricted diffusion to suggest an acute ischaemic focus. Squint (esotropia) requiring two operations and visual impairment. Fully independent; wears hinged AFOs. Developing some foot deformities as he is growing.
**P193**	Male. Born 27/40. Spastic diplegia. No evidence of PVL on MRI. Low tone and hypermobility with normal power and reflexes in upper limbs and spasticity with brisk lower limb reflexes and contractures at ankles; bilateral pes cavus with hammertoes. Past history of chronic lung disease and now asthma; increased iron levels due to congenital liver anomaly - portacaval shunt; poor growth and delayed puberty; bilateral inguinal hernias; left testicular torsion and orchidectomy; atrioseptal defect; bilateral ptosis with elongated facies and high-arched palate. Mother visual problems.
**P199**	Female. Born 30/40. Maternal pre-eclampsia; IUGR; respiratory distress syndrome of newborn. Spastic diplegia. Developmental delay. Sleep apnoea - adenotonsillectomy at 6 years. Bilateral hamstring lengthening and calf recession. Sleep disordered breathing; eczema; family history of Haemophilia A in father. Four cranial U/S at birth, all normal.
**P201**	Male. Born 39/40. IUGR. Spastic quadriplegia. CT - Extensive encephalomalacia primarily involving the left parietal and frontal lobes and also to a lesser extent the superior aspect of the right parietal lobe. Developing calcification throughout the cerebrum. Required bag and mask ventilation for 2mins when born. Prolonged seizures involving facial twitching and left arm and shoulder jerking at 1 month - abnormal EEG showed burst suppression pattern. Family history of epilepsy (paternal grandfather and uncle); failure to thrive; irritability; pyridoxine deficiency; GORD; severe intellectual disability; gastrostomy fed; severe scoliosis; eczema.
**P205**	Male. Born 29/40. Spastic diplegia. MRI - Periventricular deep white matter gliosis and volume loss consistent with PVL. Reduced fetal movement; admitted to NICU immediately after birth with fetal distress syndrome - given magnesium sulphate, ventilation and surfactant doses for 6 days; echogenic bowel was detected on morphology scan; prematurity associated jaundice; apnea of prematurity and anaemia; retinopathy of prematurity; MRSA positive; cholestasis. Congenital heart disease - multiple ventricular septal defect; cystic fibrosis (classical) - bowel obstruction secondary to meconium ileus managed by resection of some gut and jejunostomy; secondary intestinal obstruction and adhesions repaired by laparatomy and appendicectomy; chronic lung disease from mechanical ventilation (finally extubated at 50 days of age); pancreatic insufficiency; gastrostomy; conjugated hyperbilirubinaemia secondary to CF liver disease.
**P211**	Female. Born 40/40. IUGR. Jaundice. Spastic/dystonic hemiplegia, with severe spasticity on left side. MRI - focal dysplasia in right frontal lobe. Irregular cortex in the right frontal region, with the appearance of a dysplasia.
**P214**	Male. Born 40/40. Spastic/dyskinetic quadriplegia. IUGR; poor CTG and decreased fetal movements resulting in CS. Resuscitation: flat at delivery - required IPPV, ECM (1min), CPAP, oxygen. Taken to NICU, intubated approx. 3 days. Day 1 became apnoeic, day 2 seizures, stiffened limbs and trunk, myoclonic jerking of limbs; hydrocephalus on CT scan; microcephaly; ongoing status epilepticus. Global developmental delay; epilepsy; cortical visual impairment; poor weight gain.

Case ID	Clinical summary
**P217**	Male. Born 41/40. Spastic diplegia. MRI -Brain structurally normal initially, with subtle delay of myelination in temporal lobes and incidental cerebellar ectopia later noted. Kinking of optic nerves. Severe spasticity affecting lower limbs. Neurologist suspected HSP at 10 years of age on the basis of his gait pattern, normal MRI and static presentation. Gross motor delay, otherwise development is normal; tendo-Achilles tightening; iron deficiency anaemia and microcytosis.
**P220**	Male. Born 37/40 by CS after fetal bradycardia and maternal pyrexia. Spastic hemiplegia. Presented at day 4 with right myoclonic seizure, left MCA infarct with residual right hemiparesis. Epilepsy; developmental delay.
**P225**	Female. Born 29/40. IUGR; augmented labour due to chorioamnionitis and funisitis. Spastic/dyskinetic quadriplegia. MRI - Bilateral PVL, more marked on the left within the posterior frontal and parietal white matter. US: Small grade 1 germinal matrix haemorrhage. Dev. delay; heart murmur. Sister 1 ASD + ADHD, and Sister 2 ASD + OCD).
**P226**	Male. Born 26/40. Spastic hemiplegia. IVF pregnancy, twin 2 with IUGR, GBS positive pregnancy, Grade 4 IVH with limited extension into periventricular area and only small area of leukomalacia.
**P227**	Female. Born 28/40, IVF pregnancy, triplet 2/3. One triplet IUD at 23/40. Spastic diplegia. MRI - mild generalised atrophy, increased calibre of the ventricles and extra-axial spaces. Changes of cystic encephalomalacia and volume over the parietal convexities, bilaterally.
**P228**	Male. Born 24/40. Spastic diplegia. Visual problems. Hypoplastic thumbs (sister also). Two sisters, both with low tone. Eosinophilic oesophagitis; laryngomalacia; Chronic lung disease; Patent ductus arteriosus; bilateral intracranial haemorrhage (grade 1). Maternal ulcerative colitis, born with 1 kidney.
**P229**	Female. Born 41/40. Spastic/athetoid quadriplegia. Dev. delay. MRI - Bilateral hyper intense signal within both thalamic nuclei.
**P230**	Male. Born 39/40. Spastic hemiplegia. MRI - Unilateral encephalomalacia within the deep white matter of the left frontal lobe extending into the anterior aspect of the left parietal lobe. Evidence of Wallerian degeneration involving the cortical spinal tracts.
**P231**	Female. Born 37/40. Spastic quadriplegia. Birth complicated by shoulder dystocia. Maternal GBS positive with antibiotics. Glutaric Acidemia type 2, with no metabolic condition found. Dev. delay; Epilepsy; Cortical visual impairment; impetigo.
**P232**	Male. Born 38/40. Spastic diplegia. Maternal pre-eclampsia and heart condition (valve problem) during pregnancy. Truncal hypotonia and increased spastic tone in leg; microcephaly; poor head control. Global developmental delay; onset of seizures at 6 years of age; CT - possible cerebral dysplasia and lissencephaly. PVL.
**P233**	Female. Born 38/40. Severe spastic and dystonic left hemiplegia. Neglect of left side. Strawberry nevus. Mild thoracic scoliosis and inverted left foot. MRI – thin body of corpus callosum, secondary to PVL on right side with associated exvacuo dilatation of the right lateral ventricle. Centrum semiovale gliosis / fronto-parietal white matter loss. Small area of blooming artefact at the right periventricular region, with resultant porencephalic cyst, which communicates with the lateral ventricle.
**P235**	Male. Born 40/40. Spastic quadriplegia. Increasing seizure frequency, global dev delay, frontoparietal polymicrogyria, microcephaly, cortical visual impairment. Bilateral adductor releases at 2 yrs of age. Strong family history of developmental dysplasia of the hip. MRI - Extensive and diffuse bilateral symmetrical cortical dysplasia involving the majority of both hemispheres but sparing the posterior fossa. Delayed myelination.
**P236**	Male. Born 38/40. Spastic diplegia with some dystonia, spasticity possibly progressive. Speech and developmental delay; myopia. MRI - delayed myelination; spinal cord abnormalities. Maternal type 1 diabetes.

Case ID	Clinical summary
**P701**	Male. Born 40/40. Spastic/dyskinetic diplegia. Uterine rupture, diagnosed neonatal encephalopathy. Non-verbal, understands signs and face expressions; mild episodic asthma; partial epileptic seizures at 3 years of age needs phenobarbitone. US - Ventricles and extra axial spaces are decreased in size. No intracranial haemorrhage demonstrated.
**P703**	Male. Dystonic/spastic quadriplegia. Cord prolapse; ill at delivery requiring 4 hrs of hand ventilation and had 2 pneumothoraxes. Neonatal seizures. Significant chronic and persistent pain relating to spasms in lower back and hips. Gait deteriorated over years. US 2 days - Increase of parenchymal echogenicity of both cerebral hemispheres, associated with thickening of sulci and moderate hyper-echogenicity of the thalami. US 6 mths: Prominent ventricular system and sulcal pattern is seen. Finding is consistent with non-specific widening of CSF containing spaces.
**P704**	Male. Born 40/40. IUGR. Spastic/dyskinetic quadriplegia. Hypoglycaemia, polycythemia and infection in perinatal period requiring blood transfusion. Tetralogy of Fallot (sister also). MRI - periventricular leukomalacia and left basal ganglia calcification.
**P706**	Male. Born 31/40. Spastic/dyskinetic quadriplegia. Epilepsy and global dev. delay. CT - Asymmetry between lateral ventricles, with both lateral ventricles slightly prominent but third and fourth ventricles normal size and extra-axial spaces not enlarged. Prominent cisterna magna but the posterior fossa structures appear normal in size.
**P708**	Male. Born 40/40. IUGR. Dystonic hemiplegia with some spasticity. Mobility decreased and increasing dystonia. Dev. delay; bleeding or clotting disorder; hearing loss.
**P710**	Female. Born 32/40. Spastic diplegia. Kidney scarring. Significant planovalgus deformity at the left ankle. MRI - Periventricular white matter loss and hyperintensity.
**P711**	Male. Born 40/40. IUGR. Spastic hemiplegia.
**P712**	Male. Born 27/40. Spastic diplegia. Neurofibromatosis type 1; hearing difficulties; slightly small cochlear nerve on left side; metopic craniosynostosis with normal appearance of brain.
**P714**	Male. Born at >37/40. Spastic/dystonic quadriplegia. Erb's palsy; developmental delay. Diagnosed with HIE at term, possible acute on chronic. MRI - cortical and subcortical frontoparietal and occipital diffusion restriction extending into parasagittal white matter, centrum semiovale, posterior limb of the internal capsules bilaterally, anterior thalamus, post central gyrus on the right left posterior and inferior cerebellum. MRI at 2 years - atrophy and high signal in both parasagittal and occipital regions, as well as both motor cortex regions, more marked on the left. Mild atrophy and heterogeneity of the thalamus bilaterally. Wedge-shaped atrophy of the left cerebellar hemisphere, with subtle changes on the right. Overall asymmetry of the hemispheres with the left being smaller.
**P715**	Male. Born 42/40. Spastic/dystonic hemiplegia. Neonatal seizures, CVA at 3 weeks of age. Mild intellectual impairment, seizure disorder, behavioural and emotional issues (aggressive behaviour and progressive outbursts), complex congenital heart disease.
**P719**	Female. Born 36/40. IUGR. Spastic hemiplegia - dense right hemiparesis with dysarthria; severe drooling; Polycythaemia; Epilepsy; Developmental disability and right visual field defect. MRI - Large area of cystic encephalomalacia in the left MCA territory with significant attenuation of the left MCA and its branches. Small area of cystic encephalomalacia in the right MCA territory, severe PVL with evidence of middle cerebral artery occlusion on the left side.
**P720**	Male. Born 40/40. Spastic hemiplegia with some dystonia in left arm. Intermittent right knee pain and headaches. Asthma. Older brother ASD. MRI - Periventricular white matter loss and areas of abnormal signal in the periventricular white matter, consistent with periventricular leukomalacia (PVL). Asymmetry in the size of the thalami.

Case ID	Clinical summary
**P721**	Female. Spastic/dystonic quadriplegia; has had bilateral hip releases and hamstring releases. Significant planovalgus deformity, worsening. Dysphonia; breathing difficulty.
**P730**	Male. Born 28/40. IUGR. Spastic/dystonic quadriplegia. Significant scoliosis; dev. delay, intellectual disability; severe vision impairment; epilepsy; osteoporosis; gastro-oesophageal reflux. Maternal Protein C resistance. CT - Prominent ventricular system with irregular margins of the lateral ventricles. White matter atrophy.
**P736**	Female. Born 27/40. Asymmetric spastic diplegia. Left calcaneal shift and lateral column lengthening. Dev. delay; OCD (Mother also).
**P737**	Male. Born 26/40. Spastic/dystonic quadriplegia. Right plagiocephaly. CT - Right lambdoid suture is narrower than the left with some heaped-up bone over the inner table. Sclerosis and a relatively featureless right lambdoid suture may suggest early craniosynostosis.
**P739**	Male. Born 36/40. IUGR. Spastic/dystonic quadriplegia with relative sparing of right arm. Has developed severe pain in right arm (localised around elbow), otherwise stable. Visual problems. MRI – Bilateral, nearly symmetrical periventricular white matter hyperintensity, predominantly involving the deep white matter is suggestive of a demyelination/ dysmyelination disorder.
**P740**	Male. Born 28/40. Spastic quadriplegia, lower limbs more severely affected.
**P741**	Female. Born 40/40. Spastic quadriplegia, unable to bear weight and spends most of day in wheelchair; severe contracture of right wrist. Congenital CMV infection; chronic staphylococcus infection requiring 12 months antibiotics; Ep, visual problems, scoliosis, osteoporosis, dev. delay, deaf - 3/4 siblings in family deaf, 1 sibling with ASD and OCD. MRI - brain is diffusely abnormal. Cerebral appearances consistent with intrauterine CMV infection. Inner ear structures appear normal. CT - enlarged lateral ventricles, with no evidence of acute hydrocephalus.
**P743**	Female. Born 26/40, twin 2/2. Severe spastic/dyskinetic quadriplegia. Dev. delay, hearing loss, chronic lung condition. Severe spasm and progressive scoliosis with underlying osteoporosis causing distress and pain. MRI - Generalised bilateral cerebral atrophy, with mild progression.
**P745**	Male. Spastic hemiplegia.
**P746**	Female. Spastic diplegia. Good functional use of upper limbs; evidence of spasticity at hamstring bilaterally, with knee flexion contractures. Developing crouched gait with planovalgus foot position. Difficulty with distant vision.
**P747**	Female. Born 29/40. Spastic diplegia. Dev. delay, ASD, visual problems. Stiff leg with marked ankle pronation. Aggressive behaviour. MRI -bilateral periventricular leukomalacia.
**P748**	Male. Born 40/40. Spastic hemiplegia. Stutter; eczema. MRI - Encephalomalacia within the left corona radiata/body of the caudate. No other focal abnormality and MRA is normal.
**P749**	Female. Born 31/40. IUGR. Predominantly dystonic quadriplegia. Progressive and profound hearing and visual problems. Ongoing problem with hip pain. Anxiety issues; drooling; dryness in mouth. MRI - Widespread peribronchial white matter loss and gliotic change. Slight increase in signal in the vestibular cochlear nuclei. Right cochlear nerve a little smaller than left.
**P750**	Male. Born 42/40. Bilateral dystonia. Bilateral hip subluxation. Iron deficiency anaemia (resolved); pulmonary stenosis (balloon dilation, aged 3 months).
**P751**	Female. Born 26/40. Spastic/partially dystonic hemiplegia. Dev. delay. MRI - Gliotic changes in the left cerebellar hemisphere. Stable appearance of the right cerebral, left cerebellar and cerebellar vermis volume loss. Severe bilateral intraventricular and intracranial haemorrhages.

Case ID	Clinical summary
**P752**	Male. Spastic hemiplegia.
**P753**	Male. Born term. Spastic/dystonic quadriplegia. Bilateral hip reconstruction. Obstructed labour. Born by LSCS, seizures from 6 hours of age, resistant and required multiple anticonvulsants. MRI – Bilateral periventricular cystic changes in the centum semi ovale. Extensive cystic encephalomalacia. US - Absent corpus callosum and cystic space in the right parietal region.
**P754**	Male. Born 40/40. Spastic quadriplegia. Global developmental delay; autistic behaviour; some visual impairment; difficult behavioural management. MRI - partial agenesis of the corpus callosum.
**P756**	Female. Born 40/40. Spastic hemiplegia. MRI - Small calibre M1 segment of left MCA with paucity of distal branches.
**P758**	Male. Born 40/40. Spastic diplegia. Autism spectrum disorder, hearing loss. MRI - open lipped schizencephaly on the right and either transmantle heterotopia or closed lip schizencephaly on the left. These are associated with large extra-axial CSF spaces. Suggestion of some mass effect on the left side with enlargement of the middle cranial fossa. Multiple areas of polymicrogyria. Absence of the septum pellucidum and small optic nerves.
**P759**	Male. Born 37/40. Spastic hemiplegia.
**P760**	Female. Born 40/40. Spastic hemiplegia. Father ADHD.
**P761**	Female. Born 30/40. Spastic diplegia, both knees flexed during gait. Dev. delay; visual problems.
**P763**	Female. Born 37/40. IUGR; emergency CS after low amniotic fluid noted. Spastic hemiplegia. Dev. delay, visual problems, epilepsy, ASD. Mother epilepsy; brother with autism, dev. delay. MRI - Ex-vacuo dilation of the left lateral ventricle associated with periventricular white matter volume loss and signal abnormality as well as reduced volume of the left basal ganglia and left side of the brain stem. Non-specific white matter signal abnormality in the right frontal region, but right cerebral hemisphere otherwise normal appearance.
**P769**	Male. Born 25/40. IUGR; identical twin died at 1 day old. Spastic diplegia. Global developmental delay; ASD; sleep disturbances; osteopenia; bilateral inguinal hernias; conjugated hyperbilirubinemia. Brother with ADHD.
**P772**	Male. Born 37/40. Dystonic dyskinetic quadriplegic CP - very dystonic with lots of involuntary dsykinetic movements. Arthrogryposis affecting leg (Father also). Dev. delay.
**P773**	Male. Born 28/40. Spastic diplegia. ADHD.
**P775**	Male. Born 38/40. Parents second cousins. IUGR. Spastic dystonic quadriplegia. Seizures as neonate; dev. delay; bilateral sensorineural hearing deficiency; visual problems; bilateral dislocated hips, left worse than right. Fixed knee flexion contracture requiring soft tissue release. Developing right wrist flexion deformity/contractures and also has dysphagia. MRI - Cystic lesions in basal ganglia and thalami with diffuse cerebral volume loss. The internal auditory canals appear bulbous bilaterally, but contain normal nerves.
**P776**	Male. Born 36/40. IUGR. Asymmetric spastic diplegia. ASD, dev. delay, intellectual disability and seizure disorder with consequent behaviour disturbances and aggressive behaviour; ADHD; OCD; anxiety/depression. Pregnancy complicated by bleeding at 6 months and then intermittent bleeding from 8 months until delivery. MRI - Bilateral periventricular white matter volume loss with signal changes, consistent with periventricular leukomalacia. Mild cerebral cerebellar degenerative changes noted.
**P778**	Female. Born 39/40. Spastic hemiplegia. Right vulpius calf release, flexor hallucis longus/flexor digitorum longus release.
**P779**	Female. Born 28/40. IUGR; dichorionic diamniotic twin pregnancy complicated by gestational hypertension, antepartum haemorrhage, placental abruption, foetal bradycardia and in utero death of a male twin. Mother 3 previous still births, 3 live births. Spastic diplegia. Severe periventricular

Case ID	Clinical summary
	haemorrhage in newborn period (grade 3 haemorrhage on left side and grade 4 haemorrhage on right side). Post-haemorrhagic hydrocephalus. Dev. delay - communication skills and gross motor function skills. Hearing loss (sister also).
**P780**	Male. Born 36/40. Spastic hemiplegia. Congenital motor nystagmus; epilepsy; neonatal jaundice. Sister OCD.
**P782**	Male. Born 36/40. IUGR. Spastic/dystonic hemiplegia. Mild to moderate developmental delay; ADHD.
**P783**	Male. Born 38/40. IUGR. Spastic hemiplegia. Parents are cousins. Moderate developmental disability; seizures; tachycardia; hypoglycaemia; hyperinsulinism. MRI - Right parietofrontal haemorrhage. White matter volume loss with ex vacuo dilatation of the frontal horn of the right lateral ventricle. Prominence of the ventricles and extra axial CSF spaces.
**P784**	Female. Born 38/40. Spastic diplegia. Global developmental delay/disability; ADHD (Mother also); history of femoral fracture; worsening contractures of knees and ankles. MRI - Progression in demyelination. Stable appearance of white matter changes; patchy in frontal regions and more confluent posteriorly.
**P785**	Female. Born 38/40. Spastic hemiplegia. Poor balance; speech delay; swallowing difficulties; vomits easily; muscle development and imbalance.
**P786**	Female. Born 25/40. Dystonic/spastic quadriplegia (GMFCS 5). Necrotising enterocolitis; retinopathy in neonatal period; developmental delay; epilepsy. US (5 weeks) - reduced sulcation, no evidence of IVH.
**P788**	Male. Dystonic quadriplegia with associated dysphagia. Developmental delay (brother and sister also); epilepsy. Mother 13 previous miscarriages. MRI - postnatal herpetic meningoencephalitis.
**P789**	Male. Born 40/40. Spastic hemiplegia. Associated homonymous hemianopia; right sided visual defects; learning difficulties; complex partial seizures. Mother polycystic kidney disease. CT - large old infarct involving the left MCA territory with associated ex vacuo dilatation of the left lateral ventricle. No identifiable hydrocephalus.
**P790**	Male. Born 38/40. IUGR. Pre-eclampsia. Spastic/dystonic quadriplegia. Global developmental delay/disability; cortical visual impairment; hearing impairment; dysphagia; epilepsy.
**P792**	Male. Born 30/40. IUGR. Asymmetric spastic diplegia. Speech delay and global development delay; drooling; complex seizures.
**P795**	Female. Born 25/40. Spastic/dystonic diplegia. Trend for internal rotation at hips with some knee flexion and anterior pelvic tilt bilaterally; quite significant dystonia in calves. Chronic lung disease which required home oxygen; epilepsy; auditory processing disorder and language problems; deafness; visual problems; anxiety disorder.
**P796**	Male. Born 40/40. Spastic/dystonic quadriplegia. Developmental delay; visual impairment; epilepsy; swallowing difficulty; seizures. Sustained a bilateral intraventricular haemorrhage, subsequently developed hydrocephalus which required a ventriculoperitoneal shunt. CT 2 weeks: Resolving left temporoparietal occipital haematoma with extensive perilesional oedema. Acute hydrocephalus with periventricular/ transependymal oedema. Possible vermian hypoplasia. Extent of haematoma unusual for a term infant. An underlying vascular malformation cannot be excluded.
**P798**	Female. Born 39/40. Spastic hemiplegia. Neonatal seizures. Dev. delay; learning difficulties with verbal based skills; difficulties with attention and hyperactivity. Bleeding or clotting disorder. MRI – left cerebral hemisphere oedema secondary to left middle cerebral arterial ischaemic infarcts.
**P799**	Male. Born 38/40. Spastic hemiplegia. Congenital microcephaly; epilepsy; autism; ADHD; OCD; anxiety/depression; developmental delay; various visual problems including hemianopia and optic nerve damage. Increasing weight causes difficulty with left foot position, increasing physical aggressiveness. MRI, 2 months - Right middle cerebral artery infarct with changes of porencephaly. Smaller capsular and basal ganglionic infarcts

Case ID	Clinical summary
	on the left side. Diffuse and generalised cortical volume loss. Thin and hypoplastic right MCA. MRI, 5 years - Gliotic change in the left periventricular region surrounding the encephalomalacic cavity has increased and evidence of wallerian degeneration has developed in the right thalamus. Extensive right middle artery territory cystic encephalomalacia and small right middle cerebral artery changes are stable. MRI, 11 years- Extensive cystic encephalomalacia and gliosis in the right middle cerebral artery territory does not show significant interval change. Wallerian degeneration involving the bilateral thalami and brainstem as described. Bilateral paranasal sinus disease.
**P801**	Female. Born 31/40. IUGR. Spastic triplegia with minimal effect on right leg. Visual problems. Grade 2 intraventricular haemorrhage at birth (VP shunt insertion in the newborn period).
**P802**	Female. Born 40/40. Spastic/dystonic hemiplegia. Visual problems.
**P902**	Female. Born 35/40. IUGR. Spastic hemiplegia. Dev. delay. MRI - regions of gliosis as demonstrated by T2 high signal and parenchymal volume loss are seen in the region of the right thalamus and basal ganglia.
**P904**	Male. Born 28/40. Twin 2/2 (monozygotic), twin 1/2 has no health conditions. Spastic diplegia. ASD; ADHD; anxiety; visual problems. MRI - periventricular white matter hyperintensity and minor dilation of the lateral ventricles.
**P905**	Male. Born 40/40. Dystonic hemiplegia. Neonatal seizures. Brother ADHD and anxiety/depression. MRI brain and spine – no specific pathology.
**P907**	Male. Twin 2/2 (dizygotic), born 28/40. IUGR. Spastic diplegia. Dev. delay. MRI – periventricular leukomalacia.
**P910**	Male. Born 40/40. Spastic/dystonic quadriplegia. Congenital microcephaly; infantile spasms; epilepsy; developmental delay; visual problems. MRI - bilateral polymicrogyria.
**P911**	Female. Born 40/40. Spastic hemiplegia. ADHD (Father also); visual problems (Mother also). MRI – periventricular leukomalacia.
**P913**	Male. Born 41/40. IUGR. Spastic diplegia. Speech delay; ASD (maternal uncle also). Father ADHD. MRI – no specific brain or spinal abnormality. Past osteomyelitis.
**P918**	Male. Born 35/40. IUGR. Spastic quadriplegia. Intellectual disability (sister also); epilepsy (sister and mother also); OCD (sister also); severe receptive and expressive language disorder; visual problems (mother also).
**P919**	Female. Born 28/40. Spastic hemiplegia.
**P921**	Male. Born 30/40. Spastic diplegia.
**P926**	Male. Pregnancy with placenta praevia and antepartum haemorrhage. Spastic hemiplegia. Developmental delay. Mother 3 stillbirths, 3 live births.
**P928**	Female. Born 29/40 following antepartum haemorrhage. Spastic diplegia.
**P931**	Male. Born 40/40. IUGR. Spastic/dystonic quadriplegia. Developmental delay. Bleeding or clotting disorder (father also). Parents cousins.
**P934**	Male. Born 41/40. Spastic hemiplegia. MRI Brain - focal area of gliosis involving the posterior limb of the right internal capsule and right centrum semiovale.
**P936**	Male. Born 33/40. IUGR. Spastic diplegia. Periventricular leukomalacia. Dyslexia. Two sisters with ADHD and anxiety/depression.
**P938**	Female. Born 31/40. Asymmetric spastic diplegia. ADHD (sibling also). Two siblings with ASD. MRI - bilateral parietal periventricular white matter abnormal T2 hyperintensity.

Case ID	Clinical summary
**P939**	Female. Born 32/40. IUGR. Spastic hemiplegia. Congenital nystagmus with cortical visual impairment. MRI - T2 hyperintensity in the periventricular white matter around the superolateral margin of the body of the left lateral margins of the trigone on each side. Porencephaly and gliosis.
**P940**	Male. Born 29/40. IUGR. Spastic hemiplegia. MRI – periventricular leukomalacia.
**P943**	Male. Born 27/40. Spastic diplegia. Developmental delay; scoliosis. Maternal uncle ASD. MRI – periventricular leukomalacia.
**P944**	Male. Born 38/40. Spastic hemiplegia. Developmental delay; epilepsy; learning difficulties. MRI - Previous focal cortical infarcts in the right frontal, right parietal, and right occipital lobes.
**P945**	Male. Born 28/40. Spastic/dystonic quadriplegia. Developmental delay; epilepsy; visual problems. MRI - extensive PVL.
**P947**	Male. Born 25/40. Spastic/dystonic quadriplegia. Developmental delay.
**P948**	Female. Born 30/40. Spastic diplegia. MRI - Periventricular T2 hyperintensity.
**P949**	Male. Born 27/40. Spastic diplegia. Ventricular septal defect, closed spontaneously; hydromyelia in spinal cord; visual problems.
**P953**	Female. Born 40/40. Spastic hemiplegia. MRI – small area of gliosis.
**P955**	Female. Spastic diplegia. ID, facial dysmorphism, expressive language disorder with no speech. MRI – periventricular white matter changes.
**P955**	Female. Spastic diplegia. ID, facial dysmorphism, expressive language disorder with no speech. MRI – periventricular white matter changes.
**P957**	Male. Born 39/40. Spastic hemiplegia. Anxiety/depression. MRI – periventricular leukomalacia, changes more marked on right side.
**P959**	Male. Born 39/40. IUGR. Spastic/dyskinetic quadriplegia. Placental infarction. MRI – periventricular leukomalacia.
**P962**	Female. Born 39/40. Spastic hemiplegia. MRI - Periventricular white matter gliosis, more marked on the left.
**P965**	Male. Born 41/40. IUGR. Spastic hemiplegia. MRI - Right schizencephalic cleft, lined by extensive areas of polymicrogyria. Associated periventricular gliosis bilaterally.
**P966**	Female. Born 29/40. Spastic diplegia. Developmental delay.
**P968**	Female. Born 40/40. Spastic/dystonic hemiplegia. Diagnosed neonatal encephalopathy. Learning difficulty; ADHD; OCD.
**P972**	Female. Born 29/40. Spastic quadriplegia. Left arm involuntary movement. Pregnancy complicated by diabetes and hypertension. Patent ductus arteriosus. MRI – evidence of previous IVH and mild PVL.
**P974**	Female. Born 37/40. Spastic hemiplegia. ASD (Uncle also), brother with ADHD. MRI - enlarged right ventricle with periventricular gliosis.
**P980**	Male. Born 26/40. Spastic quadriplegia. Neonatal seizures, developmental delay; epilepsy; OCD. MRI - Grade 4 Germinal matrix haemorrhage with IVH.
**P983**	Male. Born 35/40. Spastic diplegia. Developmental delay; visual problems. MRI - PVL.
**P1105**	Male. Born 31/40. Spastic/dystonic quadriplegia, with spasticity dominant. Developmental delay; eosinophilic oesophagitis; asthma; visual problems.
**P1106**	Female. Born 27/40. IUGR. Spastic diplegia. Developmental delay; chronic lung disease; visual problems.
**P1110**	Female. Born 38/40. Spastic triplegia. Squint. Hydrocephalus and antenatal IVH detected at 37/40. Maternal uncle with ASD, ADHD.

Case ID	Clinical summary
**P1114**	Male. Born 36/40. Spastic quadriplegia. Neonatal seizures; autistic features; shunted hydrocephalus; severe global developmental delay; seizure disorder; visual impairment with roving nystagmus. CT - Longstanding shunted hydrocephalus. Large cystic spaces predominantly in a peritrigonal distribution, in addition to Grade III intracranial haemorrhage, with bilateral intraventricular and subependymal haemorrhages and asymmetric dilation of lateral ventricles.
**P1123**	Male. Born 41/40. IUGR. Spastic/dystonic quadriplegia. Neonatal seizures; hypoxic ischaemic encephalopathy; foetal bradycardia intrapartum. MRI - Foci of gliosis involving periventricular deep white matter both cerebral hemispheres.
**P1124**	Male. Born 27/40. Spastic quadriplegia. Congenital microcephaly; antepartum haemorrhage; neonatal seizures; epilepsy; shunted hydrocephalus, aortic stenosis; developmental delay. Paternal grandmother CP.
**P1129**	Male. Born 37/40. Spastic hemiplegia.
**P1130**	Male. Born 40/40. Spastic hemiplegia. ASD (brother also); MRI - abnormal T2 weighted high signal intensity gliosis associated with ex vacuo dilation of the left lateral ventricle.
**P1132**	Male. Born 39/40. Spastic hemiplegia. Mild language delay. MRI - Right sided periventricular gliosis and ex vacuo dilatation of body and trigone of right lateral ventricle. Haemosiderin noted in right caudothalamic groove.
**P1133**	Male. Born 27/40. Spastic hemiplegia. Exomphalos; Beckwith-Wiedemann Syndrome. US - Evidence of intraparenchymal haemorhage with ventricular dilatation/porencephalic cyst and associated periventricular leukomalacia.
**P1134**	Male. Spastic/dystonic hemiplegia.
**P1136**	Female. Born 40/40. Spastic hemiplegia. Asthma; seizures as neonate; epilepsy; developmental delay; visual problems. MRI – MCA stroke.
**P1137**	Female. Born 29/40. Spastic hemiplegia. Astigmatism; asthma; eczema. Stroke in utero and klebsiella pneumoniae meningitis in neonatal period.
**P1138**	Male. Born 41/40. Spastic hemiplegia. Right-sided porencephalic cyst.
**P1140**	Female. Born 26/40. Spastic hemiplegia. Intellectual impairment; developmental delay; ASD; complex partial seizures; shunted hydrocephalus; anxiety disorder; eczema; hearing loss; heart murmur. MRI - Marked white matter volume loss left parietal and occipital lobes corresponding with site of previous left grade IVH. Ventricles of normal size with VP shunt in situ.
**P1141**	Male. Born 40/40. Spastic quadriplegia. Pre-eclampsia. Resuscitation and ventilation required; neonatal seizures; developmental delay; epilepsy; constipation.
**P1145**	Male. Born 38/40. Spastic hemiplegia. Epilepsy; ASD; global developmental delay. MRI - Large area of right middle cerebral artery territory cystic encephalomalacia with marginal gliosis, associated marked volume loss right cerebral hemisphere with midline shift to the right, mild ex vacuo dilatation right lateral ventricle and secondary Wallerian degeneration involving right thalamus and right lateral aspect of brain stem.
**P1146**	Male. Born 27/40, twin. Spastic quadriplegia. Neonatal seizures, epilepsy, developmental delay. Neonatal ventricular haemorrhage. Shunted hydrocephalus.
**P1147**	Female. Born 24/40, twin. IUGR. Spastic quadriplegia, asymmetric. Epilepsy, seizure onset approx. 9 years. Hydrocephalus with VP shunt.
**P1149**	Male. Born 37/40. Spastic quadriplegia. Scoliosis; dislocated hip; drools failure to thrive; developmental delay; kidney abnormality. Cousin with developmental delay, hole in heart.
**P1150**	Female. Twin. Spastic hemiplegia.

Supplementary Table 2: Summary of prioritised variants validated in this cohort.

Case ID	Gene	Variant/<br>Inheritance	Frequency (gnomAD or MGRB)	CADD Phred	Ratio observed/ expected (gnomAD)
P162	*ARHGAP32*	Chr11:128848764G>T: NM_014715.3:c.934C>A:p.R312S<br>Heterozygous, not maternal, Father deceased	0	34	Missense o/e = 0.85<br>(CI 0.81 - 0.9)
P163	*KAT6A*	Chr8:41790507T>C: NM_006766.3:c.5231A>G:p.Y1744C<br>Heterozygous, not maternal	0	22.3	Missense o/e = 0.83<br>(CI 0.78 - 0.87)
P165	*HTT*	Chr4:3225824G>A:NM_002111.8: c.7731G>A:p.W2577*<br>Heterozygous, inheritance unknown	0	52	pLOF o/e = 0.12<br>(CI 0.08 - 0.18)
	PARK2	Chr6:162859017_163164739dup<br>Inheritance unknown	0.002	.	pLOF o/e = 0.55<br>(CI 0.36 - 0.86)
	TRIM32, ASTN2	Chr9:119311659_119462832del<br>Heterozygous, inheritance unknown	0	.	ASTN2 pLOF o/e = 0.14<br>(CI 0.08 - 0.25)
P169	*GRIN2B*	Chr12:13764700A>T:NM_000834.5: c.1739T>A:p.F580Y<br>Heterozygous, inheritance unknown	0	29.8	Missense o/e = 0.48<br>(CI 0.44 - 0.52)
P176	*ARHGAP31*	Chr3:119128396del:NM_020754.2:c.1699del:P567Rfs*28<br>Heterozygous, maternal	0	.	pLOF o/e = 0.09<br>(CI 0.05 - 0.19)
P178	*MFN2*	Chr1:12071568G>A:NM_001127660.2: c.2220G>A:p.W740*<br>Heterozygous, paternal	0	54	pLOF o/e = 0.13<br>(CI 0.07 - 0.28)
	CAMTA1	Chr1:7724952C>T:NM_001349608.2:c.2255C>T:p.S752F<br>Heterozygous, maternal	0	28.7	Missense o/e = 0.71<br>(CI 0.67 - 0.76)
	PARK2	Chr6:162717356_162916007dup<br>Inheritance unknown	0.006	.	pLOF o/e = 0.55<br>(CI 0.36 - 0.86)
P180	*SKI*	Chr1:2237568A>T:NM_003036.4:c.1877A>T:p.K626M<br>Heterozygous, inheritance unknown	7.25E-05	22.5	Missense o/e = 0.8<br>(CI 0.73 - 0.87)
	SLC2A1	Chr1:43395364TT>AC:NM_006516.3:c.767AA>TG:p.K256V<br>Heterozygous, inheritance unknown	1.42E-05	24	Missense o/e = 0.53<br>(CI 0.46 - 0.6)
	SCN1A	Chr2:166896000C>T:NM_001165963.4:c.2522C>T:p.T841M<br>Heterozygous, inheritance unknown	3.19E-05	25.3	Missense o/e = 0.55<br>(CI 0.51 - 0.59)
P182	*GALC*	Chr14:88411975C>T:NM_001201401.2:c.1523G>A:p.R508H<br>Heterozygous, paternal	3.24E-05	29.4	Missense o/e =0.97<br>(CI 0.89 - 1.06)
		Chr14:88452941T>C:NM_001201401.2:c.265A>G:p.T89A<br>Heterozygous, not paternal	2.50E-03	23	Missense o/e = 0.97<br>(CI 0.89 - 1.06)

P183	*SCO1, GAS7*	Chr17:10062326_10600121dup<br>Not maternal	0	.	GAS7 pLOF o/e = 0.18<br>(CI 0.1 - 0.36)
P185	*CLCN2*	Chr3:184071575C>T:NM_001171088.3:c.1598G>A:p.R533Q<br>Heterozygous, inheritance unknown	2.00E-04	24	Missense o/e = 0.92<br>(CI 0.85 - 0.99)
	CACNA1C	Chr12: 2719716G>T:NM_000719.7:c.3568G>T:p.V1190L<br>Heterozygous, inheritance unknown	1.22E-05	27.6	Missense o/e = 0.5<br>(CI 0.46 - 0.53)
P187	*ROCK2*	Chr2:11104502_11337616dup<br>Inheritance unknown	0	.	.
P188	*COL4A4*	Chr2:227872823G>A:NM_000092.5: c.4720C>T:p.Q1574*<br>Heterozygous, inheritance unknown	0	46	pLOF o/e = 0.47<br>(CI 0.37 - 0.62)
P189	NCOR1	Chr17:16042475A>T:NM_001190438.1:c.872T>A:p.L291H<br>Heterozygous, not maternal	0	27.6	Missense o/e = 0.7<br>(CI 0.66 - 0.74)
P191	*KCNH1*	Chr1:211263967A>C:NM_002238.4:c.376T>G:p.F126V<br>Heterozygous, maternal, Sibling 1 also carries	0	27.7	Missense o/e = 0.55<br>(CI 0.5 - 0.61)
	ADCY6	Chr12:49170894C>G:NM_015270.5: c.1369G>C:p.A457P<br>Heterozygous, not maternal, not carried by siblings	0	30	Missense o/e = 0.73<br>(CI 0.68 - 0.78)
P193	*CHD7*	Chr8:61655217C>T:NM_001316690.1:c.1226C>T:p.P409L<br>Heterozygous, paternal	0	30	Missense o/e = 0.77<br>(CI 0.74 - 0.81)
	COL5A2	Chr2:189916185G>A:NM_000393.5:c.2792C>A:p.P931H<br>Heterozygous, paternal	0	29.9	Missense o/e = 0.77<br>(CI 0.72 - 0.82)
P199	*F8*	ChrX:154156919G>T:NM_000132.3:c.5146C>A:p.H1716N<br>Inheritance unconfirmed, Father haemophilia A	0	25.5	Missense o/e = 0.76<br>(CI 0.71 - 0.81)
	NECTIN2	Chr19:45368566C>T:NM_001042724.2:c.127C>T:p.R43*<br>Heterozygous, inheritance unknown	0	35	pLOF o/e = 0.12<br>(CI 0.06 - 0.32)
P201	*TLR7*	ChrX:12906598C>A:NM_016562.4:c.2971C>A:p.L991I<br>Hemizygous, inheritance unknown	2.18E-05	25.3	Missense o/e = 0.56<br>(CI 0.5-0.63)
	FERMT3	Chr11:63990566C>T:NM_031471.6:c.1717C>T:p.R573*<br>Heterozygous, inheritance unknown	0	42	pLOF o/e = 0.35<br>(CI 0.23-0.56)
P205	*CFTR*	Chr7:117199645TCTT>TNM_000492.4:c.1520_1522del:<br>p.P508del<br>Homozygous, maternal, no contact with father	7.17E-03	21.3	Missense o/e = 1.32<br>(CI 1.25 - 1.39)
	CLCN1	Chr7:143048771C>T:NM_000083.3:c.2680C>T:p.R894*<br>Heterozygous, not maternal	3.18E-03	35	pLOF o/e=0.11<br>(CI 0.05-0.28)
P211	*IL1RAP*	Chr3:190374214C>G:NM_001167931.2:c.1882C>G:p.R628G<br>Heterozygous, inheritance unknown	0	24.5	Missense o/e = 0.74<br>(CI 0.67-0.81)

	ITPR3	Chr6:33652174A>T:NM_002224.4: c.4978A>T:p.K1660*<br>Heterozygous, inheritance unknown	0	48	pLOF o/e = 0.46<br>(CI 0.37-0.57)
P214	*15q11-q13 dup*	Chr15:22722801_26749200dup<br>Validated by array, likely larger event than this	0	.	.
P217	*SPAST*	Chr2:32347645_32354557del<br>Heterozygous, paternal	0	.	pLOF o/e = 0.09<br>(CI 0.04 - 0.22)
	SPAST	Chr2:32289031C>T:NM_014946.4:c.131C>T:p.S44L<br>Heterozygous, maternal	4.54E-03	21.2	Missense o/e = 0.81<br>(CI 0.73 - 0.9)
P220	*TNR*	Chr1:175372696A>G:NM_003285.3:c.556T>C:p.C186R<br>Heterozygous, inheritance unknown	0	28.4	Missense o/e = 0.84<br>(CI 0.79 - 0.9)
P225	*SPAST*	Chr2:32370014A>G:NM_014946.4:c.1625A>G:p.D542G<br>Heterozygous, maternal, both siblings share variant	4.13E-04	21.2	Missense o/e = 0.81<br>(CI 0.73 - 0.9)
	FBN1	Chr15:48717607G>C:NM_000138.4:c.7412C>G:p.P2471R<br>Heterozygous, maternal, siblings do not share variant	2.48E-05	31	Missense o/e = 0.64<br>(CI 0.61-0.68)
	ICAM5	Chr19:10407242G>A:NM_003259.4:c.2725G>A:p.E909K<br>Paternal, shared by sibling 2	0	27.6	Missense o/e=0.69<br>(CI 0.63 - 0.75)
	HERC2	Chr15:28358378A>G:NM_004667.6: c.14071T>C: p.Y4691H<br>Heterozygous, paternal, siblings do not share	0	24.1	Missense o/e = 0.76<br>(CI 0.73 - 0.79)
P226	*SRGAP1*	Chr12:64505574A>G:NM_001346201.2:c.1883A>G:p.D628G<br>Heterozygous, paternal	0	28.4	Missense o/e= 0.75<br>(CI 0.69 - 0.81)
P228	*ASTN2*	Chr9:119495729G>A:NM_014010.5:c.2317C>T:p.Q773*<br>Heterozygous, paternal	0	46	pLOF o/e = 0.14<br>(CI 0.08 - 0.25)
P229	*BCOR*	ChrX:39934249T>C:NM_001123383.1:c.350A>G:p.E117G<br>Heterozygous, maternal	0	25.7	Missense o/e = 0.81<br>(CI 0.75 - 0.86)
P230	*WNK3*	ChrX:54275132G>A:NM_001002838.4:c.3649C>T:p.H1217Y<br>Hemizygous, X-linked	4.66E-05	23.8	Missense o/e = 0.71<br>(CI 0.66-0.77)
P231	*SUN1*	Chr7:892253C>T:NM_001171944:c.553C>T:p.R185*<br>Heterozygous, maternal	4.01E-06	38	pLOF o/e = 0.37<br>(CI 0.26-0.56)
	TENM2	Chr5:167655125G>C:NM_001080428.3:c.4793G>C:p.R1598T<br>Heterozygous, not maternal	1.62E-05	32	Missense o/e = 0.77<br>(CI 0.74-0.81)
	CDNF	Chr10:14879836C>G:NM_001029954.3:c.110G>C:p.C37S<br>Heterozygous, maternal	0	27.3	Missense o/e = 0.92<br>(CI 0.78-1.09)
P232	*EGFR*	Chr7:55223558C>T:NM_001346941.2:c.124C>T:p.R42*<br>De novo, not shared by 3 siblings	0	38	pLOF o/e = 0.23<br>(CI 0.15-0.35)

P233	*SYNE2*	Chr14:64628848C>T:NM_015180.6: c.16153C>T:p.Q5385*<br>Heterozygous, inheritance unknown	0	45	pLOF o/e = 0.32<br>(CI 0.27 - 0.37)
P235	*BCOR*	ChrX:39933749C>T:NM_001123383.1:c.850G>A:p.D284N<br>Hemizygous, inheritance unknown	3.2E-05	26.3	Missense o/e = 0.81<br>(CI 0.75-0.86)
	WDR47	Chr1:109538428C>T:NM_001142550.2:c.1489G>A:p.G497R<br>Heterozygous, inheritance unknown	3.98E-06	26.8	Missense o/e = 0.65<br>(CI 0.59-0.71)
P236	*TUBB4A*	Chr19:6495282C>T:NM_006087.4:c.1228G>A:p.E410K<br>Heterozygous, de novo	0	27.5	Missense o/e = 0.31<br>(CI 0.26-0.37)
P701	*KCTD17*	Chr22:37453523G>A:NM_001282684.1:c.497G>A:p.R166H<br>Maternal, heterozygous	1.21E-05	26.9	Missense o/e =0.64<br>(CI 0.54-0.76)
P703	*SPG7*	Chr16:89598369G>A:NM_003119.4:c.1045G>A:p.G349S<br>Heterozygous, Inheritance unknown	8.23E-04	26.7	Missense o/e =1.11<br>(CI 1.03-1.2)
P704	*CACNA1S*	Chr1:201052419G>A:NM_000069.3:c.1264C>T:p.R422C<br>Heterozygous, maternal	1.59E-05	33	Missense o/e = 1.01<br>(CI 0.96 - 1.06)
P706	*AGAP2*	Chr12:58120493C>G:NM_014770.4:c.2353G>C:p.D785H<br>Heterozygous, inheritance unknown	0	29.3	Missense o/e = 0.62<br>(CI 0.57 - 0.68)
P708	*KMT2B*	Chr19:36214767G>A:NM_014727.3: c.3193G>A:p.E1065K<br>Heterozygous, maternal	0	25.1	Missense o/e = 0.76<br>(CI 0.72 - 0.8)
	KLHL3	Chr5:136961485C>T:NM_001257195.2:c.1446G>A:p.W482*<br>Heterozygous, not maternal	0	38	pLOF o/e = 0.16<br>(CI 0.08 - 0.34)
P710	*F2*	Chr11:46761055G>A:NM_001311257.2:c.*97G>A<br>Heterozygous, inheritance unknown	8.44E-03	.	Missense o/e = 0.7<br>(CI 0.63 - 0.77).
P711	*DIP2A*	Chr21:47966897G>T:NM_001146115.2: c.2335G>T:p.D779Y<br>Heterozygous, inheritance unknown	0	32	Missense o/e = 0.76<br>(CI 0.72 - 0.81)
P712	*FOXP1*	Chr3:71027104G>A:NM_001244813.3:c.923C>T:p.T308M<br>Heterozygous, inheritance unknown	0	27.9	Missense o/e = 0.67<br>(CI 0.6 - 0.74)
	NF1	Chr17:28992701_30408700del<br>Het, inheritance unknown	0	.	pLOF o/e = 0.22<br>(CI 0.16 - 0.29)
P714	*STXBP1*	Chr9:130425629G>A:NM_003165.6:c.575G>A:p.R192Q<br>Heterozygous, maternal	3.98E-06	29.5	Missense o/e = 0.36<br>(CI 0.31 - 0.42)
P715	*TTN*	Chr2:179476563G>A:NM_001256850.1:c.45550C>T:p.Q15184*<br>Heterozygous, maternal	0	61	pLOF o/e = 0.33<br>(CI 0.3 - 0.35)
P720	*EML2*	Chr19:46130010A>G:NM_001193268.3:c.1297T>C:p.W433R<br>Heterozygous, not maternal	0	28.6	Missense o/e = 0.98<br>(CI 0.91 - 1.06)

	PIH1D3	ChrX:106466003G>A:NM_173494.2:c.361G>A:p.G121R<br>Hemizygous, X-linked	3.4E-05	24.7	Missense o/e = 0.84<br>(CI 0.68 - 1.04)
P721	*MATN3*	Chr2:20206009G>A:NM_002381.5:c.286C>T:p.P96S<br>Heterozygous, maternal	1.09E-05	26.8	Missense o/e = 0.86<br>(CI 0.76 - 0.96)
	GALC	Chr14:88412026A>G:NM_001201401.2:c.1472T>C:p.F491S<br>Heterozygous, not maternal	1.21E-05	31	Missense o/e = 0.97<br>(CI 0.89 - 1.06)
P730	*SRCAP*	Chr16:30748569G>A:NM_006662.3:c.7208G>A:p.R2403Q<br>Heterozygous, not maternal	0	27.6	Missense o/e = 0.86<br>(CI 0.83 - 0.9)
	CNTN6	Chr3:1427412G>T:NM_014461.4:c.2635G>T:p.V879L<br>Heterozygous, not maternal	0	26	Missense o/e = 1.36<br>(CI 1.28 - 1.45)
P737	*UBQLN2*	ChrX:56591528C>G:NM_013444.4:c. 1222C>G:p.Q408E<br>Hemizygous, inheritance unknown	0	25.9	Missense o/e = 0.73<br>(CI 0.64 - 0.82)
	KAZALD1	Chr10:102822755G>C:NM_030929.5:c.406G>C:p.G136R<br>Heterozygous, not paternal	0	33	Missense o/e = 0.97<br>(CI 0.86 - 1.11)
P739	*ACTN1*	Chr14:69343866C>T:NM_001102.4:c.2453G>A:p.R818H<br>Heterozygous, not maternal	0	33	Missense o/e = 0.61<br>(0.56 - 0.67)
	PROC	Chr2:128186457T>C:NM_000312.4:c.1321T>C:p.Y441H<br>Heterozygous, not maternal	3.9E-05	28.3	Missense o/e = 0.85<br>(CI 0.76 - 0.95)
P740	*DNMT1*	Chr19:10247869G>A:NM_001130823.3:c.4381C>T:p.R1461W<br>Heterozygous, paternal	0	33	Missense o/e = 0.55<br>(CI 0.51 - 0.59)
P741	*MITF*	Chr3:70008431C>T:NM_006722.3:c.1018C>T:p.R340C<br>Heterozygous, inheritance unknown	0	33	Missense o/e = 0.77<br>(CI 0.69-0.85)
P743	*CHD9*	Chr16:53358554T>C:NM_001308319.2:c.8441T>C:p.L2814P<br>Heterozygous, not maternal	0	24	Missense o/e = 0.72<br>(CI 0.69 - 0.76)
P746	*ABCA4*	Chr1:94466628G>A:NM_000350.3:c.6316C>T:p.R2106C<br>Heterozygous, inheritance unknown	1.31E-04	33	Missense o/e = 1.05<br>(CI 1 - 1.1)
	ABCA4	Chr1:94481325G>C:NM_000350.3:c.5282C>G:p.P1761R<br>Heterozygous, inheritance unknown	0	26.5	Missense o/e = 1.05<br>(CI 1 - 1.1)
P747	*ARHGAP32*	Chr11:129034251C>T:NM_001142685.2:c.188G>A:p.R63Q<br>Heterozygous, inheritance unknown	0	27	Missense o/e = 0.85<br>(CI 0.81 - 0.9)
P748	*LDB3*	Chr10:88478533G>A:NM_001080114.2:c.1577G>A:p.C526Y<br>Heterozygous, inheritance unknown	0	31	Missense o/e = 0.96<br>(CI 0.89 - 1.04)
	CCDC88C	Chr14:91757409G>C:NM_001080414.4:c.4132C>G:p.R1378G<br>Heterozygous, inheritance unknown	0	26.1	Missense o/e = 0.94<br>(CI 0.9 - 0.99)

P749	*GJB2*	Chr13:20763626C>T:NM_004004.6:c.95G>A:p.R32H<br>Heterozygous, paternal	3.99E-06	25.3	Missense o/e = 1.17<br>(CI 1.03 - 1.34)
	FGFR1	Chr8:38270868_38270907del<br>Heterozygous, paternal	0	.	pLOF o/e = 0.09<br>(CI 0.04 - 0.21)
	ATP1A3	Chr19:42485704G>A:NM_001256213.2: c.1420C>T: p.R474C<br>Heterozygous, ,maternal	4.49E-04	28.9	Missense o/e = 0.29<br>(CI 0.26 - 0.33)
P750	*VCX3A, STS, PNPLA4*	ChrX:6451301_8138000del<br>Hemizygous, inheritance unknown	0	.	.
	MARVELD3	Chr16:71674560G>A:NM_001017967.4:c.863G>A:p.G288E<br>Heterozygous, not maternal	0	26.5	Missense o/e = 0.94<br>(CI 0.84 - 1.04)
		Chr16:71674692A>G:NM_001017967.4:c.995A>G:p.D332G<br>Heterozygous, maternal	0	23.9	Missense o/e = 0.94<br>(CI 0.84 - 1.04)
P751	*CNKSR2*	ChrX:21545084A>T:NM_001168649.3:c.910A>T:p.I304F<br>Heterozygous, maternal	5.49E-06	26.4	Missense o/e = 0.47<br>(CI 0.42 - 0.54)
P752	*NKX2-6*	Chr8:23560414dup:NM_001136271.3:c.455dup:p.Q153Afs*207<br>Heterozygous, inheritance unknown	3.15E-05	30	pLOF o/e = 0.45<br>(CI 0.22 - 1.03)
P753	*DOCK6*	Chr19:11323875G>A:NM_020812.4:c.4468C>T:p.R1490*<br>Heterozygous, inheritance unknown	0	47	pLOF o/e = 0.58<br>(CI 0.47 - 0.72)
	CNTNAP2	Chr7:147869373G>T:NM_014141.6:c.2813G>T:p.R938L<br>Heterozygous, inheritance unknown	0	33	Missense o/e = 1.03<br>(CI 0.97 - 1.09)
P754	*KIDINS220*	Chr2:8871669del: NM_020738.2: c.4497del: p.R1499Sfs*9<br>Heterozygous, not maternal, father deceased	0	.	pLOF o/e = 0.24<br>(CI 0.17 - 0.35)
P756	*CACNA1A*	Chr19:13318399C>A:NM_001127222.2:c.7249G>T:p.E2417*<br>Heterozygous, not maternal	0	40	pLOF o/e = 0.08<br>(CI 0.04 - 0.13)
P758	*ZNF74, USP41*	Chr22:20744491_20758257dup<br>Validated by qPCR, duplication only of *ZNF74*	0	.	.
P759	*SETX*	Chr9:135172393_135172402del:<br>NM_001351527.2:c.5821_5830del:p.A1941Lfs*6<br>Heterozygous, not maternal, Father unavailable	0	.	pLOF o/e = 0.21<br>(CI 0.14 - 0.3)
P760	*PNPLA6*	Chr19:7620610insGCCA NM_001166114.2: c.3058_3061dup:<br>p.R1021Qfs*38<br>Heterozygous, inheritance unknown	1.25E-04	35	pLOF o/e = 0.48<br>(CI 0.36 - 0.64)
		Chr19:7606546delG:NM_001166114.2: c.1144del: p.A383Pfs*11<br>Heterozygous, inheritance unknown	0	.	pLOF o/e = 0.48<br>(CI 0.36 - 0.64)

P761	*SPG11*	Chr15:44876084del:NM_001160227.2:c.5794del:p.H1932Mfs*19<br>Heterozygous, not paternal, Mother not available	0	.	pLOF o/e = 0.67<br>(CI 0.56 - 0.81)
P763	*CPA6*	Chr8:68396042C>T:NM_020361.5:c.799G>A:p.G267R<br>Heterozygous, inheritance unknown	2.06E-03	29.1	Missense o/e = 1.09<br>(CI 0.98 - 1.21)
		Chr8:68419039G>C:NM_020361.5:c.619C>G:p.Q207E<br>Heterozygous, inheritance unknown	1.43E-03	26.2	Missense o/e = 1.09<br>(CI 0.98 - 1.21)
	STRADA	Chr17:61800688T>G:NM_001003787.4:c.95-2A>C Splicing<br>Heterozygous, inheritance unknown	8.23E-06	25.8	pLOF o/e = 0.5<br>(CI 0.33 - 0.8)
	REM2	Chr14:23355873G>A:NM_173527.3:c.778G>A:p.A260T<br>Heterozygous, inheritance unknown	0	34	Missense o/e = 0.75<br>(CI 0.65 - 0.86)
P769	*CCDC88C*	Chr14:91739384G>T:NM_001080414.4:c.5672C>A:p.P1891Q<br>Heterozygous, not paternal	0	25.6	Missense o/e = 0.94<br>(CI 0.9 - 0.99)
P772	*MYH4*	Chr17:10360830C>G:NM_017533.2:c.1804G>C:p.D602H<br>Heterozygous, not maternal, Father not available	0	27	Missense o/e = 0.99<br>(CI 0.94 - 1.04)
	COL6A3	Chr2:238249283A>T:NM_057166.5:c.6455T>A:p.I2152N<br>Heterozygous, maternal	0	28.4	Missense o/e = 1.04<br>(CI 1 - 1.08)
	PDGFRB	Chr5:149498359G>C:NM_002609.4:c.2855C>G:p.S952C<br>Heterozygous, maternal	2.39E-05	28.1	Missense o/e = 0.8<br>(CI 0.75 - 0.86)
P773	*ZFHX3*	Chr16:72821397G>C:NM_001164766.2: c.8036C>G:p.S2679*<br>Heterozygous, maternal	0	45	pLOF o/e = 0.08<br>(CI 0.05 - 0.14)
P775	*ATF4*	Chr22:39917925C>T:NM_001675.4:c.374C>T:p.P125L<br>Homozygous, IBD	0	22.8	Missense o/e = 0.7<br>(CI 0.6 - 0.83)
	NAA15	Chr4:140278588A>G:NM_057175.5:c.1136A>G:p.Y379C<br>Heterozygous, inheritance unknown	0	27	Missense o/e = 0.48<br>(CI 0.43 - 0.54)
P776	*RAB5C*	Chr17:40280747G>A:NM_004583.4:c.238C>T:p.Q80*<br>Heterozygous, inheritance unknown	0	38	pLOF o/e = 0.08<br>(CI 0.03 - 0.36)
P778	*VWF*	Chr12:6143978C>T:NM_000552.5: c.2561G>A:p.R854Q<br>Heterozygous, inheritance unknown	3.47E-03	33	pLOF o/e = 0.93<br>(CI 0.89 - 0.97)
P779	*F2*	Chr11:46747447G>A:NM_000506.5: c.598G>A:p.E200K<br>Heterozygous, inheritance unknown	1.20E-03	0	Missense o/e = 0.7<br>(CI 0.63 - 0.77)
P780	*NECAP1*	Chr12:8242570G>C:NM_015509.4: c.134G>C:p.G45A<br>Heterozygous, not maternal	0	29.2	Missense o/e = 0.69<br>(CI 0.59 - 0.81)
P782	*ARFGAP2*	Chr11:47196804G>A:NM_001242832.2:c.325C>T:p.R109*<br>Heterozygous, maternal	3.98E-06	37	Missense o/e = 0.32<br>(CI 0.2 - 0.54)

	AHDC1	Chr1:27873829C>G:NM_001029882.3:c.4798G>C:p.V1600L<br>Heterozygous, maternal	0	26.5	Missense o/e = 0.75<br>(CI 0.7 - 0.79)
P783	*ZFYVE26*	Chr14:68220884C>A:NM_015346.4: c.7032G>T:p.L2344F<br>Heterozygous, inheritance unknown	0	23	Missense o/e = 0.96<br>(CI 0.92 - 1)
		Chr14:68268930C>T:NM_015346.4: c.1505G>A:p.C502Y<br>Heterozygous, inheritance unknown	0	29.5	Missense o/e = 0.96<br>(CI 0.92 - 1)
	RAPGEFL1	Chr17:38348507A>C:NM_001303533.2:c.1195A>C:p.N399H<br>Homozygous, Likely IBD	0	25.8	Missense o/e = 0.7<br>(CI 0.62 - 0.79)
P784	*GNB1*	Chr1:1737942A>G:NM_001282539.2:c.239T>C:p.I80T<br>Heterozygous, inheritance unknown	3.98E-06	25.6	Missense o/e = 0.28<br>(CI 0.23 - 0.35)
P785	*DOCK8, KANK1*	Chr9:293723_686363dup<br>Inheritance unknown	0	.	DOCK8 pLOF o/e =0.36<br>(CI 0.28 - 0.48)
P786	*ITPR1*	Chr3:4753542A>G:NM_001168272.2:c.5048A>G:p.Y1683C<br>Heterozygous, maternal	0	29.3	Missense o/e = 0.59<br>(CI 0.56 - 0.63)
	ELOVL4	Chr6:80626473T>C:NM_022726.2:c.797A>G:p.Y266C<br>Heterozygous, maternal	0	29	Missense o/e = 0.74<br>(CI 0.64 - 0.86)
	BSCL2	Chr11:62472963G>A:NM_001122955.4:c.214C>T:p.P72S<br>Heterozygous, maternal	0	26.1	Missense o/e = 0.95<br>(CI 0.86 - 1.06)
P788	*WWOX*	Chr16:78269608_78348628del<br>Heterozygous, inheritance unknown	0	.	pLOF o/e = 1.07<br>(CI 0.77 - 1.53)
P789	*CHRNB2*	Chr1:154544167A>T:NM_000748.3: c.868A>T:p.T290S<br>Heterozygous, maternal	0	24.8	Missense o/e = 0.67<br>(CI 0.6 - 0.75)
	CHRNA7	Chr15:32456401_32475000dup<br>Inheritance unknown	0	.	pLOF o/e = 0.44<br>(CI 0.26 - 0.77)
P790	*IKBKG*	ChrX:153792196C>T:NM_001145255.4:c.781C>T:p.H261Y<br>Hemizygous, inheritance unknown	0	23.3	Missense o/e = 0.99<br>(CI 0.79 - 1.24)
P792	*COL4A1*	Chr13:110850841C>T:NM_001303110.2:c.1258G>A:p.G420R<br>Heterozygous, inheritance unknown	0	18.3	Missense o/e = 0.73<br>(CI 0.68 - 0.77)
P795	*CAMTA1*	Chr1:7811383G>A:NM_001349613.1:c.1943G>A:p.R648Q<br>Heterozygous, inheritance unknown	4.03E-06	33	Missense o/e = 0.71<br>(CI 0.67 - 0.76)
	F2	Chr11:46761055G>A:NM_001311257.2:c.*97G>A<br>Heterozygous, inheritance unknown	8.44E-03	.	Missense o/e = 0.7<br>(CI 0.63 - 0.77).
P796	*PIEZO2*	Chr18:10763065G>T:NM_022068.4:c.2903C>A:p.S968Y<br>Heterozygous, paternal	0	25.3	Missense o/e = 0.75<br>(CI 0.71 - 0.78)

P798	*PEX1*	Chr7:92132483dup:NM_001282677.2:c.1926dup:p.I643Yfs*42 Heterozygous, not maternal	4.85e-4	27.6	pLOF o/e = 0.5 (CI 0.38 - 0.66)
	PEX14	Chr1:10596309C>T:NM_004565.2:c.124C>T:p.R42C Heterozygous, maternal	1.06e-5	26.4	Missense o/e = 0.76 (CI 0.67 - 0.86)
P799	*GNAO1*	Chr16:56377748A>C:NM_138736.3:c.951A>C:p.K317N Heterozygous, not maternal	0	29.2	Missense o/e = 0.41 (CI 0.34 - 0.48)
P801	*CDK13*	Chr7:39990904C>T:NM_003718.5:c.664C>T:p.Q222* Heterozygous, maternal	0	35	pLOF o/e = 0.2 (CI 0.12 - 0.32)
P802	*EFEMP1*	Chr2:56098226G>A:NM_001039349.3:c.1033C>T:p.R345W Heterozygous, not maternal	0	25.9	Missense o/e = 0.69 (CI 0.61 - 0.78)
	PCBP3	Chr21:47329390A>G:NM_001130141.2:c.461A>G:p.E154G Heterozygous, not maternal	0	33	Missense o/e = 0.63 (CI 0.55 - 0.72)
P902	*CAMTA1*	Chr1:7796498C>T:NM_001349613.1:c.290C>T:p.S97F Heterozygous, not maternal	0	32	Missense o/e = 0.71 (CI 0.67 - 0.76)
P904	*SCN8A*	Chr12:52188354C>T:NM_014191.4:c.4724C>T:p.A1575V De novo, shared by twin	4.01E-06	29.5	Missense o/e = 0.35 (CI 0.32 - 0.38)
P905	*DST*	Chr6:56463369T>C:NM_015548.5:c.A3964G:p.K1322E Heterozygous, maternal	0	25.2	Missense o/e = 0.88 (CI 0.84 - 0.91)
P907	*TBX2*	Chr17:59482700C>T:NM_005994.4:c.1189C>T:p.R397W Heterozygous, not maternal	0	28.9	Missense o/e = 0.78 (CI 0.7 - 0.85)
P910	*TUBA1A*	Chr12:49580570C>T:NM_001270399.2:c.50G>A:p.G17D Heterozygous, inheritance unknown	0	29.2	Missense o/e = 0.03 (CI 0.01 - 0.05)
P911	*COL4A2*	Chr13:111145620G>A:NM_001846.4: c.3625G>A:p.G1209R Heterozygous, paternal	0	24.7	Missense o/e = 0.81 (CI 0.76 - 0.86)
P913	*SLC9A5*	Chr16:67283034G>A:NM_001323971.2:c.117G>A:p.W39* Heterozygous, maternal	0	36	pLOF o/e = 0.48 (CI 0.33 - 0.69)
	SPG7	Chr16:89613145C>T:NM_003119.4:c.1529C>T:p.A510V Heterozygous, not maternal	0.027	32	Missense o/e = 1.11 (CI 1.03 - 1.2)
P918	*USP32*	Chr17:58292096A>T:NM_032582.4: c.1907T>A:p.L636* Heterozygous, not maternal	0	38	pLOF o/e = 0.14 (CI 0.09 - 0.22)
	KCNH5	Chr14:63453878C>T:NM_139318.5: c.461G>A:p.R154Q Heterozygous, not maternal	1.06E-05	29.1	Missense o/e = 0.7 (CI 0.64 - 0.76)
	DEAF1	Chr11:687962C>T:NM_001293634.1:c.613G>A:p.V205I Heterozygous, not maternal	2.79E-05	24.5	Missense o/e = 0.77 (CI 0.69 - 0.85)

P926	*NOTCH3*	Chr19:15276711C>T:NM_000435.3:c.5554G>A:p.A1852T<br>Heterozygous, inheritance unknown	1.59E-05	26.9	Missense o/e = 0.74<br>(CI 0.7 - 0.77)
P931	*ALDH3A2*	Chr17:19663333_19663335delinsGGGCTAAAAGTACTGTTGGGG:<br>NM_000382.3: c.941_943delinsGGGCTAAAAGTACTGTTGGGG:<br>p.A314_P315delinsGAKSTVGA<br>Homozygous, maternal, inferred IBD.	0	.	Missense o/e = 0.51<br>(CI 0.33 - 0.82)
P934	*NOTCH3*	Chr19:15276824T>A:NM_000435.3: c.5441A>T:p.D1814V<br>Heterozygous, not maternal	0	25.9	Missense o/e = 0.74<br>(CI 0.7 - 0.77)
	KCNA6	Chr12:4920526C>T:NM_002235.4:c.1319C>T:p.S440L<br>Heterozygous, not maternal	0	33	Missense o/e = 0.53<br>(CI 0.47 - 0.6)
P936	*FGFR2*	Chr10:123247626C>T:NM_001144914.1:c.1529G>A:p.C510Y<br>Heterozygous, maternal	0	34	Missense o/e = 0.69<br>(CI 0.63 - 0.75)
P938	*NOTCH1*	Chr9:139409789A>G:NM_017617.5: c.1967T>C:p.L656P<br>Heterozygous, not maternal	0	22.8	Missense o/e = 0.76<br>(CI 0.73 - 0.8)
P939	*PDGFRB*	Chr5:149502705G>A:NM_002609.4:c.2083C>T:p.R695C<br>Heterozygous, maternal	1.13E-04	32	Missense o/e = 0.8<br>(CI 0.75 - 0.86)
	PROC	Chr2:128179014G>A:NM_000312.4: c.226G>A:p.V76M<br>Heterozygous, not maternal	4.96E-05	21.2	Missense o/e = 0.85<br>(CI 0.76 - 0.95)
P940	*VAMP1*	Chr12:6580127_6584844del<br>Heterozygous, inheritance unknown	0	.	pLOF o/e = 0.54<br>(CI 0.28 - 1.15)
P943	*STRIP1*	Chr1:110596480G>A:NM_001270768.2:c.2175G>A:p.W725*<br>Heterozygous, inheritance unknown	0	44	pLOF o/e = 0.37 (CI 0.25<br>- 0.55)
P944	*TAF1*	ChrX:70601671G>A:NM_004606.3:c.1499G>A:p.R500H<br>Hemizygous, inheritance unknown	1.72E-05	29.4	Missense o/e = 0.44<br>(CI 0.4 - 0.48)
P945	*SPTBN2*	Chr11:66475075G>C:NM_006946.4:c.1565C>G:p.A522G<br>Heterozygous, inheritance unknown	0	24.1	Missense o/e = 0.81<br>(CI 0.77 - 0.85)
	PCBP3	Chr21:47131753_47242245del<br>Heterozygous, inheritance unknown	0	.	pLOF o/e = 0.17<br>(CI 0.08 - 0.39)
P947	*NIPA1*	Chr15:23088461_23131516del<br>Heterozygous, inheritance unknown	0.002	.	pLOF o/e = 0.49<br>(CI 0.26 - 1.04)
P948	*ANKLE2*	Chr12:133325764_133335225dup<br>Heterozygous, inheritance unknown	0	.	pLOF o/e = 0.59<br>(CI 0.42 - 0.85).
P953	*EMC1*	Chr1:19568838C>A:NM_001271428.2:c.509+1G>T Splicing	0	34	pLOF o/e =0.79<br>(CI 0.62-1.01)

P955	*KMT2E*	Chr7:104752315A>G:NM_018682.4:c.4112A>G:p.K1371R<br>Heterozygous, inheritance unknown	0	24.6	Missense o/e = 0.87<br>(CI 0.82 - 0.92)
	VPS53	Chr17:419889_420996del<br>Heterozygous, inheritance unknown	0	.	pLOF o/e = 0.42<br>(CI 0.29 - 0.61).
	SPTBN2	Chr11:66456209T>C:NM_006946.4:c.6146A>G:p.E2049G<br>Heterozygous, inheritance unknown	0	25.9	Missense o/e = 0.81<br>(CI 0.77 - 0.85)
P957	*MFN2*	Chr1: 12064892G>A:NM_001127660.2:c.1403G>A:p.R468H<br>Heterozygous, inheritance unknown	2.5E-03	27.6	pLOF o/e = 0.13<br>(CI 0.07 - 0.28)
P962	*SPG7*	Chr16:89613145C>T:NM_003119.4:c.1529C>T:p.A510V<br>Heterozygous, inheritance unknown	0.0029	26.8	Missense o/e =1.11<br>(CI 1.03 - 1.2)
P965	*COL4A1*	Chr13:110817245C>G:NM_001845.6:c.4114G>C:p.G1372R<br>Heterozygous, inheritance unknown	0	23.9	Missense o/e =0.73<br>(CI 0.68 - 0.77)
P972	*MT-TL1*	ChrM:3243A>G: NC_012920.1: m.3243A>G<br>Low level detectable in maternal blood-derived DNA	.	.	.
P974	*TRIO*	Chr5:14480080A>T:NM_007118.4:c.6296A>T:p.K2099I<br>Heterozygous, inheritance unknown	0	32	Missense o/e = 0.64<br>(CI 0.61 - 0.68)
	CHD8	Chr14:21897482G>A:NM_001170629.2:c.856C>T:p.R286C<br>Heterozygous, inheritance unknown	2.45e-5	32	Missense o/e = 0.56<br>(CI 0.53 - 0.59)
P980	*22q11.2 dup*	Chr22:18873001_19571600dup<br>Validated by array, event likely larger than this	0	.	.
P983	*RASGEF1B*	Chr4:82363492T>A:NM_152545.3:c.967A>T:p.T323S<br>Heterozygous, inheritance unknown	0	29.4	Missense o/e= = 0.75<br>(CI 0.66 - 0.84)
P1105	*WNK4*	Chr17:40947642G>A:NM_032387.5:c.3023-1G>A Splicing<br>Heterozygous, maternal	3.98E-06	27	pLOF o/e = 0.81<br>(CI 0.64 - 1.05)
P1106	*PIEZO2*	Chr18:10797455del:NM_022068.4:c.1444del:p.R482Efs*16<br>Heterozygous, not maternal, not shared by sibling, father not available	0	.	pLOF o/e = 0.28<br>(CI 0.22 - 0.37)
P1110	*COL4A2*	Chr13:111092182T>C:NM_001846.2:c.957+2T>C Splicing<br>Heterozygous, not maternal, father not available	0	25	pLOF o/e = 0.45<br>(CI 0.35 - 0.58)
P1114	*AXL*	Chr19:41748919G>T:NM_001278599.2:c.640G>T:p.G214*<br>Heterozygous, inheritance unknown	0	41	pLOF o/e = 0.27<br>(CI 0.18 - 0.44)
	PDGFA	Chr7:552072del:NM_002607.6:c.181del:p.T61Pfs*3<br>Heterozygous, inheritance unknown	0	.	pLOF o/e = 0.1 (CI 0.04 - 0.48)
P1123	*SYT14*	Chr1:210056221_210262039dup<br>Maternal	0	.	pLOF o/e = 0.15<br>(CI 0.07 - 0.34)

P1129	*THRA*	Chr17:38233862insGCTTCTTTCGCCGCACAATCCAGAAGAACCTCCATCCCACCTATTCCTGCAAA:NM_001190919.2: c.222+1insGCTTCTTTCGCCGCACAATCCAGAAGAACCTCCATCCCACCTATTCCTGCAAA Splicing	0	.	pLOF o/e = 0.23 (CI 0.13 - 0.45)
P1130	*EXD1*	Chr15:41482310C>T:NM_152596.4:c.707G>A:p.W236* Heterozygous, paternal, not shared by sibling	0	39	pLOF o/e = 0.88 (CI 0.63 - 1.26)
	EXD1	Chr15:41502420_41511953del Heterozygous, paternal	0	.	
P1132	*BUB1B*	Chr15:40493140C>A:NM_001211.6:c.1526C>A:p.S509* Heterozygous, maternal	0	36	pLOF o/e = 0.42 (CI 0.31 - 0.58)
	FIGN	Chr2:164466649T>G:NM_018086.4:c.1693A>C:p.K565Q Heterozygous, maternal	0	24.8	Missense o/e = 0.84 (CI 0.77 - 0.92)
P1134	*HTRA2*	Chr2:74759053G>C:NM_013247.5:c.1115+1G>C Splicing Heterozygous, not paternal	0	32	pLOF o/e = 0.4 (CI 0.23 - 0.76)
P1136	*FARS2*	Chr6:5369212A>G:NM_001318872.2:c.409A>G:p.S137G Heterozygous, not maternal	3.98E-06	25.5	Missense o/e = 0.88 (CI 0.79 - 0.98)
P1137	*CTNNB1*	Chr3:40831837_41291740dup Not maternal	0	.	pLOF o/e = 0.03 (CI 0.01 - 0.13)
P1138	*COL4A2*	Chr13:111155739G>A:NM_001846.4: c.4049G>A:p.G1350D Heterozygous, maternal	0	25.6	Missense o/e = 0.81 (CI 0.76 - 0.86)
P1140	*CLIP2*	Chr7:73703473_73704908del Heterozygous, de novo	0	.	pLOF o/e = 0.09 (CI 0.04 - 0.2)
P1141	*CHD8*	Chr14:21871660T>A:NM_020920.4:c.2633A>T:p.Q878L Heterozygous, maternal	0	27.8	Missense o/e = 0.56 (CI 0.53 - 0.59)
P1145	*ROCK2*	Chr2:11354970T>A:NM_001321643.2:c.1674A>T:p.L558F Heterozygous, not maternal	0	24.2	Missense o/e = 0.53 (CI 0.49 - 0.58)
P1146	*PIEZO2*	Chr18:10715752C>T:NM_022068.4:c.4978G>A:p.V1660M Heterozygous, maternal	2.31E-05	23.9	Missense o/e = 0.75 (CI 0.71 - 0.78)
P1147	*Chr1q21.1 deletion*	Chr1:145382601_145616000del Heterozygous, inheritance unknown	0	.	.
P1149	*KMT2D*	Chr12:49422934G>A:NM_003482.4: c.14161C>T:p.R4721C Heterozygous, maternal	3.21E-05	33	Missense o/e = 0.81 (CI 0.79 - 0.84)
	CHD7	Chr18:63548074C>T:NM_004361.5:c.2302C>T:p.R768C Heterozygous, not maternal	3.21E-05	34	Missense o/e = 0.77 (CI 0.74 - 0.81)

P1150	*UNC80*	Chr2:210783302G>C:NM_032504.2:c.5060G>C:p.G1687A<br>Heterozygous, not maternal	0	24.3	Missense o/e = 0.63<br>(CI 0.59 - 0.66)

Supplementary Table 3: Detailed interpretation of clinically reportable variants identified by whole genome sequencing.

Case ID	Gene	Variant/Inheritance	Freq (gnomAD or MGRB)	CADD Phred	ACMG classification/ OMIM disorder	Clinical significance
**Individuals with Pathogenic/Likely pathogenic variants considered causative for CP**						
P169	*GRIN2B*	NM_000834.3: c.1739T>A: p.F580Y Het, inheritance unknown	0	29.8	**Likely pathogenic** (PM1, PM2, PP3, PP4)/Epileptic encephalopathy, early infantile, 27 (AD) (MIM 616139)	**Likely pathogenic,** epilepsy with abnormal EEG, on background of extreme prematurity. Novel mutation in *GRIN2B* pore domain
P176	*ARHGAP31*	NM_020754.2: c.1699del: p.P567Rfs*28 Het, maternal	0	.	**Likely pathogenic** (PVS1, PM2)/Adams-Oliver syndrome (AD) (MIM 100300)	**Likely pathogenic,** C-terminal truncation upstream of reported variants[1]. Variable penetrance and expressivity[2,3], and unaffected obligate carriers reported[4]. Early embryonic vascular anomalies and neurological findings reported for *ARHGAP31*[5,6], on background of extreme prematurity
P178#	*MFN2*	NM_001127660.1: c.2220G>A: p.W740* Het, paternal	0	54	**Likely pathogenic** (PS1, PM2, PP3)/Hereditary motor and sensory neuropathy VIA (AD)(MIM 601152)	**Likely pathogenic,** tremor, moderate-severe sensorineural hearing loss. Many pathogenic loss of function variants in ClinVar. Variable age at onset within families and incomplete penetrance reported, also carries VUS in *CAMTA1*
P185	*CLCN2*	NM_001171088.3: c.1598G>A: p.R533Q Het, inheritance unknown	2.00E-04	24	**Likely pathogenic** (PM1, PM2, PP3, PP5)/ Familial hyper-aldosteronism, type II (AD) (MIM 605635)	**Likely pathogenic**, complex phenotype: clinically diagnosed with pseudo-hypoaldosteronism - recurrent bronchiolitis then chest infections, complex partial epilepsy with absence seizures and drop attacks beginning at 15 months.
	CACNA1C	NM_000719.7: c.3568G>T: p.V1190L Het, inheritance unknown	1.22E-05	27.6	**Likely pathogenic** (PM1, PM2, PP2, PP3)/ Long QT syndrome 8 (AD) (MIM 618447),	
P205	*CFTR*	NM_000492.4: c.1520-22del: p.P508del Homozygous, maternal, father unavailable	7.17E-03	21.3	**Pathogenic** (PVS1, PS3, PP4, PM3)/Cystic Fibrosis, classical (AR) (MIM 219700)	**Pathogenic,** diagnosed cystic fibrosis. Reduced fetal movement and fetal distress syndrome, *CLCN1* may contribute to complex phenotype.

Case ID	Gene	Variant/Inheritance	Freq (gnomAD or MGRB)	CADD Phred	ACMG classification/ OMIM disorder	Clinical significance
	CLCN1	NM_000083.3: c.2680C>T: p.R894* Het, not maternal	3.18E-03	35	**Pathogenic** (PVS1, PS3, PP3, PP5, BS1)/ Myotonia congenita (both AR and AD) (MIM 160800)	
P217	*SPAST*	Chr2:32347645_32354557del Compound het, paternal (Supplementary Figure 6)	0	.	**Pathogenic** (PVS1, PM1, PM2)/ Spastic Paraplegia 4 (SPG4) (AD) (MIM 182601)	**Pathogenic,** novel variant removing amino acids 367-415 at the C-terminal end of the AAA domain. Variable age of onset and penetrance within families reported for SPG4[7].
		NM_014946.4: c.131C>T: p.S44L Compound het, maternal	4.54E-03	21.2	**Spastic Paraplegia 4 modifier**	**Modifier,** age of onset/severity HSP[7,8]
P225[#]	*SPAST*	NM_014946.3:c.1625A>G : p.D542G Het, maternal, both siblings share variant	4.13E-04	21.2	**Likely pathogenic** (PM1, PM2, PP3, PP5)/ Spastic Paraplegia 4 (SPG4) (AD) (MIM 182601)	**Likely pathogenic,** Variable age of onset and penetrance within families reported for SPG4[7]. Complex phenotype, carries additional VUS in intolerant genes.
P233	*SYNE2*	NM_015180.4:c.16153C>T: p.Q5385* Het, inheritance unknown	0	44	**Pathogenic** (PVS1, PM2, PP3)/Emery-Dreifuss muscular dystrophy 5 (AD) (MIM 612999)	**Pathogenic,** atypical early presentation.
P236	*TUBB4A*	NM_006087.4: c.1228G>A: p.E410K *De novo*	0	27.5	**Pathogenic** (PM1, PM2, PM6, PP2, PP3, PP5)/ hypomyelinating leukodystrophy, 6 (AD) (MIM 612438)	**Pathogenic,** known pathogenic variant[9]. In keeping with clinical presentation and imaging.
P708	*KLHL3*	NM_001257195.1: c.1446G>A: p.W482* Het, not maternal	0	38	**Pathogenic** (PVS1, PM2, PP3)/ Pseudo-hypoaldosteronism type IID (AD/AR) (MIM 614495)	**Likely pathogenic,** variant likely to escape NMD. Carrier of VUS in *KMT2B*

Case ID	Gene	Variant/Inheritance	Freq (gnomAD or MGRB)	CADD Phred	ACMG classification/ OMIM disorder	Clinical significance
P712[#]	*NF1*	Chr17:28992701_30408700del<br>Het, inheritance unknown	0	.	**Pathogenic** (PVS1, PS3, PM2)/<br>NF1 microdeletion syndrome (AD)	**Pathogenic,** clinical diagnosis neurofibromatosis type 1, on background of premature birth. Cardiovascular complications are a feature of NF1[10].
P715	*TTN*	NM_001267550.2:c.50473C>T:pQ16825*<br>Het, maternal	0	61	**Pathogenic** (PVS1, PM2, PP3)/ Familial hypertrophic cardiomyopathy type 9 (AD) (MIM 613765)	**Likely pathogenic**. Incidental finding for mother, incomplete penetrance reported[11].
P750[+]	*VCX3A, STS, PNPLA4*	ChrX:6451301_8138000del<br>Hemi, inheritance unknown (Supplementary Figure 5)	0	.	**Pathogenic/**Xp22.3 deletion (XLR)	**Pathogenic,** possible atypical presentation without icthyosis. Carries novel compound heterozygous VUS in MARVELD3 (Supplementary Table 6), a component of tight junctions.
P754	*KIDINS220*	NM_020738.2: c.4497del: p.R1499Sfs*9<br>Het, not maternal, Father deceased	0	.	**Likely pathogenic** (PVS1, PM1, PM2) /Spastic paraplegia, intellectual disability, nystagmus, and obesity (AD)(MIM 617296)	**Likely pathogenic,** in keeping with clinical phenotype.
P756	*CACNA1A*	NM_001127222.2:c.7249G>T: p.E2417*<br>Het, not maternal	0	40	**Likely pathogenic** (PVS1, PM2) / Familial hemiplegic migraine 1 (AD) (MIM 141500)	**Likely pathogenic,** variants in CACNA1A reported with recurrent stroke[12,13]. P/LP nonsense and frameshift variants in C-terminus reported in ClinVar.
P759	*SETX*	NM_015046.7: c.5821_5830del: p. A1941Lfs*6<br>Het, not maternal, father unavailable	0	.	**Pathogenic** (PVS1, PM2, PP3) /Spinocerebellar ataxia, with axonal neuropathy 2 (AR) (MIM 606002)/ Amyotrophic lateral sclerosis 4, juvenile (AD) (MIM 602433)	**Likely pathogenic,** lost to follow up.

Case ID	Gene	Variant/Inheritance	Freq (gnomAD or MGRB)	CADD Phred	ACMG classification/ OMIM disorder	Clinical significance
P763	*CPA6*	NM_020361.5: c.799G>A: p.G267R<br>Het, inheritance unknown	2.06E-03	29.1	**Likely pathogenic** (PM2, PM3, PP3, PP2, BS2)/ Familial temporal lobe epilepsy, type 5 (AD/AR) (MIM 614417)	**Likely pathogenic,** diagnosed familial epilepsy. One individual heterozygous for both variants reported[14].
		NM_020361.5: c.619C>G: p.Q207E<br>Het, inheritance unknown	1.43E-03	26.2	**Likely pathogenic** (PM1, PM2, PP3, PP5, BS2)/ Familial temporal lobe epilepsy, type 5 (AD/AR) (MIM 614417)	
	STRADA	NM_001003787.4: c.95-2A>C splicing<br>Het, inheritance unknown	8.23E-06	25.8	**Likely pathogenic** (PVS1, PP3, PP5) / Polyhydramnios, megalencephaly, and symptomatic epilepsy (AR) (MIM 611087)	**Uncertain significance,** oligohydramnios a factor in pregnancy.
P784	*GNB1*	NM_002074.5: c.239T>C: p.I80T<br>Het, inheritance unknown	3.98E-06	25.6	**Likely pathogenic** (PS1, PS3, PM2, PP5)/ Intellectual disability, autosomal dominant 42 (AD) (MIM 616973)	**Pathogenic,** recurrently mutated residue[15], clinical fit: diagnosed global developmental disability, ADHD.
P792	*COL4A1*	NM_001845.4: c.1258G>A: p.G420R<br>Het, inheritance unknown	0	18.3	**Likely pathogenic** (PM1, PM2, PP2, PP3)/ Brain small vessel disease 1 (AD) (MIM 175780)	**Likely pathogenic,** novel mutation altering glycine residue in Triple helix domain.
P910	*TUBA1A*	NM_006009.3: c.50G>A: p.G17D<br>Het, inheritance unknown	0	29.2	**Likely pathogenic** (PM1, PM2, PP2, PP3) / Lissencephaly 3 (AD) (MIM 611603)	**Pathogenic,** clinical fit: bilateral polymicrogyria, microcephaly, spastic/dystonic quadriplegia, epilepsy.

Case ID	Gene	Variant/Inheritance	Freq (gnomAD or MGRB)	CADD Phred	ACMG classification/ OMIM disorder	Clinical significance
P911	*COL4A2*	NM_001846.2: c.3625G>A: p.G1209R Het, paternal	0	24.7	**Likely pathogenic** (PM1, PM2, PP2, PP3)/Brain small vessel disease 2 (AD) (MIM 614483)	**Likely pathogenic,** novel mutation altering glycine residue in Triple helix domain. Term baby with PVL and no recorded risk factors. Variable penetrance and expressivity with asymptomatic carriers reported for *COL4A2*[16].
P931	*ALDH3A2*	NM_000382.3: c.941_943delinsGGGCTAAAAGTACTGTTGGGG: p.A314_P315delinsGAKSTVGA Hom, Inferred IBD	0	.	**Pathogenic** (PVS1, PM2, PM3) /Sjogren-Larsson syndrome (AR) (MIM 270200)	**Pathogenic,** previously described mutation[17]**.** Parents are cousins. Possible atypical presentation, lost to follow-up
P939	*PDGFRB*	NM_002609.3: c.2083C>T: p.R695C Het, maternal	1.13E-04	32	**Likely pathogenic** (PM1, PM2, PP3, PP5)/Basal ganglia calcification, idiopathic, 4 (AD) (MIM 615007)	**Both variants likely pathogenic**[18,19]**.** Possible complex phenotype. Porencephaly, gliosis and PVL on background of preterm birth with IUGR.
	PROC	NM_000312.3: c.226G>A: p.V76M Het, not maternal	4.96E-05	21.2	**Likely Pathogenic** (PM1, PM2, PP2, PP5) /Thrombophilia due to protein C deficiency (AR/AD) (MIM 176860)	
P965	*COL4A1*	NM_001845.4: c.4114G>C: p.G1372R Het, inheritance unknown	0	23.9	**Pathogenic** (PM1, PM2, PP2, PP3)**/**Brain small vessel disease 1 (AD) (MIM 175780)	**Pathogenic**, novel mutation altering glycine residue in Triple helix domain. Term baby with periventricular gliosis, schizencephalic cleft lined by extensive polymicrogyria.

Case ID	Gene	Variant/Inheritance	Freq (gnomAD or MGRB)	CADD Phred	ACMG classification/ OMIM disorder	Clinical significance
P972	*MT-TL1*	NC_012920.1: m.3243A>G Heteroplasmy 58%, Low level detectable in maternal blood-derived DNA	.	.	**Pathogenic** (PS1, PS3)/Mitochondrial myopathy, encephalopathy, lactic acidosis and stroke-like episodes (MELAS) (mitochondrial) (MIM 540000)	**Pathogenic,** known disease-causing variant[20], responsible for 80% of MELAS cases.
P980	*22q11.2 dup*	Chr22:18873001_21469900dup Validated by array (Supplementary Figure 3)	0	.	**Likely pathogenic**/22q11.2 duplication syndrome (AD)	**Pathogenic,** overlapping duplications reported previously in CP[21-23]
P1110	*COL4A2*	NM_001846.2: c.957+2T>C Splicing Het, not maternal	0	25	**Likely pathogenic** (PVS1, PM2)/Brain small vessel disease 2 (AD) (MIM 614483)	**Likely pathogenic,** likely LOF variant in term infant with no reported risk factors, antenatal IVH and hydrocephalus.
P1138	*COL4A2*	NM_001846.2: c.4049G>A: p.G1350D Het, maternal	0	25.6	**Likely pathogenic** (PM2, PM1, PP2, PP3)**/** Brain small vessel disease 2 (AD) (MIM 614483)	**Likely pathogenic,** novel mutation altering glycine residue in Triple helix domain. Term baby with right sided porencephalic cyst. Variable penetrance and expressivity with asymptomatic carriers reported for *COL4A2*[16].
**Individuals with P/LP variants considered risk factors for CP**						
P165	*TRIM32, ASTN2*	Chr9:119311659_119462832del Het, inheritance unknown (Supplementary Figure 2)	0	.	**Likely pathogenic/**recurrent deletion, risk factor for NDDs	**Risk factor for NDDs,** including ASD, ADHD and speech delays[24]
	HTT	NM_002111.8: c.7731G>A: p.W2577* Het, inheritance unknown	0	52	**Pathogenic** (PVS1, PM2, PP3)	**Uncertain clinical significance,** possible risk factor for NDDs

Case ID	Gene	Variant/Inheritance	Freq (gnomAD or MGRB)	CADD Phred	ACMG classification/ OMIM disorder	Clinical significance
P199[#]	*F8*	NM_000132.3: c.5146C>A: p.H1716N Inheritance unconfirmed, father haemophilia A	0	25.5	**Likely pathogenic** (PM1, PM2, PP2, PP3, PP4)/Haemophilia A (XLR) (MIM 306700)	**Risk factor,** female obligate carrier of pathogenic F8 mutation with increased APTT, on background of prematurity and maternal pre-eclampsia.
P214	*15q11-q13 dup*	Chr15:22722801_26749200dup Likely maternal origin determined by methylation (Supplementary Figure 4), inheritance unknown	0	.	**Pathogenic/**15q11-q13 duplication syndrome (AD)	**Risk factor,** recurrent duplications associated with NDDs[25]
P710	*F2*	NM_000506.5:c.*97G>A Het, inheritance unknown	8.44E-03	.	**Likely pathogenic** (PM2, BP7, PP5)/ Venous thrombosis (AD) (MIM 601367)	**Risk factor,** Heterozygosity for F2 c.*97G>A (G20210A, rs1799963) is the second most common genetic risk factor for venous thrombosis[26]. On background of premature birth. Periventricular white matter loss and hyperintensity on MRI noted at 3 years.
P747	*F2*	NM_001311257:c.*97G>A Het, inheritance unknown	8.44E-03	.	**Likely pathogenic** (PM2, BP7, PP5)/ Venous thrombosis (AD) (MIM 601367)	**Risk factor,** Heterozygosity for F2 c.*97G>A (G20210A, rs1799963) is the second most common genetic risk factor for venous thrombosis[26]. On background of extreme prematurity.
P752	*NKX2-6*	NM_001136271.3: c.455dup: p.Q153Afs*207 Het, maternal	3.15E-05	30	**Pathogenic** (PVS1, PP3, PP5) /Conotruncal heart malformations (AR) (MIM 217095), het mutations may predispose to atrial fibrillation	**Risk factor,** predisposing to congenital heart disease and stroke[27,28].

Case ID	Gene	Variant/Inheritance	Freq (gnomAD or MGRB)	CADD Phred	ACMG classification/ OMIM disorder	Clinical significance
P779	*F2*	NM_000506.3: c.598G>A: p.E200K<br>Het, inheritance unknown	1.20E-03	0	**Risk factor**/Thrombophilia (AD) (MIM 188050); Susceptibility to recurrent pregnancy loss (AD) (MIM 614390)	**Risk factor,** on background of gestational hypertension, antepartum haemorrhage, placental abruption, foetal bradycardia and in utero death of twin. Mother 3 previous still births, 3 live births. Severe periventricular haemorrhage in newborn period with post-haemorrhagic hydrocephalus.
P795	*F2*	NM_001311257:c.*97G>A<br>Het, inheritance unknown	8.44E-03	.	**Likely pathogenic** (PM2, BP7, PP5)/ Venous thrombosis (AD) (MIM 601367)	**Risk factor,** Heterozygosity for F2 c.*97G>A (G20210A, rs1799963) is the second most common genetic risk factor for venous thrombosis[26]. On background of extreme prematurity.
P1147	*Chr1q21.1 deletion*	Chr1:145382601_145616000del<br>Het, inheritance unknown (Supplementary Figure 7)	0	.	**Pathogenic**/ Chr1q21.1 microdeletion (AD) (MIM 612474)	**Risk factor.** Event encompassing the recurrently deleted Chr1q21.1 region, reported to predispose to a range of neurological and cardiac phenotypes[29].
**Individuals with P/LP variants of uncertain clinical significance for CP**						
P182	*GALC*	NM_000153.4: c.1592G>A: p.R531H<br>Het, paternal	3.24E-05	29.4	**Pathogenic** (PS1, PS3, PM2, PP3, PP5)/ Krabbe disease (AR) (MIM 245200)	**Uncertain clinical significance,** atypical early signs on imaging and lost to follow-up. Both variants previously reported[30-34], with T112A associated with atypical progression.
		NM_000153.4: c.334A>G: p.T112A<br>Het, not paternal	2.50E-03	23	**Likely pathogenic** (PM1, PM2, PP3, PP5)**/**Krabbe disease (AR) (MIM 245200)	
P188	*COL4A4*	NM_000092.5: c.4720C>T: p.Q1574*<br>Het, inheritance unknown	0	46	**Pathogenic** (PVS1, PM2, PP3)/Alport syndrome (AR/AD) (MIM 203780)	**Uncertain clinical significance,** oligohydramnios and IUGR, lost to follow-up.
P228[#]	*ASTN2*	NM_014010.4:c.2317C>T: p.Q773*<br>Het, paternal	0	46	**Pathogenic** (PVS1, PM2, PP3)/<br>Loss-of-function variants in *ASTN2* are a risk factor for NDDs[31]	**Uncertain significance,** clinical phenotype atypical

Case ID	Gene	Variant/Inheritance	Freq (gnomAD or MGRB)	CADD Phred	ACMG classification/ OMIM disorder	Clinical significance
P232	*EGFR*	NM_005228.3:c.925C>T: p.R309*<br>*De novo*, not shared by 3 siblings	0	38	**Pathogenic** (PVS1, PM2, PM6, PP3)/ variants associated with lung and brain cancers (AD) (MIM 131550)	**Uncertain significance,** novel candidate with role in placentation and development.
P703	*SPG7*	NM_003119.2: c.1045G>A: p.G349S<br>Het, inheritance unknown	8.23E-04	26.7	**Likely pathogenic** (PM1 PM2 PP3 PP5)/Spastic paraplegia 7 (AR)	**Uncertain significance,** no second variant found. Dystonic quadriplegia with deteriorating gait, on background of cord prolapse.
P760	*PNPLA6*	NM_001166114.2: c.3058_3061dup: p.R1021Qfs*38<br>Het, inheritance unknown	1.25E-04	35	**Pathogenic** (PVS1, PM2, PP3)/Spastic paraplegia 39 (AR) (MIM 612020)	**Uncertain significance,** likely *cis* inheritance based on long-read sequencing.
		NM_001166114.2: c.1144del: p.A383Pfs*11<br>Het, inheritance unknown	0	.	**Pathogenic** (PVS1, PM2, PP3)/Spastic paraplegia 39 (AR) (MIM 612020)	
P778	*VWF*	NM_000552.4: c.2561G>A: p.R854Q<br>Het, inheritance unknown	3.47E-03	33	**Pathogenic** (PS1, PS3, PM1, PP5, BS1) /Von Willebrand disease type 2N (AR) (MIM 193400)	**Uncertain significance.** Term baby without other known risk factors.
P1106	*PIEZO2*	NM_022068.2: c.1444del: p. R482Efs*16<br>Het, not maternal, not shared by sibling	0	.	**Likely pathogenic** (PVS1, PM2)/ Distal Arthrogryposis types 3 (AD) (MIM 114300) and 5 (AD) (MIM 108145)	**Uncertain significance,** predicted to be LOF variant. On background of extreme prematurity. Infant with IUGR, restrictive lung disease and vision problems, phenotypes reported in Arthrogryposis type 5 and caused by GOF variants.
P1132	*BUB1B*	NM_001211.5: c.1526C>A: p.S509*<br>Het, maternal	0	36	**Pathogenic** (PVS1, PM2, PP3)/Premature Chromatid separation (AD) (MIM 602860)	**Uncertain significance,** increased risk of aneuploidy in offspring.

Case ID	Gene	Variant/Inheritance	Freq (gnomAD or MGRB)	CADD Phred	ACMG classification/ OMIM disorder	Clinical significance
**Individuals with P/LP variants considered incidental findings for CP.**						
P741	*MITF*	NM_006722.2: c.1018C>T: p.R340C Het, inheritance unknown	0	33	**Likely pathogenic** (PM1, PM2, PM5, PP3)/ Waardenburg syndrome type 2a (AD) (MIM 193510)	**Incidental finding,** possible cause of familial deafness
P746	*ABCA4*	NM_000350.2: c.6316C>T: p.R2106C Het, inheritance unknown	1.31E-04	33	**Pathogenic** (PS1, PM2, PM3, PP5, PP3) /Early onset retinal dystrophy (AR) (MIM 248200)	**Incidental finding,** possible cause of visual problems
		NM_000350.2: c.5282C>G: p.P1761R Het, inheritance unknown	0	26.5	**Likely pathogenic** (PS1, PM3, PP3, PP2, PP5)/Early onset retinal dystrophy (AR) (MIM 248200)	**Incidental finding,** possible cause of visual problems
P749[#]	*GJB2*	NM_004004.5: c.95G>A: p.R32H Het, paternal	3.99E-06	25.3	**Pathogenic** (PS1, PS3, PM2, PP5, PP3) /Deafness, autosomal dominant 3A (AD) (MIM 601544)	**Incidental finding,** possible contributor to deafness
P802	*EFEMP1*	NM_001039348.3: c.1033C>T: p.R345W Het, not maternal	0	25.9	**Pathogenic** (PS1, PM2, PP3, PP5) /Doyne honeycomb retinal dystrophy (AD) (MIM 126600)	**Incidental finding.**

Forty-nine individuals had variants classified as pathogenic or likely pathogenic by ACMG criteria. For twenty-eight of these individuals, the clinically reportable variant was considered likely to be causative for cerebral palsy, while a further 9 individuals carried variants considered to be risk factors for cerebral palsy. Ten individuals carried at least one variant classified as likely pathogenic/pathogenic by ACMG criteria, but with uncertain clinical significance in the individual. A further four individuals carried ACMG pathogenic/likely pathogenic variants which were considered incidental findings likely contributing to other components of the clinical phenotype, but without a link to CP. [#]Additional high impact candidate variants

identified in this individual (see Supplementary Table 4). [+]Additional compound heterozygous variants identified in this individual (see Supplementary Table 6) *premature termination codon. Abbreviations: AD, autosomal dominant; APTT, activated partial thromboplastin time; AR, autosomal recessive; XLR, X-linked recessive; ASD, Autism spectrum disorder; ADHD, Attention-deficit/hyperactivity disorder; CADD phred, combined annotation dependent depletion scaled score; del, deletion; dup, duplication; EEG, electroencephalogram; fs, frameshift; GOF, gain of function; gnomAD, genome aggregation database; hemi, hemizygous; het, heterozygous; HSP, Hereditary spastic paraplegia; IUGR, intrauterine growth restriction; LOF, loss of function; MGRB, Medical Genome Reference Bank; MRI, magnetic resonance imaging; NDD, neurodevelopmental disorders; PVL, periventricular leukomalacia; VUS, variant of uncertain significance.

Supplementary Table 4: Rare, predicted deleterious variants identified in variation intolerant candidate CP genes.

Case ID	Gene	Variant	Inheritance	Population Freq (gnomAD/MGRB)	CADD Phred	pLI/missense Z score (gnomAD)
P163	*KAT6A*	NM_006766.5:c.5231A>G:p.Y1744C	Het, not maternal	0	28	Z = 2.07, o/e = 0.83 (0.78 - 0.87)
P178[#]	*CAMTA1*	NM_015215.2:c.2345C>T: p.S782F	Het, maternal	0	28.7	Z = 3.26, o/e = 0.71 (CI 0.67-0.76)
P180	*SLC2A1*	NM_006516.3: c.767AA>TG: p.K256V	Het, inheritance unknown	1.42E-05	24	Z = 2.93, o/e = 0.53 (CI 0.46 - 0.6)
	SCN1A	NM_001165963.4: c.2522C>T: p.T841M	Het, inheritance unknown	3.19E-05	25.3	Z = 5.22, o/e = 0.55 (CI 0.51 - 0.59)
P183	*SCO1, GAS7*	Chr17:10062326_10600121dup	Het, not maternal	0	.	*GAS7* pLI=0.85, o/e = 0.18 (CI 0.1 - 0.36)
P187	*ROCK2*	Chr2:11104502_11337616dup	Het, inheritance unknown	0	.	pLI=1, o/e = 0.13 (CI 0.08 - 0.21)
P189	*NCOR1*	NM_001190438.1: c.872T>A: p.L291H	Het, not maternal	0	27.6	Z=3.94, o/e = 0.7 (CI 0.66 - 0.74)
P191	*KCNH1*	NM_002238.4: c.376T>G: p.F126V	Het, maternal, sib 1 also carries	0	27.7	Z = 3.82, o/e = 0.55 (CI 0.5 - 0.61)
	ADCY6	NM_015270.5: c.1369G>C: p.A457P	Het, not maternal, sibs do not carry	0	30	Z = 2.68, o/e = 0.73 (CI 0.68 - 0.78)
P193	*CHD7*	NM_001316690.1: c.1226C>T: p.P409L	Het, paternal	0	30	Z = 3.22, o/e = 0.77 (CI 0.74 - 0.81)
	COL5A2	NM_000393.5: c.2792C>A: p.P931H	Het, paternal	0	29.9	Z = 2.44, o/e = 0.77 (CI 0.72 - 0.82)
P199[#]	*NECTIN2*	NM_001042724.2: c.127C>T: p.R43*	Het, inheritance unknown	0	35	pLI=0.97, o/e = 0.12 (CI 0.06 - 0.32)
P201	*TLR7*	NM_016562.4: c.2971C>A: p.L991I	Hemi, inheritance unknown	2.18E-05	25.3	Z = 3.01, o/e = 0.56 (CI 0.5 - 0.63)
	FERMT3	NM_031471.6: c.1717C>T: p.R573*	Het, inheritance unknown	0	42	pLI=0, o/e = 0.35 (CI 0.23 - 0.56)
P211	*ITPR3*	NM_002224.4: c.4978A>T: p.K1660*	Het, inheritance unknown	0	48	pLI=0, o/e = 0.46 (CI 0.37-0.57)
P225[#]	*HERC2*	NM_004667.6: c.14071T>C: p.Y4691H	Het, paternal, siblings do not share	0	24.1	Z=4.42, o/e = 0.76 (0.73 - 0.79)
	FBN1	NM_000138.4:c.7412C>G: p.P2471R	Het, maternal, siblings do not share	2.48E-05	31	Z=5.06, o/e= 0.64 (CI 0.61-0.68)

P228[#]	*THOC7*	NM_025075.2: c.337C>T: p.R113*	Het, paternal	1.20E-05	40	pLI = 0.01, o/e = 0.42 (0.23 - 0.82)
P230	*WNK3*	NM_020922.4:c.3649C>T: p.H1217Y	Hemi, X-linked	4.66E-05	23.8	Z=2.55, o/e = 0.71 (CI 0.66-0.77)
P231	*SUN1*	NM_001130965.3: c.859C>T: p.R287*	Het, maternal	4.01E-06	38	pLI=0, Z= o/e = 0.37 (CI 0.26-0.56)
	TENM2	NM_001122679.1:c.5483G>C: p.R1828T	Het, not maternal	1.62E-05	32	Z=3.3, o/e = 0.77 (CI 0.74-0.81)
P706	*AGAP2*	NM_014770.4: c.2353G>C: p.D785H	Het, inheritance unknown	0	29.3	Z= 3.39, o/e = 0.62 (CI 0.57 - 0.68)
P708	*KMT2B*	NM_014727.3: c.3193G>A: p.E1065K	Het, maternal	0	25.1	.
P712	*FOXP1*	NM_001244813.3: c.923C>T: p.T308M	Het, inheritance unknown	0	27.9	Z=2.28, o/e = 0.67 (0.6 - 0.74)
P714	*STXBP1*	NM_003165.6: c.575G>A: p.R192Q	Het, maternal	3.98E-06	29.5	Z=4.26, o/e = 0.36 (CI 0.31-0.42)
P730	*SRCAP*	NM_006662.3: c.7208G>A: p.R2403Q	Het, not maternal	0	25.7	Z=2.13, o/e = 0.86 (0.83 - 0.9)
P739	*ACTN1*	NM_001102.4: c.2453G>A: p.R818H	Het, maternal	0	33	Z= 3.36, o/e = 0.61 (0.56 - 0.67)
P740	*DNMT1*	NM_001130823.3: c.4381C>T: p.R1461W	Het, paternal	0	33	Z= 4.99, o/e = 0.55 (CI 0.51 - 0.59)
P743	*CHD9*	NM_001308319.2: c.8441T>C: p.L2814P	Het, not maternal	0	24	Z= 3.77, o/e = 0.72 (0.69 - 0.76)
P749[#]	*FGFR1*	Chr8:38270868_38270907del: NM_015850.3: c.*239_*278del	Het, paternal	0	.	pLI= 1, o/e = 0.09 (CI 0.04 - 0.21)
	ATP1A3	NM_001256213.2: c.1420C>T: p.R474C	Het, maternal	4.49E-04	28.9	Z=6.33, o/e = 0.29 (CI 0.26 - 0.33)
P751	*CNKSR2*	NM_001168649.3: c.910A>T: p.I304F	Het, maternal	5.49E-06	26.4	Z=3.61, o/e = 0.47 (CI 0.42 - 0.54)
P775	*ATF4*	NM_001675.4: c.374C>T: p.P125L	Homozygous, IBD	0	22.8	Z=3.54, o/e = 0.7 (CI 0.6 - 0.83)
	NAA15	NM_057175.5: c.1136A>G: p.Y379C	Het, paternal	0	27	Z=3.81, o/e = 0.48 (CI 0.43 - 0.54)
P776	*RAB5C*	NM_004583.4: c.238C>T: p.Q80*	Het, inheritance unknown	0	38	pLI= 0.94, o/e = 0.08 (CI 0.03 - 0.36)
P782	*ARFGAP2*	NM_001242832.2: c.325C>T: p.R109*	Het, maternal	3.98E-06	37	pLI = 0, o/e = 0.32 (CI 0.2 - 0.54)
P785	*DOCK8, KANK1*	Chr9:293723-686363 dup	Inheritance unknown	0	.	DOCK8 pLI = 0, o/e = 0.36 (CI 0.28 - 0.48)
P786	*ITPR1*	NM_001168272.2: c.5048A>G: p.Y1683C	Het, maternal	0	29.3	Z = 5.6, o/e = 0.59 (0.56 - 0.63)

P795	*CAMTA1*	NM_001349613.1: c.1943G>A: p.R648Q	Het, inheritance unknown	4.03E-06	33	Z = 3.26, o/e = 0.71 (0.67 - 0.76)
P796	*PIEZO2*	NM_022068.4: c.2903C>A: p.S968Y	Het, paternal	0	25.3	Z = 3.44, o/e = 0.75 (0.71 - 0.78)
P801	*CDK13*	NM_003718.5: c.664C>T: p.Q222*	Het, maternal	0	35	pLI = 0.91, o/e = 0.2 (CI 0.12 - 0.32)
P902	*CAMTA1*	NM_001349613.1: c.290C>T: p.S97F	Het, not maternal	0	32	Z=3.26, o/e = 0.71 (CI 0.67 - 0.76)
P904	*SCN8A*	NM_014191.4: c.4724C>T: p.A1575V	*De novo*, Het, shared by monozygotic twin	4.01E-06	29.5	Z = 7.64, o/e = 0.35 (CI 0.32 - 0.38)
P913	*ABLIM1*	NM_002313.5:c.244+6delTAAGT splicing	Het, maternal	0	.	pLI=0.93, o/e = 0.19 (CI 0.11 - 0.33)
P918	*USP32*	NM_032582.3: c.1907T>A: p.L636*	Het, not maternal	0	38	pLI=1, o/e = 0.14 (CI 0.09 - 0.22)
P926	*NOTCH3*	NM_000435.3: c.5554G>A: p.A1852T	Het, inheritance unknown	1.59E-05	26.9	Z = 3.53, o/e = 0.74 (CI 0.7 - 0.77)
P934	*NOTCH3*	NM_000435.3: c.5441A>T: p.D1814V	Het, not maternal	0	25.9	Z = 3.53, o/e = 0.74 (CI 0.7 - 0.77)
	KCNA6	NM_002235.4: c.1319C>T: p.S440L	Het, not maternal	0	33	Z = 3.33, o/e = 0.47 (CI 0.41 - 0.54)
P938	*NOTCH1*	NM_017617.5: c.1967T>C: p.L656P	Het, paternal	0	22.8	Z = 3.45, o/e = 0.76 (CI 0.73 - 0.8)
P943	*STRIP1*	NM_001270768.2: c.2175G>A: p.W725*	Het, inheritance unknown	0	44	pLI=0, o/e = 0.37 (CI 0.25 - 0.55)
P944	*TAF1*	NM_004606.3: c.1499G>A: p.R500H	Hemi, inheritance unknown	1.72E-05	29.4	Z= 5.49, o/e = 0.44 (CI 0.4 - 0.48)
P945	*SPTBN2*	NM_006946.4: c.1565C>G: p.A522G	Het, inheritance unknown	0	24.1	Z = 2.63, o/e = 0.81 (CI 0.77 - 0.85)
	PCBP3	Chr21:47131753_47242245del	Het, inheritance unknown	0	.	pLI=0.84, o/e = 0.17 (CI 0.08 - 0.39)
P955	*SPTBN2*	NM_006946.4: c.6146A>G: p.E2049G	Het, inheritance unknown	0	25.9	Z = 2.63, o/e = 0.81 (0.77 - 0.85)
P974	*TRIO*	NM_007118.4: c.6296A>T: p.K2099I	Het, inheritance unknown	0	32	Z= 5.32, o/e = 0.64 (CI 0.61 - 0.68)
	CHD8	NM_001170629.2: c.856C>T: p.R286C	Het, inheritance unknown	2.45E-05	32	Z=5.95, o/e = 0.56 (CI 0.53 - 0.59)
P1114	*AXL*	NM_001278599.2: c.640G>T: p.G214*	Het, inheritance unknown	0	41	pLI=0.01, o/e = 0.27 (CI 0.18 - 0.44)
	PDGFA	NM_002607.6: c.181delA: T61Pfs*3	Het, inheritance unknown	0	.	pLI=0.81, o/e = 0.1 (CI 0.04 - 0.48)
P1123	*SYT14*	Chr1:210056221_210262039dup	Het, maternal	0	.	pLI=0.94, o/e = 0.15 (CI 0.07 - 0.34)

P1129	*THRA*	NM_001190919.2: c.222+1ins GCTTCTTTCGCCGCACAATCCAGAAGA ACCTCCATCCCACCTATTCCTGCAAA Splicing	Het, inheritance unknown	0	.	pLI=0.33, o/e = 0.23 (CI 0.13 - 0.45)
P1134	*HTRA2*	NM_013247.5: c.1115+1G>C Splicing	Het, not paternal	0	32	pLI=0, o/e = 0.4 (CI 0.23 - 0.76)
P1137	*CTNNB1*	Chr3:40831837_41291740dup	Not maternal	0	.	pLI = 1, o/e = 0.03 (CI 0.01 - 0.13)
	PNPLA6	NM_006702.5: c.1810G>A: p.A604T	Het, not maternal	0	33	Z = 4.35, o/e = 0.59 (0.55 - 0.64)
P1140	*CLIP2*	Chr7:73703473_73704908del	Het, de novo	0	.	pLI = 1, o/e = 0.09 (CI 0.04 - 0.2)
P1141	*CHD8*	NM_020920.4: c.2633A>T: p.Q878L	Het, maternal	0	27.8	Z = 5.95, o/e = 0.56 (CI 0.53 - 0.59)
P1145	*ROCK2*	NM_001321643.2: c.1674A>T: p.L558F	Het, not maternal	0	24.2	Z = 4.38, o/e = 0.53 (CI 0.49 - 0.58)
P1146	*PIEZO2*	NM_022068.4: c.4978G>A: p.V1660M	Het, maternal	2.31E-05	23.9	Z=3.44, o/e = 0.75 (CI 0.71 - 0.78)
	ARHGEF3	NM_019555.3: c.1267del: p.Q423Kfs*46	Het, maternal	0	.	pLI = 0, o/e = 0.31 (0.2 - 0.52)
P1149	*KMT2D*	NM_003482.4: c.14161C>T: p.R4721C	Het, maternal	3.21E-05	33	Z = 3.73, o/e = 0.81 (0.79 - 0.84)
P1150	*UNC80*	NM_032504.2: c.5060G>C: p.G1687A	Het, not maternal	0	24.3	Z=5.53, o/e = 0.63 (CI 0.59 - 0.66)

Het, heterozygous; fs, frameshift; dup, duplication; del, deletion; *, premature termination codon; #, additional clinically reportable variant(s) identified in this proband (see Table 1); gnomAD, genome aggregation database; MGRB, Medical Genome Reference Bank; CADD phred, combined annotation dependent depletion scaled score; pLI, predicted loss-of-function intolerance; o/e, ratio of observed to expected number of variants; CI, confidence interval.

Supplementary Table 5: Heterozygous P/LP variants identified in genes causing autosomal recessive Hereditary Spastic Paraplegia.

Case ID	Gene	Variant	Population Freq (gnomAD)	ACMG classification	Clinical description
P703	*SPG7*	NM_003119.4: c.1045G>A: p.G349S<br>Het, inheritance unknown	8.23E-04	**Likely pathogenic** (PM1, PM2, PP3, PP5)<br>Spastic paraplegia 7 (AR)	Dystonic/spastic quadriplegia, on background of cord prolapse; ill at delivery, neonatal seizures. Gait has deteriorated over years.
P760	*PNPLA6*	NM_001166114.2: c.3058_3061dup: p.R1021Qfs*38<br>Het, inheritance unknown	1.25E-04	**Pathogenic** (PVS1, PM2, PP3)/Spastic paraplegia 39 (AR)	Spastic hemiplegia. Father ADHD. Variants likely in *cis* based in long-read sequencing.
		NM_001166114.2: c.1144del: p.A383Pfs*11<br>Het, inheritance unknown	0	**Pathogenic** (PVS1, PM2, PP3)/Spastic paraplegia 39 (AR)	
P761	*SPG11*	NM_001160227.2: c.5794del: p.H1932Mfs*19<br>Het, not paternal	0	**Pathogenic** (PVS1,PM1, PM2, PP3)<br>Spastic paraplegia 11 (AR)	Spastic diplegia, both knees flexed during gait. Dev. delay; visual problems, on background of premature birth.
P913	*SPG7*	NM_003119.4: c.1529C>T: p.A510V<br>Het, not maternal	0.0029	**Likely pathogenic** (PS3, PM1, PP3, BS1)<br>Spastic paraplegia 7 (AR)	Spastic diplegia. Speech delay; ASD (maternal uncle also). Father ADHD. Also carries VUS in *ABLIM1* (maternal)
P962	*SPG7*	NM_003119.4: c.1529C>T: p.A510V<br>Het, inheritance unknown	0.0029	**Likely pathogenic** (PS3, PM1, PP3, BS1)<br>Spastic paraplegia 7 (AR)	Spastic hemiplegia.

Supplementary Table 6: Candidate compound heterozygous and di-genic variants identified in CP cases.

Case ID	Gene	Variant	Inheritance	Population Freq (gnomAD/MGRB)	CADD Phred
P703	**ERCC2**	Chr19:45822388-45863805 del	Het, inheritance unknown	0	.
	**ERCC6**	NM_000124.4: c.631G>C: p.A211P	Het, inheritance unknown	0	24
P750[#]	***MARVELD3***	NM_001017967.4: c.863G>A: p.G288E	Compound het, not maternal	0	26.5
		NM_001017967.4: c.995A>G: p.D332G	Compound het, maternal	0	23.9
P783	***ZFYVE26***	NM_015346.4: c.7032G>T: p.L2344F	Het, inheritance unknown	0	23
		NM_015346.4: c.1505G>A: p.C502Y	Het, inheritance unknown	0	29.5
	RAPGEFL1	NM_001303533.2: c.1195A>C: p.N399H	Hom, likely IBD	0	25.8
P798	***PEX1***	NM_001282677.2: c.1926dup: p.I643Yfs*42	Het, not maternal. **Pathogenic** (PVS1 PM1 PM2 PP3 PP5)	4.85E-04	27.6
	PEX14	NM_004565.3: c.124C>T: p.R42C	Het, not maternal	1.06E-05	26.4

Het, heterozygous; Hom, homozygous; fs, frameshift; dup, duplication; del, deletion; * premature termination codon; [#] additional clinically reportable variant identified in this proband (see Table 1); gnomAD, genome aggregation database; MGRB, Medical Genome Reference Bank; CADD phred, combined annotation dependent depletion scaled score; IBD, identical by descent; pLI, predicted loss-of-function intolerance; o/e, ratio of observed to expected number of variants; CI, confidence interval.

Supplementary Table 7: Approved drugs and clinical trials for genetic diagnoses

Gene	Druggable genome category*	Clinical trials⁺	Other	Clinical utility of diagnosis
ASTN2	Tbio			
GRIN2B	Tclin (multiple drugs)	Clinical trial - L-serine, Multiple clinical trials for epileptic encephalopathies		Y
ARHGAP31	Tbio			
MFN2	Tbio		Candidate drugs[35]	Y
CLCN2	Tclin (Lubiprostone)	Multiple trials for aldosteronism		Y (same individual as *CACNA1C*)
CACNA1C	Tclin (multiple drugs)	Multiple	Simons searchlight data collection	Y (same individual as *CLCN2*)
F8	Tbio			No change
CLCN1	Tbio	Observational studies	Multiple off-label drugs, e.g. Mexiletine[36], Ranolazine[37]	Y (same individual as *CFTR*)
CFTR	Tclin	Multiple	Interaction with *CLCN1* may have bearing on treatment in this child.	Y (same individual as *CLCN1*)
15q11-q13 dup	Tbio (*UBE3A*)	Observational studies	Simons searchlight data collection	
SPAST	Tbio	Multiple clinical trials for Hereditary Spastic Paraplegia	Simons searchlight data collection	Y (2 cases)
SYNE2	Tbio			
TUBB4A	Tclin (inhibitors only, not relevant)		L-dopa may benefit patients with extrapyramidal symptoms[38]	Y
SPG7	Tbio	Multiple clinical trials for Hereditary Spastic Paraplegia		Y
KLHL3	Tbio			
F2	Tclin (multiple)	Multiple clinical trials		Y (4 cases)
NF1	Tbio	Multiple clinical trials		No change
TTN	Tbio	Multiple clinical trials		Y
Xp22.3 del	Tchem (STS)		Approved topical drugs only	
NKX2-6	Tbio			
KIDINS220	Tbio			
CACNA1A	Tchem (multiple, all inhibitors, not relevant)			
SETX	Tbio	Observational studies		
CPA6	Tbio			
GNB1	Tbio			

COL4A1	Tbio	Multiple clinical trials for small vessel disease	4-Sodium phenyl butyric acid has efficacy in mouse models for mutations causing ER stress[39]	Y (2 cases)
COL4A2	Tbio	Multiple clinical trials for small vessel disease	4-Sodium phenyl butyric acid has efficacy in mouse models for mutations causing ER stress[39]	Y (3 cases)
TUBA1A	Tchem (inhibitors only, not relevant)			
ALDH3A2	Tbio	Observational studies	Approved topical treatments only	
PDGFRB	Tclin (multiple inhibitors, topical agonist)			
PROC	Tchem	Multiple		Y
22q11.2 dup	Tclin (*COMT, BCR,* inhibitors)	Observational studies		
MT-TL1	-	Multiple (e.g. arginine, citrulline)	Small studies suggest L-arginine and idebenone may reduce stroke-like episodes[40]	Y
1q21.1 del	-		Simons searchlight data collection	

*Druggable genome categories for genes retrieved from Pharos (https://pharos.nih.gov/)[41], which harvests data from the National Institutes of Health (NIH) Illuminating the Druggable Genome (IDG) program. Categories: Tclin are protein drug targets with approved drugs; Tchem known to bind small molecules with high potency; Tbio limited data and no known small molecules bind; Tdark, do not meet Tclin, Tchem or Tbio criteria. + Clinical trials data retrieved from NIH Clinical Trials Database (https://clinicaltrials.gov), EU Clinical trials register (https://www.clinicaltrialsregister.eu/) and National Organization for Rare Disorders (https://rarediseases.org/).

Supplementary Table 8: Primers for validation of SNVs and indels by Sanger sequencing

Sample	Target gene	Primer name	Primer sequence (5'-3')	Primer name	Primer sequence (5'-3')
P162	*ARHGAP32*	752F	CCAGAACAACCGAAGACCAT	753R	CTGGAAGGTGGATGTTTGCT
P163	*KAT6A*	756F	AGGAAGTCACAGCAGGAGAA	757R	CCACAACCACAACCAGCAC
P165	*HTT*	1072F	CACGGACAGGTGCTCACTTA	1073R	GGTGAGCATGCCAGTCTTCT
P169	*GRIN2B*	1078F	TAAAGAGACGCCAAGCTGGT	1079R	CCTTCCTCAGAGCCATTCAG
P176	*ARHGAP31*	1332F	CCCTCCATCCTTCTGAAACA	1333R	GGGGTTTTCTTCTCCTCCAG
P178	*MFN2*	726F	CCCATCTGTGAGACAGGGATA	727R	TAAGTAGTGCTGGAGGGCTCA
P178	*CAMTA1*	1502F	GAGGTCCTGCTCAAGTCTG	1503R	ACATCTCTGCCTGGGTGAAG
P180	*SKI*	1436F	CCAGGGCACATTGTCAGATAGA	1437R	CTCCTTCACCACCTTCTCCAG
P180	*SLC2A1*	1438F	CAGACAGCTGCTGGGACAG	1439R	ACTCTGAGCCACCCTCACC
P180	*SCN1A*	1094F	GCTAATGGTTGTGTGGCAAA	1095R	ATCAAGCAAGCCATTTCACC
P182	*GALC*	1496F	GGGTATTTCAGCCAGATCCA	1497R	AACAGAGAATTTCTTACATTCTGAACA
P182	*GALC*	1498F	TGGGATTCCTCTTCTTAGCTTC	1499R	ATGATTTCCCCAACTTCAGG
P185	*CACNA1C*	1100F	CACATCCCACACTTCTCTGC	1101R	AGCAGGATGAGGACGAACAT
P185	*CLCN2*	1532F	CAAGGAGACTGGTCCTGAGC	1533R	GAGCACCCCTTGGTCCTTAG
P188	*COL4A4*	766F	TGAATACCCGATCCAGAGGC	777R	TGCCACTATGCCCAGAGAAA
P189	*NCOR1*	1294F	GCAGTCCCACCTACTCAGGA	1295R	TGCTCCATAAGGTCACCACTT
P191	*ADCY6*	768F	ATTCCCCTTCCCAGTGACAG	769R	TTCCCTCCAATTCCTTCCCA
P191	*KCNH1*	1246F	TGCCTTGACAACCTTAAATGC	1247R	GGGACACCTGTGTGGTTCTT
P193	*COL5A2*	1504F	AACTTACAGGTTGCCCATCTTCT	1505R	TATTCATCATTGGAGTGGAGCTT
P193	*CHD7*	772F	CATCCTTCACAGCCTCAGGG	773R	TCCTCTGTCCGATTCCCATG
P199	*F8*	1108F	TCACAAGAGCAGAGCAAAGG	1109R	GGCAAAGCAAGGTAGGACTG
P199	*NECTIN2*	1106F	TCCCTCACCTTCTCCACCTA	1107R	GACTCTGAGCCAGGTCATCC
P201	*FERMT3*	1112F	AAATGGCTTGCTCAGGGTTA	1113R	CAAATGGTGGGTGGGATAAG
P201	*TLR7*	1110F	CCTTTCCCAGAGCATACAGC	1111R	ACCGTTTCCTTGAACACCTG
P205	*CLCN1*	1442F	TTCAGCTACAGAAGGCCATTGA	1443R	CCACGGTCTTTATGAGGAGGTC
P211	*IL1RAP*	776F	CCCCAGAACTTCAGAGCTCA	777R	AGATAGTAGCCACCACCCAC
P211	*ITPR3*	956F	TGTCCAAGTGAGCGAGACAC	957R	TGCCCACTCTTCTCACCTCT
P217	*SPAST*	1144F	AATGAATTCTCCGGGTGGAC	1145R	GAAGATGAGGCTCCGACCTA
P217	*SPAST*	1056F	CTACCACTCATAAGGGTACTCCG	1059R	TCCAGCAGACTGTACACCATC
P220	*TNR*	1114F	ACTCGCTGTCACAGATGCAC	1115R	ATCCTTCCCCAGCACCAT
P225	*FBN1*	1336F	CAATTCTACTTTACCTTTGCAGCTC	1337R	CTAAAATTCTTCGTTAGACCCTGTG
P225	*SPAST*	1172F	CCAGGAGGTGGAGATCACAG	1173R	TGGACTTCTTAAACTTCTAAGGTGGT
P225	*HERC2*	786F	AAGCGAAGGAAAAGAGAGCG	787R	GCCCTGTGTGTTGACTGAAG
P225	*ICAM5*	788F2	GCAAGAAGGGCGAGTACAAC	789R2	GACGCCCTTGGAGTTGAATA
P226	*SRGAP1*	1248F	CACTTTCTTTGGAGTTTGCTGA	1249R	TCTCATAAACAGGGCCATCC
P228	*ASTN2*	1506F	AGGAACCTCCTCTCAAAGATGAC	1507R	TAGTCCTCAGCAGTCTCCACTTC
P229	*BCOR*	1118F	GGACTGCTGAGGTAGCAAGG	1119R	CAGCAAAGGGGTTTGGATAA
P230	*WNK3*	1340F	TGTGACCTCAGCATCTTGTGA	1341R	CTGTCCAATGTGGCTGCAAG
P231	*TENM2*	1146F	ATGTCCTAGCGGGCACCAT	1147R	AGGCTTTGAAGCTGGGAAAC
P231	*CDNF*	1026F	CTGGTTGGAAGCAGCAGAAT	1027R	CCCTTCCTATCCCTTCCTTG
P231	*SUN1*	1444F	ATTCCCACAGCACCAGAGAG	1445R	CAGGTACACCACGCTTCTCA
P232	*EGFR*	722F	CCCTCAAGAGGACCTGGAC	723R	CTCAGCAGCCGAGAACAAG
P233	*SYNE2*	1500F	TGTTATTTGCGTTGCACTGA	1501R	GATGTTTGCGAGCTGTTGAA
P235	*BCOR*	1034F	GGCAACAGGAGAGCTGTGTC	1035R	TTCTCCAGTCTGCACCAATG
P235	*WDR47*	1028F	TCCAGGATTACGAGGAGTGC	1029R	AGAGCGAGACCCTGACTCAA
P236	*TUBB4A*	994F	AGGTATTGTTAGGGTCAGCGG	696F	TGAGCGTGCAGAGCAAGAACAG
P701	*KCTD17*	1036F	TGCCCTCTATGTGACCCTTC	1037R	GGAGACTGTGGTGGGGAAC
P703	*SPG7*	1342F	CAGAACGCTTCCTCCAGCTT	1343R	CCCAACCACAGCACCTACCT
P704	*CACNA1S*	1043F	GGTGCTCTGAGGCGATAGAC	1043R	GCCTGGAGTTTGTCTGAAGG
P706	*AGAP2*	732F2	GCAACTATACCAGCGCAACC	733R2	GAAAGCCCGAAATTCCCAGA
P708	*KLHL3*	800F	GCCCAAGTTAAGCAAGCTCA	801R	TCTCCCATGGCTTTTGTTTGA
P708	*KMT2B*	802F	CCTGGCTGAGCTCAAATCCT	803R	GCCTTGTCCCTCCTACACAA
P711	*DIP2A*	840F	TAGAGGCTCCTGTGCTGTTG	845R	TTTGTCCCCTTGAGCCTTTG
P712	*FOXP1*	1148F	TGGGCACGTTGTATTTGTCT	1149R	GGGTTGCTCTGGTTTTCTGA

P714	*STXBP1*	1508F	ATTCCTTGGACTCTGCTGACTCTT	1509R	CCTCTCTCTCCTCATGAGCCTATC
P715	*TTN*	1510F	CTCTCATCCATTTCTCAGTGCCTA	1511R	GCAGACCTAAAATGGACAGTTCCT
P720	*EML2*	1154F	TGGGGGATCTTGCTACCTC	1155R	AGGTCCTCATTCAGAATCTCC
P720	*PIH1D3*	1156F	CCTGAAGACCTGCAAGCACT	1157R	GTGAAACATAGCAACCACGA
P721	*MATN3*	1346F	ATGTCCAGAGTGTCGATTATCC	1347R	CTTGGACCTGGTGTTTATCATT
P721	*GALC*	1446F	CCCATGTAATGGGTCTCTGG	1447R	GGAGAGCTTCTGAAGGATTTGA
P730	*SRCAP*	842F	GCAAAGACCTGGACCAAGCC	843R	TGGGGCTGAAATGGGTACTG
P730	*CNTN6*	844F	CCTTGTGGTTGTGGTTGATAGT	845R	GAGGTGTGGGAATGAAATGAACA
P737	*KAZALD1*	850F	TGGGAATGCGCCAACCTC	851R	TGGAGCTTTCACGTGGTTCA
P739	*ACTN1*	1160F	ATCACAGTCCAGCCAGTGC	1161R	CCCAGGGTCTTCAGTCCATA
P739	*PROC*	716F	AGACCATAGTGCCCATCTGC	717R	GGGCTTCCTTGTCTCTGATG
P740	*DNMT1*	1348F	CTTACAGGACCCACCTTCCA	1349R	CAGGACATGAGTGCATTGGT
P741	*MITF*	852F	AACCAAGCACCACCTGTTCC	853R	CAGACAGTCAACCTCAGGCA
P743	*CHD9*	854F	CTGGGTTGATGGGAATGCCT	855R	CTGAACCCTCATCTGTGCTGT
P747	*ARHGAP32*	1166F	TGATTTACCATTGCGCTAAGA	1167R	GGAAGGCTTTCATAGAATTGGA
P748	*CCDC88C*	1170F	CTCTGATGTGGGAGGCATCT	1171R	CCCTCATCTCAATCCTGGAA
P748	*LBD3*	1168F	TGTTCCCTGGTAGGATGCTC	1169R	GGCAGGAGTTAGTTCCCTGT
P749	*GJB2*	1196F	TGGGTTTTGATCTCCTCGAT	1197R	ACACGTTCAAGAGGGTTTGG
P749	*ATP1A3*	1350F	GGCGTCATAAGGAACCTGAG	1351R	CAGCTGTCCCTGTTCTGACA
P750	*MARVELD3*	864F	CGGATTCAGTGCCAGCTTTT	865R	GCCTTTGTAGCGCTTCTGTT
P750	*MARVELD3*	866F	AGGAGCCAAGAGTCGAACAA	867R	TGTAACTTCCGGGCTGTTCT
P751	*CNKSR2*	1352F	TGTGTGTTGTTTGTGTGCGTA	1353R	GCTGCCTAGGACAACGTGAT
P752	*NKX2-6*	1520F	TGTAGCCTCCGTAGCAAGAGTAG	1521R	TGTCACAATTTATGTTCCAGCAG
P753	*CNTNAP2*	1204F	GCAGTGTCATCTCCTACCACAG	1205R	GAGCAATCGCAGGAGTAACC
P753	*DOCK6*	1206F	CCTCCTCCTGCTCATTATGC	1207R	CCTGTGCCTGAGGCTCCTAC
P754	*KIDINS220*	734F	GGAGACTTCGGCACTCTCTCT	735R	GTTCATCAGGGGGCTCTATTC
P756	*CACNA1A*	968F	GGCTTAGCACCAATCATCGT	969R	ACGTGTCCTATTCCCCTGTG
P759	*SETX*	1448F	ATCAGTGCCCTCACCCATAC	1449R	GGTCACTTTGGTGTCCTTGTC
P760	*PNPLA6*	1354F	CTCTTTCATCGGAGCGTTGT	1355R	GGACAGCTGGGGATCATAGA
P760	*PNPLA6*	1386F	GGGCTCCAAGAGAATGGTC	1387R	GGCTGGACAAGGTCAGGTT
P761	*SPG11*	930F	TCCCTTCCTTCTTGGAGACA	931R	GCCTACTGGATGATGGCTGT
P763	*CPA6*	1356F	CCCAGTTGGTCCCAAACTTA	1357R	CCCTGGGCTTGTCTATTCAC
P763	*CPA6*	1358F	CACTGACTAGCTGAAATGGACA	1359R	TTGGTTACTGTGAATGCTCTTG
P763	*STRADA*	1194F	GGGTGAAACCTGAATCTCCA	1195R	GCCCTATCTCTCTGCAGCAT
P763	*REM2*	1138F	ACCTCCACAGACCCACCATA	1339R	CTTTCTTGGTGAGGCTCTCG
P769	*CCDC88C*	972F	GCTCAACCACGAGACAGTCA	973R	CAAACAGGAGTCCCCTCAGA
P772	*COL6A3*	984F	GCTCACTGGCGAAGGTGTAT	985R	CCTCTGAGTCCGTGGACAAT
P772	*PDGFRB*	1450F	GGTGTCTGACTTCTGCACCA	1451R	CCTGACCCTTCTCCCTATCC
P773	*ZFHX3*	974F	GCTGAGATCCGTGTCAGACTC	975R	TGAACCTGCAAGAGATGGTG
P775	*ATF4*	738F	TAGGGGCCTCCTACCTTTGTA	739R	GGGTGTCTTCCTCCTTTATGC
P775	*NAA15*	740F	TCCTTTCATAGTACCTCCCTTGTT	741R	CACCAAATTAGCATGACCACA
P776	*RAB5C*	834F	GGCTCTGATAACCCAATCCCT	835R	ATCTCAGTGGTGGGCTTCTG
P778	*VWF*	1432F	CTCACCTGCACCAGAACGTA	1433R	TGTTCCTTCATTGCCTCCAT
P780	*NECAP1*	1360F	CTGGAAATTAGACCAGCCTG	1361R	TACTGGTGCCTGAGCAAAGA
P782	*ARFGAP2*	1252F	CCCCAAGAACAGGCACTTAC	1253R	TGTCTCCTACAGCCAACACG
P782	*AHDC1*	1518F	TCAAAATACCTTTGCCTGAAGAT	1519R	CTCCTTAGTGACTTGACCCTGTC
P783	*RAPGEFL1*	1302F	AATGGACCCTGTCTTTGTGC	1303R	GGGAGGGACCTGACTACTCC
P783	*ZFYVE26*	1002F	GGTCAGGTGCTGATTGTCCT	1003R	GGCTCCTCAACACACCAAGT
P783	*ZFYVE26*	1004F	GGATGGAGGGAAGTGCTGTA	1005R	AGGGTGAGGCAAGAGAGACA
P784	*GNB1*	1066F	TCCAGTCCCTACCTTGTTGG	1067R	TGGCTTTGTCTGAAGCACAT
P786	*BSCL2*	1366F	GGAAGACAGACACCCAGAGC	1367R	AGAGCAGCTAGGAACGCAAG
P786	*ELOVL4*	1254F	GATTTGCTCACACCATTTGC	1255R	TTACACTGACTGCCCCTTCC
P786	*ITPR1*	832F	TGCCCTGAAGTATCTTTAACCTG	833R	CAATCAGCCTATAAGAGACGACT
P789	*CHRNB2*	836F	TCAACCTCATCATCCCCTGTG	837R	GCATGTGGTCTGCGATGAAG
P790	*IKBKG*	830F	CTGGGTTAGGGCTCACTGTG	831R	GCCACTCATGTAAGACCAGGA
P792	*COL4A1*	1528F	GATGCCAGGAGTCTCAGAGG	1529R	TCCCTGGTGAAAGAGGAGAA
P795	*CAMTA1*	1216F	TGTCGCTTGTTCTTGTTTCC	1217R	AGCCCAGGACAAGTCTTTCA

P796	*PIEZO2*	824F	GCTAAAGGGCAGTCAGGAAT	825R	ACTTTAGTACCCCAGCACCT
P798	*PEX1*	1388F	TGAACTCCTTGAGCAGAAACAA	1389R	GCGATACCATACATACTGCACCT
P798	*PEX14*	1390F	GCCAGTGAAGACAGGGAAAC	1391R	ATGAGTGGGCAGCTCTAGGA
P799	*GNAO1*	1452F	ACACCTTGCCTGTTTGCTCT	1453R	TCCGTCACAGCATCAAAGAC
P801	*CDK13*	820F	TGGTGGAATACGAGGATGTGA	821R	TTAGGCTCGGTCTTGTCCTC
P802	*EFEMP1*	1222F	CACATGGCATTTGAGACTGG	1223R	TGCTCTCACACCTCCTTCCT
P802	*PCBP3*	1078F	TTTCTCACTGCACGACGCCTG	1079R	CCTGTCTCGCACTTTGAGCTGT
P902	*CAMTA1*	718F	ACAGTGTGCTTCTGGGACTG	719R	AGGATGTGCGAGGCTGAC
P904	*SCN8A*	789F	CATGGTGACAATGATGGTGGAGAC	790R	CTCATCCTCACAATCAGTCAGGTC
P905	*DST*	814F	TCCTTGATTGCTGTTGTCACA	815R	ACTCTCTCTGTACTCACTCTGTC
P907	*TBX2*	812F	GAGTGTCCCTGATCCTGCTC	813R	CGGCCCAAAGAACTGCTG
P910	*TUBA1A*	712F	GAATCATCTCCTCCCCCAAT	713R	TTTGTATCGTGCTGGGGATA
P911	*COL4A2*	748F	GTGCTGTCCCACACATGAAA	749R	AGCAGCGTACAGGACCACAG
P913	*SLC9A5*	1126F	GAGAGAGTCCCCGACTACGA	1127R	CCAAACCTAAGTCCCAGCAC
P913	*SPG7*	1524F	CTGTGGCAGTAACTAGGCTTGAG	1525R	TCACTGTGTGGAAGCAACATAAC
P918	*USP32*	952F	GTCCATGGTGATCAATTGCTATT	953R	CGCACTCTAGTCTGGCAAC
P918	*KCNH5*	1268F	AACACCACCATCTTGGGAAG	1269R	GGCAAAATACAAAGGTCACACA
P918	*DEAF1*	1266F	GCTTCCCAAAGTGCTGGTAT	1267R	TACTATGCTTCCCGGTGTCC
P926	*NOTCH3*	1226F	CCAGCTCTGAGGTCCAAAGT	1227R	GCCAACTGAAGAGGATGAGG
P931	*ALDH3A2*	744F	GCTGTGAACAGGGTTCAATGT	745R	AAAGAGCCAGAGGCTTTTCAC
P934	*NOTCH3*	1304F	TCTTCCACCATGCCCTCTAC	1305R	CCTTGTCCTGTGTTCCCTGT
P934	*KCNA6*	1124F	AGAGGCTGACGATGACGATT	1125R	GTCAGACCTCCGTGAGCATT
P936	*FGFR2*	1090F	TTGTTGATATCTCTGGCGAGTCCAAAG	1091R	CTGGTATTACAGGCATGAGCCACC
P938	*NOTCH1*	1122F	GCTCTGACCGGAGACAAGAG	1123R	CAACGCCTACCTCTGCTTCT
P939	*PROC*	1368F	AGCGGGAGTGCATAGAGGAG	1369R	CTCCTAAGAGGGCCTCAGCA
P939	*PDGFRB*	1370F	TGCTCCTCAGGTATCCCAAA	1371R	CACTGAGTACTGCCGCTACG
P943	*STRIP1*	1372F	GACTTCCAGGCAGAGGAGTG	1373R	AATGGGCTTGGAGAAGACCT
P944	*TAF1*	1232F	GTGCTGAGCCTGCAACATTA	1233R	GAAGAAACACTGGCCTCTGC
P945	*SPTBN2*	1234F	CCCTCGACTCTTGATCACTCTT	1235R	AAGCCATTGAGACGGACATC
P953	*EMC1*	808F	ACCCATACTCACAGCCTTCA	809R	TGACCCCAAAGCCTGTTTTC
P955	*KMT2E*	806F	CCCAAGCCTCCATAATTGACG	807R	GAACGTGTGTCTGAGGAGGA
P955	*SPTBN2*	804F	AGAAGCCAGCACAGGTCAG	805R	TCTGACCCACCATCTTCCTG
P957	*MFN2*	1434F	GGTGGATGTGGCCTGAAG	1435R	GCCTCCGCATCTGATCTTT
P962	*SPG7*	1374F	CGCACCTGTGGCAGTAACTA	1375R	CGGGTTGAGAACGTACCACT
P965	*COL4A1*	1284F	TTCCTGGGTTTTCTTCTGGA	1285R	ATAGCTTCACCCGGGATTCT
P972	*MT-TL1*	1376F	GTGATCTGAGTTCAGACCGG	1377R	CGATTAGAATGGGTACAATGAGG
P974	*TRIO*	716F	TGAACAGCCCATACGATCTTT	717R	TCATTCTGAACTCGATGTAAATAAAAA
P983	*RASGEF1B*	1312F	TTTCCCCTCCAATTCTACCC	1313R	GCAAAGCGTGTTTGACACAT
P1105	*WNK4*	1236F	GCACTCTTCCCTTCTCATGG	1237R	CAGAGCTTCCCTGGTCTCTG
P1106	*PIEZO2*	872F	ATAGCTATGAGGGCACAGATGT	873R	AGGGCTTTATGAATGGGAAACA
P1110	*COL4A2*	870F	TCAATCTGTCCATCGGCATTT	871R	TGAAAGGGAGGAAGCAAAAGA
P1114	*AXL*	978F	TTCAACTCCTGCCTTCTCGT	979R	CCTCAAGTGATCCACCTGCT
P1114	*PDGFA*	878F	GATGCCCACACTGCGAATTG	879R	GGTGGGAATCGAGGTTCTCC
P1129	*THRA*	1422F	TCACCTCGTGTCTCTCTAGGAT	1423R	GTGGAGGTGCATACCTGTAATC
P1130	*EXD1*	1190F	TGTTTAGGTAACCATCCACCAG	1191R	TCTTGATGTCTTGACCTTGTGA
P1132	*BUB1B*	880F	TGCTGATGAGGGAGGACAGA	881R	ATTGACGGTTAGAAACAGAGGT
P1132	*FIGN*	1238F	CGGTTCTCATCCGACTGACT	1239R	GACCTGGTGAAGGCTGTCAT
P1134	*HTRA2*	1378F	TTCCTGGAGGGTGGTCTACT	1379R	GAAGGGAGACAGCTCTTGTG
P1136	*FARS2*	1240F	GCTGATCAAGGAGAGGGTGA	1241R	TGGTCACGCCTGTAGACATC
P1138	*COL4A2*	902F	TGGAAGCAAAGGTGACACAG	903R	TTGGAGCTTCCCAGACAACT
P1141	*CHD8*	908F	ACCAAAACAGCAGGCTAGGA	909R	TGGTTCACTGGATCTCAACA
P1145	*ROCK2*	910F	GGAGCTGTTGTTTGTGAGCA	911R	CCCAGGCAGAAAGTTCAAAA
P1146	*PIEZO2*	1244F	GTCAGCATGCATCGTTCAAT	1245R	GGTAAAACATCGCTGGGCTA
P1149	*KMT2D*	1380F	CCCACATCCAGAGTAGCACA	1381R	AGCGATTCTCCTGACAGCAT
P1149	*CHD7*	1242F	CCTGGTGCTCCTCCTTATGA	1243R	CAACCATCTGTCTCCAGCAA
P1150	*UNC80*	1132F	CTGAGGCCCAGATAGGTTCA	1133R	ACCTTTGGCCAAGAAATCAA

Supplementary Table 9: Primer sequences for qPCR copy number variant validation:

Sample	Targeted gene	Primer sequences
P165	ASTN2	Forward: 5’-CAAACCATGAACACTCCAACCC-3’<br>Reverse: 5’-TACGTTGACAGTGACCTAAGGC-3’
	TRIM32	Forward: 5’-TGATGAGGTTCCTGAGGGTTTG-3’<br>Reverse: 5’-AGTAAGGCAGTTGCTCTTTGGC-3’
	PAPPA1	Forward: 5’-GTTCTCTTTTGTCCCCAATGCC-3’<br>Reverse: 5’-ATGTACTGGAAAGAGGCACAGG-3’
P183	SCO1	Forward: 5’- CGATAGCAGCAGAACAAACAGG-3'<br>Reverse: 5’-AGGTGACGTCTTCCAGATGTTC-3'
P187	ROCK2	Forward: 5’-AGGGAAGCTGAAGCCTTATTCC-3'<br>Reverse: 5’-GCTTCTGAGTCTGCATTGACAC-3'
P785	DOCK8	Forward: 5’-CGAGGACTTTGAGAAGCAGAAC-3'<br>Reverse: 5’-TGATGGGTAAAGGGCAAAGAGC-3'
P1123	SYT14	Forward: 5’-GACCACAACATCCTTTGACACC-3'<br>Reverse: 5’-TGTTGAACCACCCAGTTGTCAG-3'
P1137	CTNNB1	Forward: 5’-TCTGTGGCAGCAGCGTTG-3'<br>Reverse: 5’-TGAAGCTGCTCCTCAGACCTTC-3'
P1140	CLIP2	Forward: 5’-AGGAACTTAGACCTGCGTTGAG-3'<br>Reverse: 5’-AAGCCAGAATCCCATCATTCCC-3'
P945	PCBP3	Forward: 5’-GATAGATCTGGAGAGCTGTTGC-3'<br>Reverse: 5’-GCGCACACAGAGGAAATTCTTC-3'
P947	NIPA1	Forward: 5’-TTAGCCAGGTGTGGTGTTG<br>Reverse:5’-TCATTGCAAGCTCCACCTTC
P1130	EXD1	Forward: 5'-TGGGCTGTACTTGAGGTCATTC-3'<br>Reverse: 5’-ATGTATGTGAGCCTGCTTCTCC-3'

Supplementary Table 10: Probe sequences for MLPA validation of CNVs:

Sample	Target gene	Probe sequences
P785	*KANK1*	L:5’-GGGTTCCCTAAGGGTTGGAcCTGGATCTCGAAAAGATTTTCACAAGACG-3’<br>R:5’-CTTCCGAAACAGACGTTTGAGTCGGacTCTAGATTGGATCTTGCTGGCAC-3'
P1130	*EXD1*	L:5’-GGGTTCCCTAAGGGTTGGACTTACCTAACAGAAGATGCTTTTGCTCTCA CTGAG-3'<br>R:5’CCTTGTTCCACTTCATCTAGTAGTTCCACTGTAATCTAGATTGGATCT TGCTGGCAC-3’
P1137	*CTNNB1*	L:5’-GGGTTCCCTAAGGGTTGGAcgctactaGTGTGTGCTGATCTAGAGCAGAC TGCTG-3'<br>R:5’-ATCCTCCTGGGTCCCACTTACCCAaactaaatctacTCTAGATTGGATCTTGC TGGCAC3’
P165	*ASTN2*	L:5’-GGGTTCCCTAAGGGTTGGAcgctactacCTGAGGCACGAGCAACTCGGATAG-3'<br>R:5’-TACCCTGTGAGTCAGGGCAGCTCTGggtcaaactaaatctacTCTAGATTGGATCTTGCTG GCAC-3'
P187	*ROCK2*	L:5’-GGGTTCCCTAAGGGTTGGAcgctactactattagCAAGGGCGCAATCAGTCAATGGGACT-3'<br>R:5’-GTTTGAGCCAAGAATCAAATGGAAGTTCCAACtaaatctacTCTAGATTGGATCTTGCTGG CAC-3'
	MRC-Holland Control Probe D	L:5’- GGGTTCCCTAAGGGTTGGAcgctactactattCGCGTGTGTCCCGACTCTCAC TCAAT-3'<br>R:5’-TTATAGGGGAGAGGGACTCGCCAAATCCCTGaaatctacTCTAGATTGGATCTTGCTGG CAC-3'

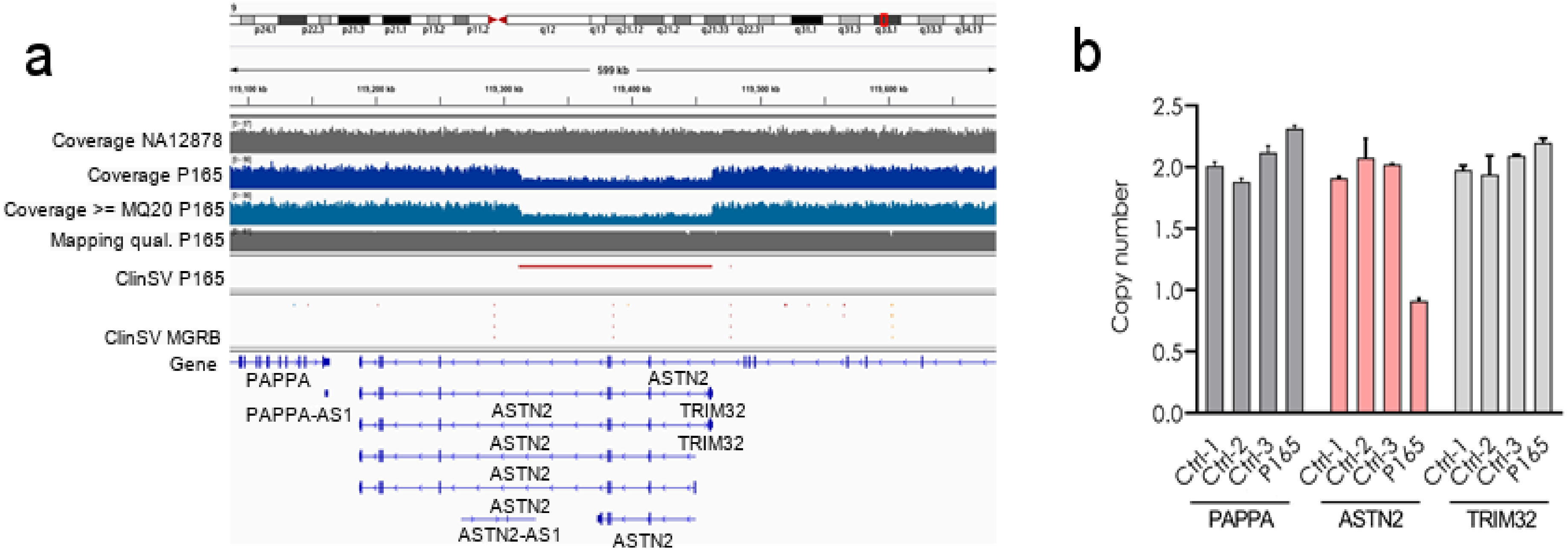
a
599 kb
Coverage NA12878
Coverage P165
Coverage >= MQ20 P165
Mapping qual. P165
ClinSV P165
ClinSV MGRB
Gene
PAPPA
PAPPA-AS1
ASTN2
TRIM32
ASTN2-AS1
b
Copy number
2.5
2.0
1.5
1.0
0.5
0.0
Ctrl-1
Ctrl-2
Ctrl-3
P165
PAPPA
ASTN2
TRIM32

Supplementary Figure 2: Validation of *ASTN2/TRIM32* deletion in sample P165. a) Integrative Genomics Viewer image of the ClinSV call for P165 (deletion Chr9:119311659-119462832). This deletion is predicted to result in deletion of multiple exons for all *ASTN2* transcripts, as well as deletion of exon 1 and 2 of *TRIM32*. No copy number variants were detected in this region for any samples from the Medical Genomes Research Biobank cohort (ClinSV MGRB). **b)** Validation of the deletion by qPCR (see Supplementary Methods, error bars are SEM). Ctrl1-3 are gDNA samples from unrelated individuals from the DNA biobank. No loss of copy number was observed for upstream gene *PAPPA* (primers targeting Chr9:119116950-119117069) or the region of *TRIM32* downstream of the expected deletion (primers targeting Chr9:119463359-119463450). Primers targeting a region within *ASTN2* (Chr9:119458018-119458115) show a loss of copy number in P165.

a

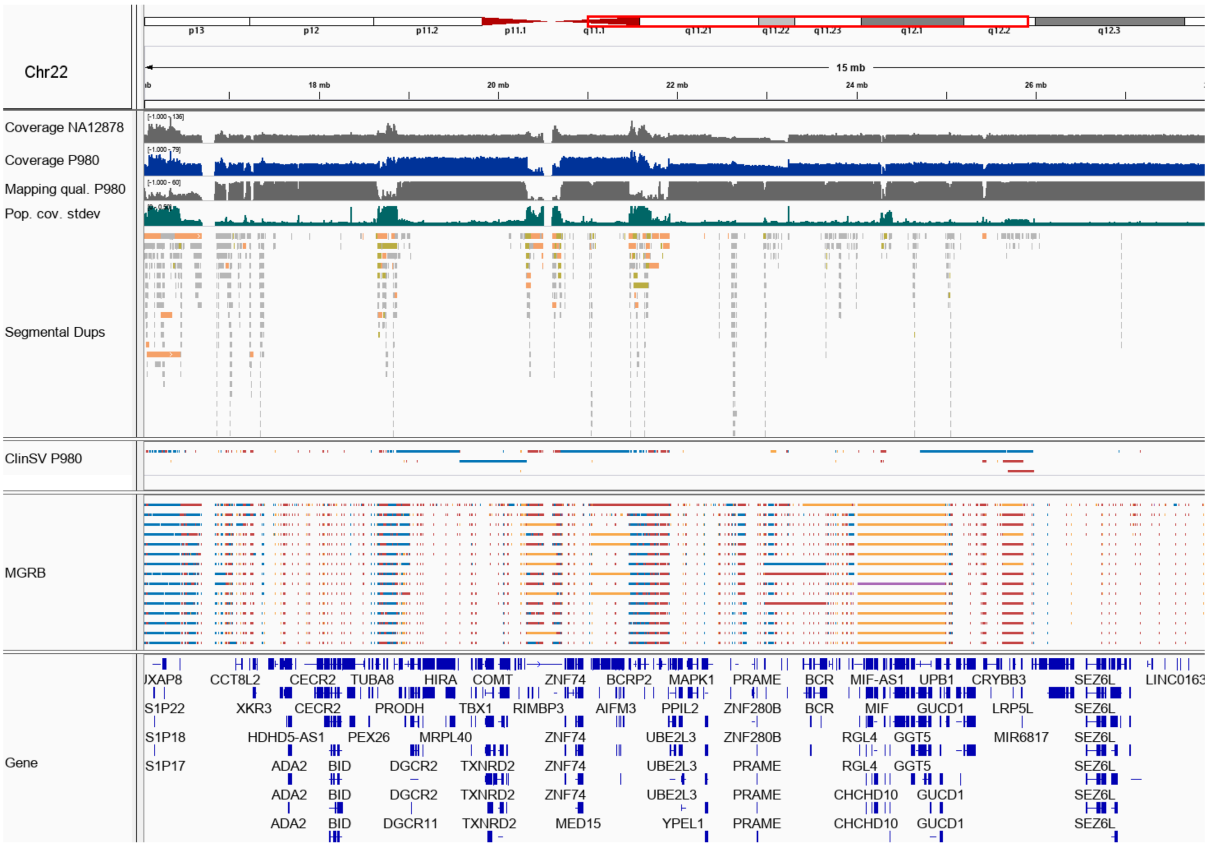
Chr22
p13
p12
p11.2
p11.1
q11.1
q11.21
q11.22
q11.23
q12.1
q12.2
q12.3
15 mb
18 mb
20 mb
22 mb
24 mb
26 mb
Coverage NA12878
Coverage P980
Mapping qual. P980
Pop. cov. stdev
Segmental Dups
ClinSV P980
MGRB
Gene
XAP8
CCT8L2
CECR2
TUBA8
HIRA
COMT
ZNF74
BCRP2
MAPK1
PRAME
BCR
MIF-AS1
UPB1
CRYBB3
SEZ6L
LINC0163
S1P22
XKR3
CECR2
PRODH
TBX1
RIMBP3
AIFM3
PPIL2
ZNF280B
BCR
MIF
GUCD1
LRP5L
SEZ6L
S1P18
HDHD5-AS1
PEX26
MRPL40
ZNF74
UBE2L3
ZNF280B
RGL4
GGT5
MIR6817
SEZ6L
S1P17
ADA2
BID
DGCR2
TXNRD2
ZNF74
UBE2L3
PRAME
RGL4
GGT5
SEZ6L
ADA2
BID
DGCR2
TXNRD2
ZNF74
UBE2L3
PRAME
CHCHD10
GUCD1
SEZ6L
ADA2
BID
DGCR11
TXNRD2
MED15
YPEL1
PRAME
CHCHD10
GUCD1
SEZ6L

b

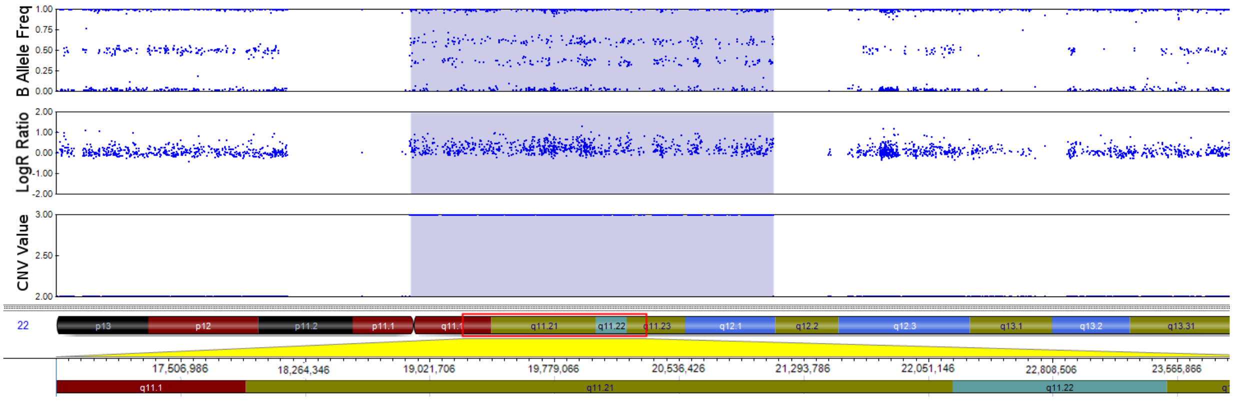
B Allele Freq
1.00
0.75
0.50
0.25
0.00
LogR Ratio
2.00
1.00
0.00
-1.00
-2.00
CNV Value
3.00
2.50
2.00
22
p13
p12
p11.2
p11.1
q11.1
q11.21
q11.22
q11.23
q12.1
q12.2
q12.3
q13.1
q13.2
q13.31
17,506,986
18,264,346
19,021,706
19,779,066
20,536,426
21,293,786
22,051,146
22,808,506
23,565,866
q11.1
q11.21
q11.22

Supplementary Figure 3: Validation of Chromosome 22q11.21 duplication. a) Integrative Genomics Viewer image of the ClinSV call for P980 (duplication Chr22:18873001-21469900). Segmental duplications within this region mediate recurrent deletions and duplications which have been associated with neurodevelopmental disorders, but also result in poorly mapped regions in control samples (MGRB). **b)** Validation by SNP array confirms the presence of a duplication encompassing a minimum region of Chr22:18899402-21109441 (blue highlighted region) which is flanked by low copy repeat regions.

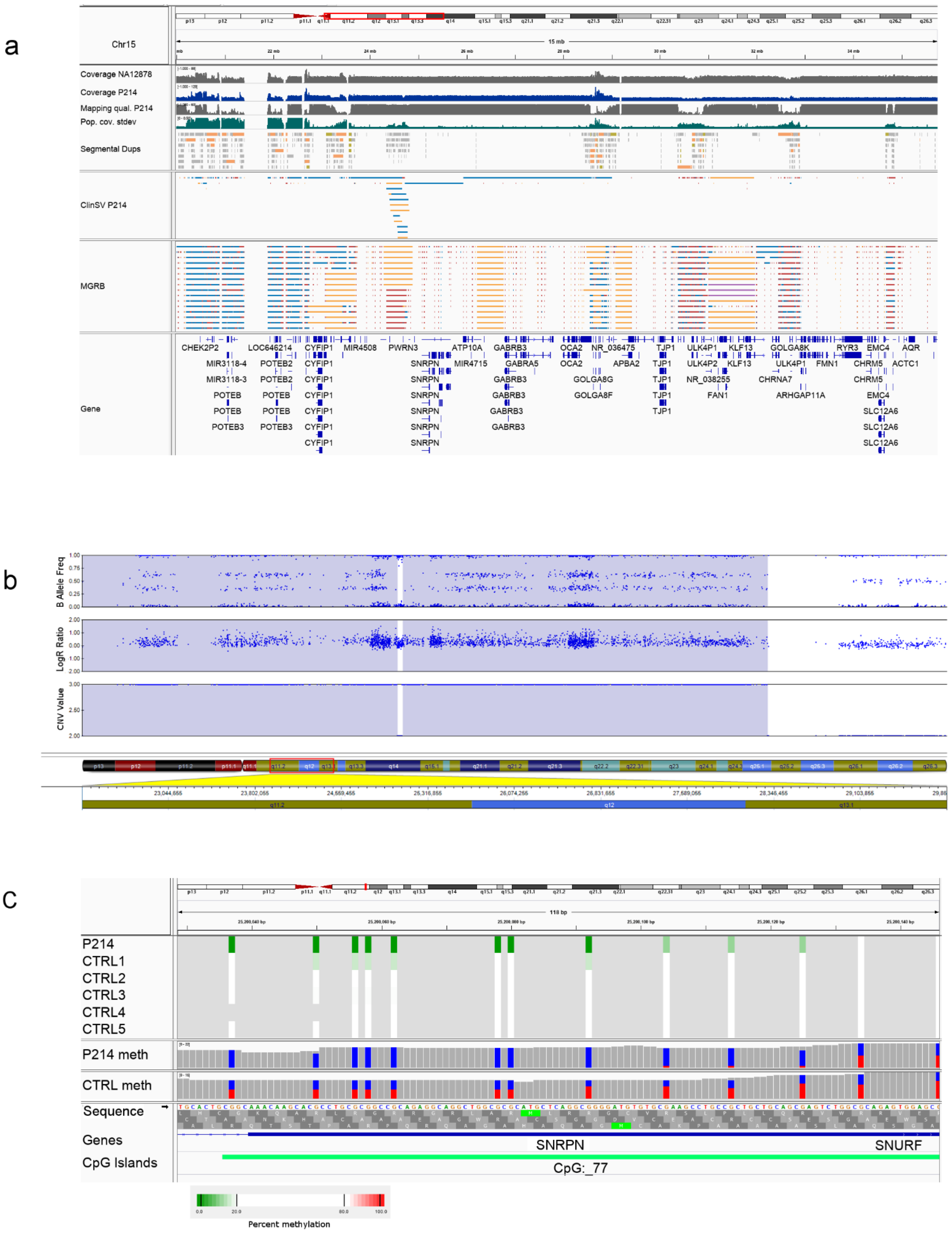
a
Chr15
Coverage NA12878
Coverage P214
Mapping qual. P214
Pop. cov. stdev
Segmental Dups
ClinSV P214
MGRB
Gene
CHEK2P2
LOC646214
CYFIP1
MIR4508
PWRN3
ATP10A
GABRB3
OCA2
NR_036475
TJP1
ULK4P1
KLF13
GOLGA8K
RYR3
EMC4
AQR
MIR3118-4
POTEB2
SNRPN
MIR4715
GABRA5
APBA2
ULK4P2
FMN1
CHRM5
ACTC1
MIR3118-3
GOLGA8G
NR_038255
CHRNA7
POTEB
FAN1
ARHGAP11A
GOLGA8F
SLC12A6
POTEB3
b
B Allele Freq
LogR Ratio
CNV Value
c
P214
CTRL1
CTRL2
CTRL3
CTRL4
CTRL5
P214 meth
CTRL meth
Sequence
Genes
CpG Islands
SNRPN
SNURF
CpG:_77
Percent methylation

Supplementary Figure 4: Validation of Chromosome 15q11.2-q13.1 duplication. a) Integrative Genomics Viewer (IGV) image of the ClinSV calls for P214, likely representing one large duplication event, containing BP1-BP3 of the PWS/AS critical region (Chr15:22722801- 29006700). The proximal region of Chromosome 15 contains multiple low copy repeats which result in increased susceptibility to genomic rearrangement including deletions and duplications. These repetitive regions also result in poorly mapped regions in control samples (MGRB), however large duplications were not observed in any sample in the MGRB database. **b)** Validation by SNP array confirms the presence of a duplication encompassing Chr15:22758737-28299213 (blue highlighted region). Parental samples were unavailable to confirm inheritance pattern for this duplication. **c)** CpG methylation ratio within the promoter of the maternally imprinted *SNRPN* gene. Tracks P214, CTRL1-CTRL5: heatmaps showing the percentage methylation calculated from MethylCapture sequencing data. P214 meth, CTRL meth: sequencing coverage for MethylCapture Sequencing data, with CpG sites indicated by colour. Blue indicates sequencing reads containing a bisulphite converted residue (i.e. not methylated) and red indicates residues protected from bisulphite conversion (i.e methylated residues). Normal copy number of the 15q imprinted region results in methylation ratio of 0.5 for the promoter of *SNRPN*, consistent with expression only from the paternal allele. Increased methylation ratios (>70%) of CpG Island 77 are indicative of maternal origin for 15q duplications, while decreased methylation ratios indicate paternal origin. We observed a decrease in methylation of the 5' end of CpG island 77 in P214, suggesting possible paternal origin for the duplication.

a

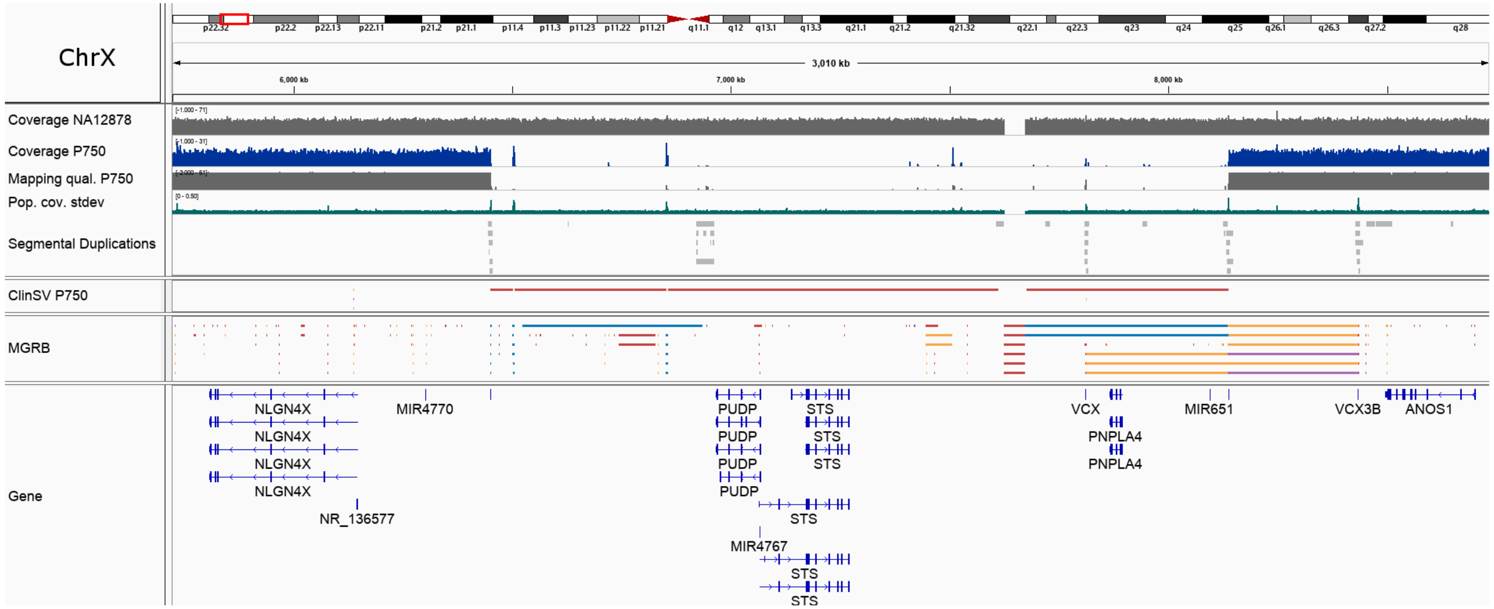

ChrX
3,010 kb
6,000 kb
7,000 kb
8,000 kb
Coverage NA12878
Coverage P750
Mapping qual. P750
Pop. cov. stdev
Segmental Duplications
ClinSV P750
MGRB
Gene
NLGN4X
MIR4770
NR_136577
PUDP
STS
MIR4767
VCX
PNPLA4
MIR651
VCX3B
ANOS1

b

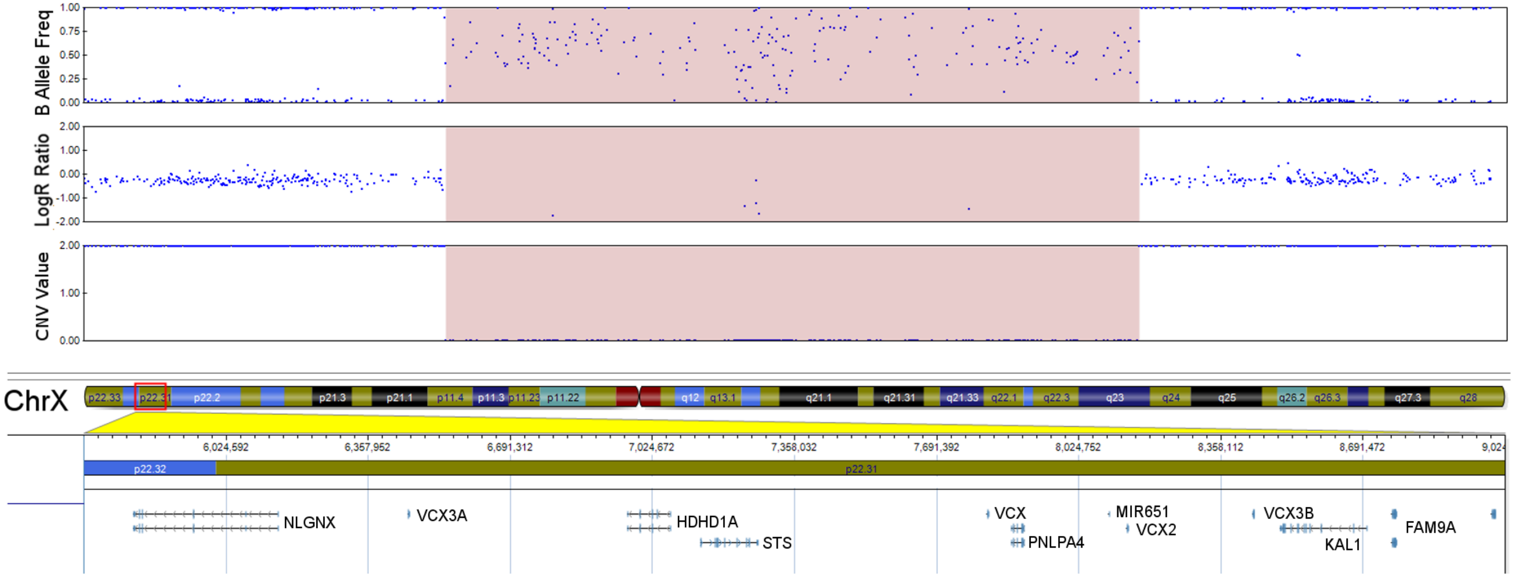

B Allele Freq
LogR Ratio
CNV Value
ChrX
6,024,592
6,357,962
6,691,312
7,024,672
7,358,032
7,691,392
8,024,752
8,358,112
8,691,472
NLGNX
VCX3A
HDHD1A
STS
VCX
PNLPA4
MIR651
VCX2
VCX3B
KAL1
FAM9A

Supplementary Figure 5: Validation of Chromosome Xq22.3 hemizygous deletion. a) Integrative Genomics Viewer image of the ClinSV call for P750 (deletion ChrX:6856601-8138000). Segmental duplications within this region mediate recurrent deletions which cause Xp22.3 microdeletion syndrome. **b)** Validation by SNP array confirmed the presence of a deletion encompassing chrX:6538899-8167012 (red highlighted region).

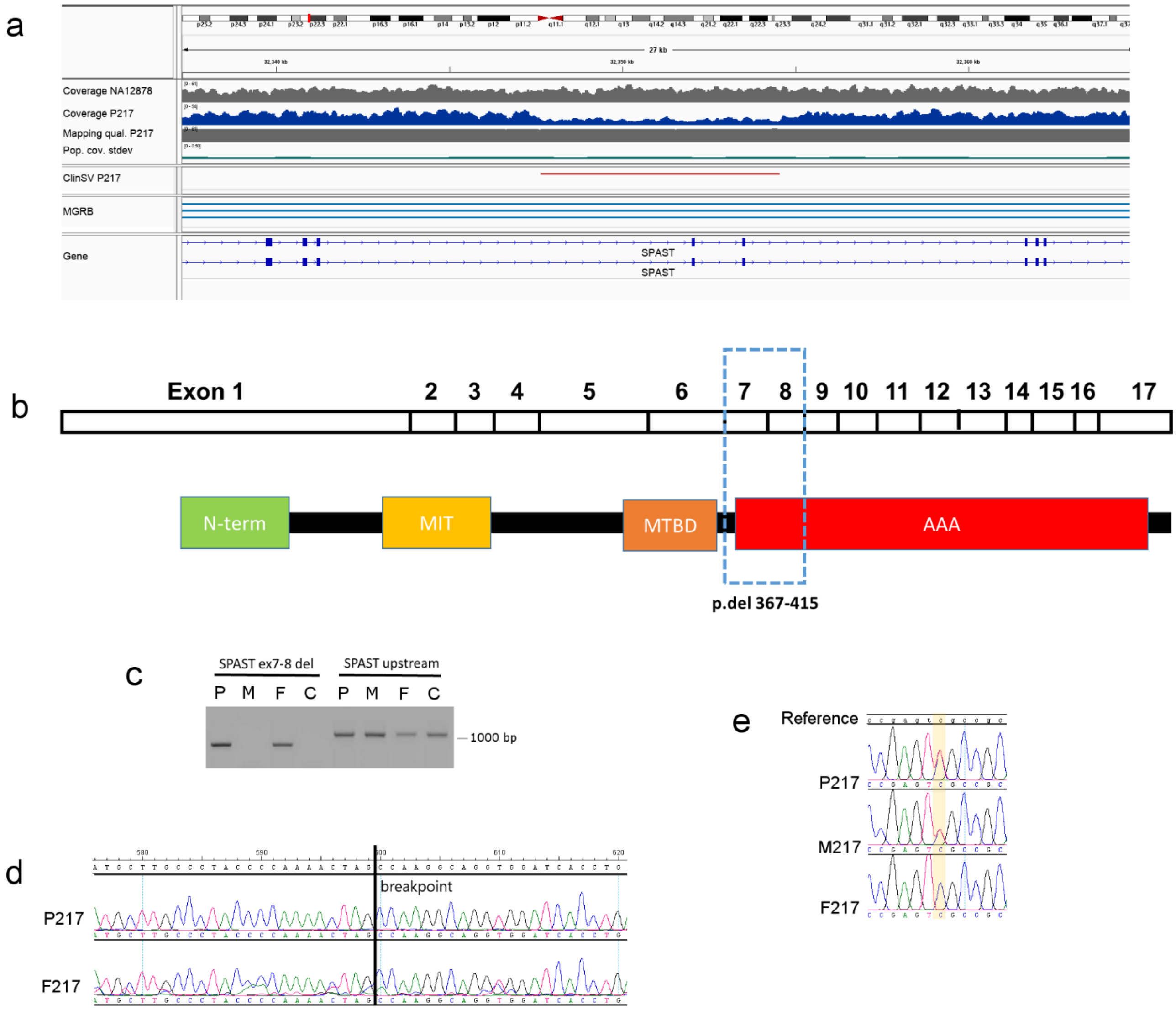

Supplementary Figure 6: Validation of *SPAST* exon 7-8 deletion and *SPAST* p.Ser44Leu modifier allele. a) Integrative Genomics Viewer image of the ClinSV call for P217 (a novel deletion Chr2:32347645-32354557). **b)** Schematic of structure of *SPAST* showing the location of exons 1-17 (top) with respect to protein domains of the protein (bottom). The location of the deletion detected in P217 (resulting in deletion of amino acids 367-415 at the N-terminal end of the AAA domain) is indicated by the blue box. **c)** The *SPAST* exon 7-8 deletion is paternally inherited. PCR with primers flanking the deleted region (SPAST ex7-8 del) gives a 742 bp product for P217 (P) and his father (F), and no product for his mother (M) and an unrelated control sample (C). PCR from the wildtype *SPAST* allele with one primer within the deleted region (SPAST wildtype) gives a product of expected size (1042 bp) for all samples, confirming that both P217 and F217 are heterozygous for the deletion. **d)** Sanger sequencing of the SPAST ex7-8 del PCR product confirms the *SPAST* breakpoints called by ClinSV in both P217 and F217. **e)** Sanger sequencing confirms maternal inheritance of the *SPAST* p.Ser44Leu modifier allele. N-term, N-terminal domain; MIT, Microtubule interacting and trafficking domain; MTBD, Microtubule binding domain; AAA, ATPases Associated with a variety of cellular Activities domain.

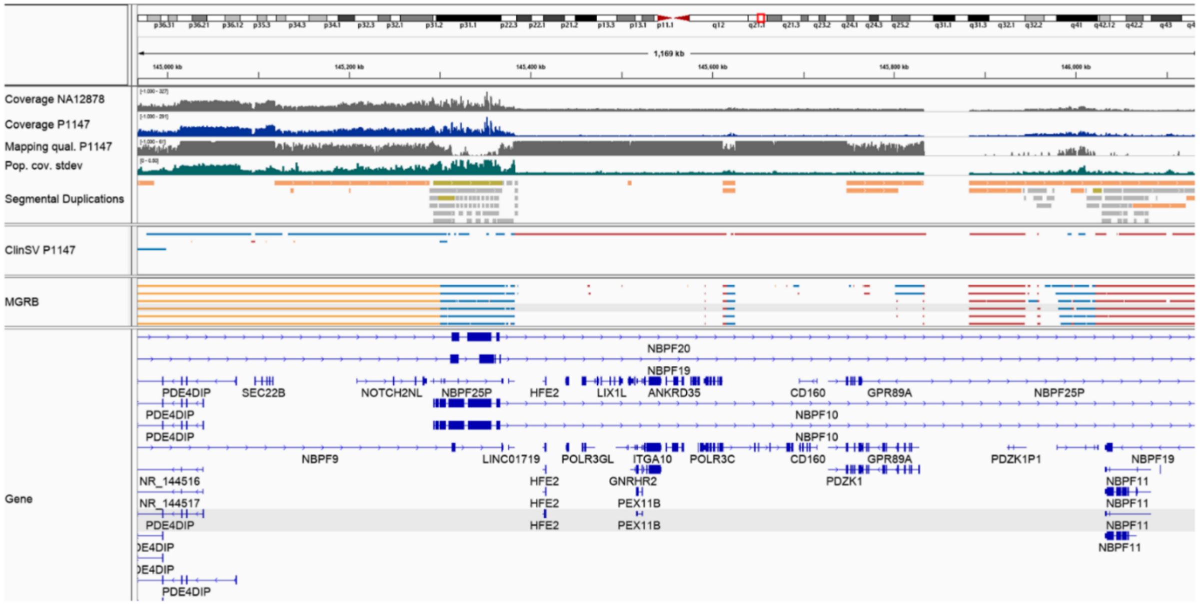
1,169 kb
Coverage NA12878
Coverage P1147
Mapping qual. P1147
Pop. cov. stdev
Segmental Duplications
ClinSV P1147
MGRB
Gene
NBPF20
NBPF19
PDE4DIP
SEC22B
NOTCH2NL
NBPF25P
HFE2
LIX1L
ANKRD35
CD160
GPR89A
NBPF10
NBPF9
LINC01719
POLR3GL
ITGA10
POLR3C
PDZK1P1
NR_144516
NR_144517
GNRHR2
PDZK1
NBPF11
PEX11B

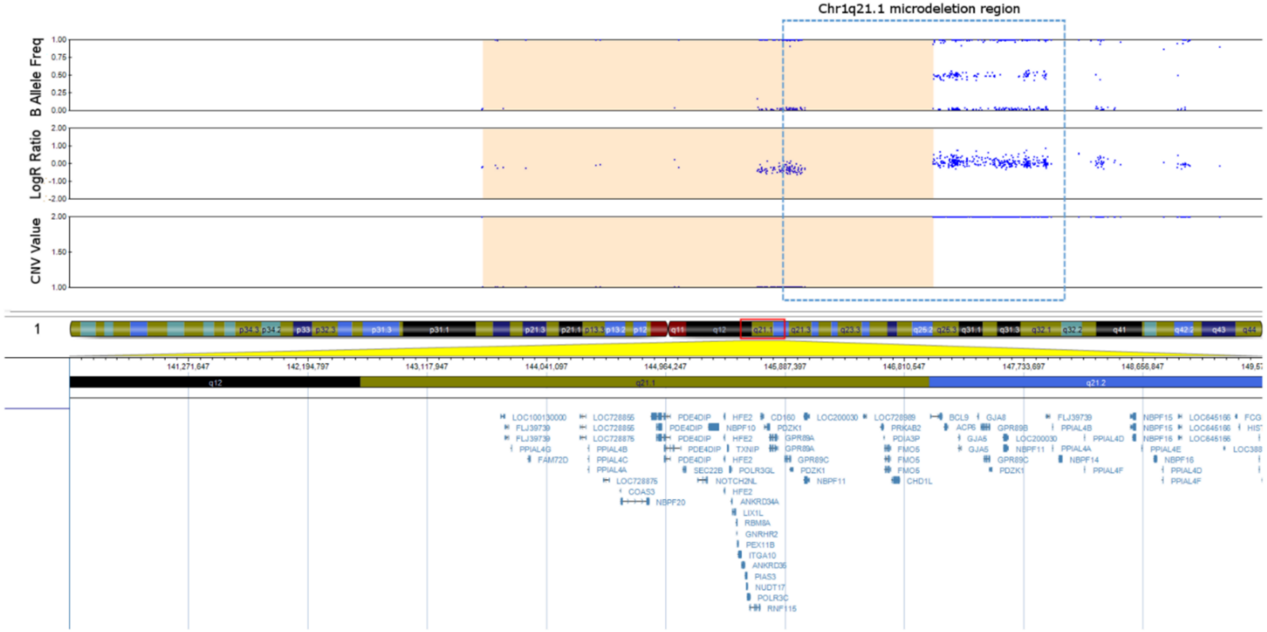
Chr1q21.1 microdeletion region
B Allele Freq
LogR Ratio
CNV Value
1
q12
q21.1
q21.2

Supplementary Figure 7: Validation of Chromosome 1q21.1 deletion. a) Integrative Genomics Viewer image of the ClinSV call for P1147 (Chr1:145382601-145616000 del). Segmental duplications within this region mediate recurrent deletions of a 1.35Mb region which causes Chr1q21.1 deletion syndrome. **b)** Validation by SNP array confirmed the presence of a deletion which was called by CNVPartition as a deletion encompassing Chr1:143545263-147029795 (red highlighted region).

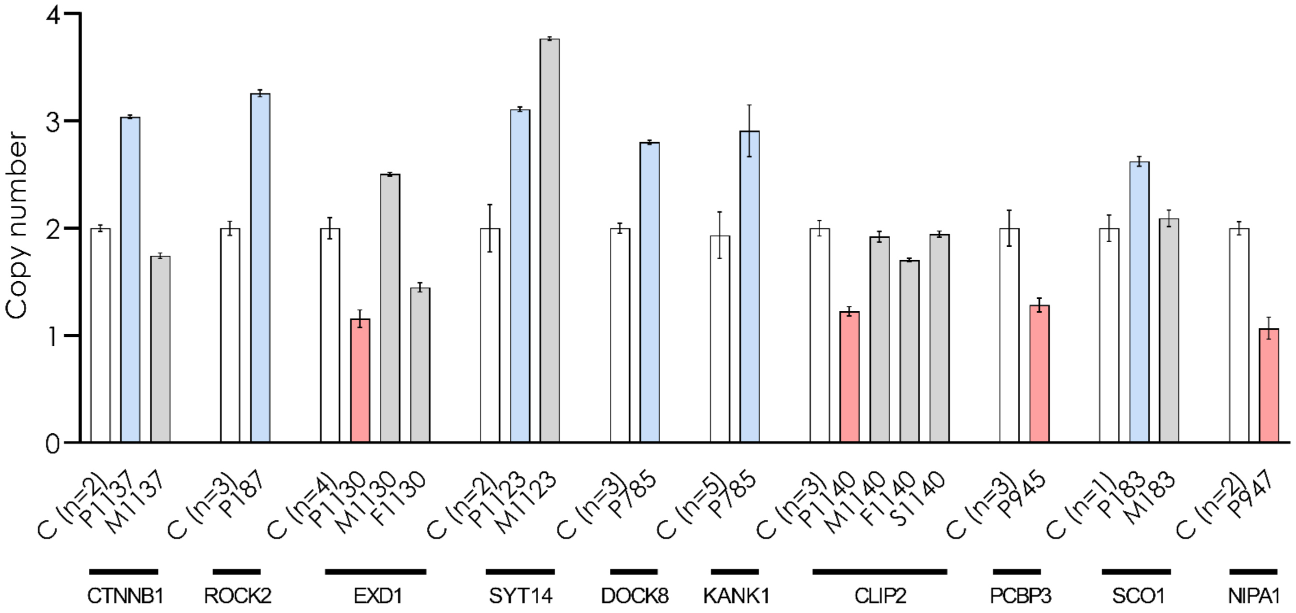

Supplementary Figure 8: Validation of copy number variants of uncertain significance by quantitative PCR (qPCR) or multiplex ligation-dependent amplification (MLPA). See Supplementary methods for details. All data shown is from qPCR, except for *KANK1*. *CTNNB1, ROCK2, EXD1* were independently validated by both qPCR and MLPA. Error bars are SEM.

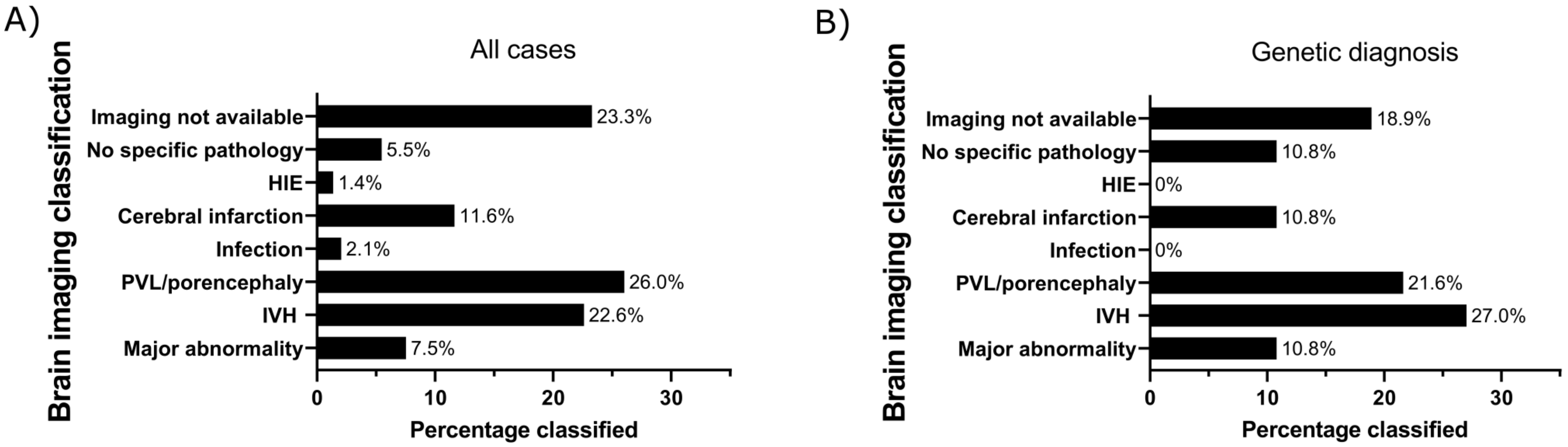

Supplementary Figure 9: Summary of brain imaging findings in all cases in the cohort (A) compared to cases with a genetic diagnosis (B). No significant difference in the distribution of brain imaging classification was found in cases with a genetic diagnosis compared to whole of cohort (Chi-square statistic: 13.78, p=0.088), or between etiological classifications (Table 3) and whole of cohort: Chi square statistic stroke/cardiovascular 2.53, p=0.96; Hereditary spastic paraplegia 14.52, p=0.07; Neurodevelopmental disorder 10.45, p=0.24). HIE, hypoxic ischaemic encephalopathy; PVL, periventricular leukomalacia; IVH, intraventricular haemorrhage.

Supplementary References:

1. Southgate, L. *et al.* Gain-of-function mutations of ARHGAP31, a Cdc42/Rac1 GTPase regulator, cause syndromic cutis aplasia and limb anomalies. *Am J Hum Genet* **88**, 574-585 (2011).
2. Nwosu, B.U., Adhami S Fau - Rogol, A.D. & Rogol, A.D. Stroke in a child with Adams-Oliver syndrome and mixed diabetic ketoacidosis and hyperglycemic hyperosmolar syndrome. **25**(3-4):357-61 (2012).
3. 46. Snape, K.M. *et al.* The spectra of clinical phenotypes in aplasia cutis congenita and terminal transverse limb defects. *Am J Med Genet A* **149A**, 1860-1881 (2009).
4. Isrie, M., Wuyts, W., Van Esch, H. & Devriendt, K. Isolated terminal limb reduction defects: extending the clinical spectrum of Adams-Oliver syndrome and ARHGAP31 mutations. *Am J Med Genet A* **164A**, 1576-1579 (2014).
5. Caron, C. *et al.* CdGAP/ARHGAP31, a Cdc42/Rac1 GTPase regulator, is critical for vascular development and VEGF-mediated angiogenesis. *Scientific Reports* **6**, 27485 (2016).
6. Hassed, S., Li, S., Mulvihill, J., Aston, C. & Palmer, S. Adams-Oliver syndrome review of the literature: Refining the diagnostic phenotype. *Am J Med Genet A* **173**, 790-800 (2017).
7. 74. Parodi, O. *et al.* Spastic paraplegia due to SPAST mutations is modified by the underlying mutation and sex, *Brain*, **141** (12), 3331–3342 (2018).
8. Schickel, J. *et al.* Isoform-specific increase of spastin stability by N-terminal missense variants including intragenic modifiers of SPG4 hereditary spastic paraplegia. *Eur J Neurol* **14**, 1322-1328 (2007).

9. Blumkin, L. *et al.* Expansion of the spectrum of TUBB4A-related disorders: a new phenotype associated with a novel mutation in the TUBB4A gene. *Neurogenetics* **15**, 107-113 (2014).

10. Friedman, J.M. *et al.* Cardiovascular disease in neurofibromatosis 1: report of the NF1 Cardiovascular Task Force. *Genet Med* **4**, 105-111 (2002).

11. Franaszczyk, M. *et al*. Titin Truncating Variants in Dilated Cardiomyopathy - Prevalence and Genotype-Phenotype Correlations. *PloS one* **12**(1) e0169007 (2017).

12. Gudenkauf, F.J., Azamian, M.S., Hunter, J.V., Nayak, A. & Lalani, S.R. A novel CACNA1A variant in a child with early stroke and intractable epilepsy. *Molecular Genetics & Genomic Medicine* **8**, e1383 (2020).

13. Knierim, E. *et al.* Recurrent Stroke Due to a Novel Voltage Sensor Mutation in Cav2.1 Responds to Verapamil. *Stroke* **42**, e14-e17 (2011).

14. Sapio, M.R. *et al.* Naturally occurring carboxypeptidase A6 mutations: effect on enzyme function and association with epilepsy. *J Biol Chem* **287**, 42900-42909 (2012).

15. Petrovski, S. *et al.* Germline De Novo Mutations in GNB1 Cause Severe Neurodevelopmental Disability, Hypotonia, and Seizures. *Am J Hum Genet* **98**, 1001-1010 (2016).

16. Verbeek, E *et al.* COL4A2 mutation associated with familial porencephaly and small-vessel disease.*EJHG* **(20)**8: 844-851 (2012).

17. Sillén, A. *et al.* Spectrum of mutations and sequence variants in the FALDH gene in patients with Sjögren-Larsson syndrome. *Hum Mutat* **12**, 377-384 (1998).

18. Downes, K. *et al.* Diagnostic high-throughput sequencing of 2396 patients with bleeding, thrombotic, and platelet disorders. *Blood* **134**, 2082-2091 (2019).

19. Sanchez-Contreras, M. *et al.* Genetic screening and functional characterization of PDGFRB mutations associated with basal ganglia calcification of unknown etiology. *Hum Mutat* **35**, 964-971 (2014).

20. Kobayashi, Y. *et al.* A point mutation in the mitochondrial tRNALeu(UUR) gene in melas (mitochondrial myopathy, encephalopathy, lactic acidosis and stroke-like episodes). *Biochemical and Biophysical Research Communications* **173**, 816-822 (1990).

21. Segel, R. *et al.* Copy number variations in cryptogenic cerebral palsy. *Neurology* **84**, 1660-1668 (2015).

22. Corbett, M.A. *et al.* Pathogenic copy number variants that affect gene expression contribute to genomic burden in cerebral palsy. *NPJ Genom Med* **3**, 33 (2018).

23. Zarrei, M. *et al.* De novo and rare inherited copy-number variations in the hemiplegic form of cerebral palsy. *Genet Med* **20**, 172-180 (2018).

24. Lionel, A.C. *et al.* Disruption of the ASTN2/TRIM32 locus at 9q33.1 is a risk factor in males for autism spectrum disorders, ADHD and other neurodevelopmental phenotypes. *Human Molecular Genetics* **23**, 2752-2768 (2013).

25. Burnside, R.D. *et al.* Microdeletion/microduplication of proximal 15q11.2 between BP1 and BP2: a susceptibility region for neurological dysfunction including developmental and language delay. *Hum Genet* **130**, 517-528 (2011).

26. Nguyen, A. Prothrombin G20210A polymorphism and thrombophilia. *Mayo Clin Proc* **75**, 595-604 (2000).

27. Zhao, L. *et al.* Prevalence and spectrum of Nkx2.6 mutations in patients with congenital heart disease. *Eur J Med Genet* **57**, 579-586 (2014).

28. Wang, J. *et al.* NKX2-6 mutation predisposes to familial atrial fibrillation. *Int J Mol Med* **34**, 1581-1590 (2014).

29. Mefford, H.C. *et al.* Recurrent rearrangements of chromosome 1q21.1 and variable pediatric phenotypes. *N Engl J Med* **359**, 1685-1699 (2008).

30. Fu, L. et al. Molecular heterogeneity of Krabbe disease. J Inherit Metab Dis 22, 155-162 (1999).

31. Nashabat, M., Al-Khenaizan, S. & Alfadhel, M. Report of a Case that Expands the Phenotype of Infantile Krabbe Disease. *Am J Case Rep* 20, 643-646 (2019).

32. Shao, Y.H. et al. Mutations in GALC cause late-onset Krabbe disease with predominant cerebellar ataxia. *Neurogenetics* 17, 137-141 (2016).

33. Spratley, S.J. et al. Molecular Mechanisms of Disease Pathogenesis Differ in Krabbe Disease Variants. *Traffic* 17, 908-922 (2016).

34. Xu, C., Sakai, N., Taniike, M., Inui, K. & Ozono, K. Six novel mutations detected in the GALC gene in 17 Japanese patients with Krabbe disease, and new genotype-phenotype correlation. *J Hum Genet* 51, 548-554 (2006).

35. Dang, X. et al. Discovery of 6-Phenylhexanamide Derivatives as Potent Stereoselective Mitofusin Activators for the Treatment of Mitochondrial Diseases. *J Med Chem* 63, 7033-7051 (2020).

36. Stunnenberg, B. C. et al. Effect of Mexiletine on Muscle Stiffness in Patients With Nondystrophic Myotonia Evaluated Using Aggregated N-of-1 Trials. *JAMA* 320, 2344-2353 (2018).

37. Novak, K. R., Norman, J., Mitchell, J. R., Pinter, M. J. & Rich, M. M. Sodium channel slow inactivation as a therapeutic target for myotonia congenita. *Annals of neurology* 77, 320-332 (2015).

38. Tonduti, D. et al. TUBB4A-related hypomyelinating leukodystrophy: New insights from a series of 12 patients. *European Journal of Paediatric Neurology* 20, 323-330 (2016).

39. Jones, F. E. et al. 4-Sodium phenyl butyric acid has both efficacy and counter-indicative effects in the treatment of Col4a1 disease. *Human molecular genetics* 28, 628-638 (2019).

40. Lekoubou, A. et al. Effect of long-term oral treatment with L-arginine and idebenone on the prevention of stroke-like episodes in an adult MELAS patient. *Rev Neurol (Paris)* 167(11):852-855 (2011).

41. Sheils, T. K. et al. TCRD and Pharos 2021: mining the human proteome for disease biology. *Nucleic Acids Res* 49(D1):D1334-D1346 (2021).
