# Supplementary material for: Yield of clinically reportable genetic variants in unselected cerebral palsy by whole genome sequencing
Source: NPJ Genom Med. 2021 Sep 16;6:74. doi: 10.1038/s41525-021-00238-0 (PMC8445947; doi:10.1038/s41525-021-00238-0)
Supplement: Supplementary file 2 — Reporting Summary [file 41525_2021_238_MOESM2_ESM.pdf]

nature portfolio

Corresponding author(s): Prof. Jozef Gecz

Last updated by author(s): Jul 22, 2021

Reporting Summary

Nature Portfolio wishes to improve the reproducibility of the work that we publish. This form provides structure for consistency and transparency in reporting. For further information on Nature Portfolio policies, see our Editorial Policies and the Editorial Policy Checklist.

Statistics

For all statistical analyses, confirm that the following items are present in the figure legend, table legend, main text, or Methods section.

n/a	Confirmed	
☐	☒	The exact sample size (*n*) for each experimental group/condition, given as a discrete number and unit of measurement
☐	☒	A statement on whether measurements were taken from distinct samples or whether the same sample was measured repeatedly
☐	☒	The statistical test(s) used AND whether they are one- or two-sided<br>*Only common tests should be described solely by name; describe more complex techniques in the Methods section.*
☐	☒	A description of all covariates tested
☒	☐	A description of any assumptions or corrections, such as tests of normality and adjustment for multiple comparisons
☐	☒	A full description of the statistical parameters including central tendency (e.g. means) or other basic estimates (e.g. regression coefficient) AND variation (e.g. standard deviation) or associated estimates of uncertainty (e.g. confidence intervals)
☐	☒	For null hypothesis testing, the test statistic (e.g. *F*, *t*, *r*) with confidence intervals, effect sizes, degrees of freedom and *P* value noted<br>*Give P values as exact values whenever suitable.*
☒	☐	For Bayesian analysis, information on the choice of priors and Markov chain Monte Carlo settings
☒	☐	For hierarchical and complex designs, identification of the appropriate level for tests and full reporting of outcomes
☐	☒	Estimates of effect sizes (e.g. Cohen's *d*, Pearson's *r*), indicating how they were calculated

Our web collection on statistics for biologists contains articles on many of the points above.

Software and code

Policy information about availability of computer code

Data collection	No custom code was used for data collection.
Data analysis	No custom code was generated.<br>The following commercial or open source software was used in analysis:<br>bcl2fastq v2.16.0<br>BWA-MEM v0.7.10-r789<br>Novosort v1.03.01<br>Samtools v1.1<br>GATK tools v3.3<br>ClinSV 0.9<br>R v4.0.3<br>MinKNOW v20.10.3<br>Guppy v4.2.2<br>minimap2 v2.1<br>Samtools sort v.1.9<br>IGV v2.7.2<br>PennCNV v1.05<br>cnvPartition 3.2.0<br>GenomeStudio 2.0 Software (Illumina)<br>GraphPad Prism 9<br>ANNOVAR (2019Mar23)<br>DNASTAR Lasergene 10, Seqman Pro8<br>TrimGalore v0.4.5

bwa-meth v2.0.1
Picard Tools v2.18.5
MethylDackel v0.2.0
Coffalyser digitalMLPA v.140721.1958

For manuscripts utilizing custom algorithms or software that are central to the research but not yet described in published literature, software must be made available to editors and reviewers. We strongly encourage code deposition in a community repository (e.g. GitHub). See the Nature Portfolio guidelines for submitting code & software for further information.

Data

Policy information about availability of data

All manuscripts must include a data availability statement. This statement should provide the following information, where applicable:
- Accession codes, unique identifiers, or web links for publicly available datasets
- A description of any restrictions on data availability
- For clinical datasets or third party data, please ensure that the statement adheres to our policy

Variants reported in this study have been submitted to ClinVar (SCV001737571-SCV001737616). Subject to compliance with our obligations under human research ethics, whole genome sequencing data are available from the authors on reasonable request.

Field-specific reporting

Please select the one below that is the best fit for your research. If you are not sure, read the appropriate sections before making your selection.

☒ Life sciences ☐ Behavioural & social sciences ☐ Ecological, evolutionary & environmental sciences

For a reference copy of the document with all sections, see nature.com/documents/nr-reporting-summary-flat.pdf

Life sciences study design

All studies must disclose on these points even when the disclosure is negative.

Sample size	150 children with clinically unselected cerebral palsy.
Data exclusions	Variants which were unable to be validated by an orthogonal method are not reported.
Replication	All variants reported were validated by one or more orthogonal method. For copy number variant analysis, 4 technical replicates and >2 controls samples were assayed for qPCR, while for MLPA target probe signal in sample of interest was normalised to control probe signal in that samples as well as target probe signal in 5 other samples.
Randomization	All samples were tested, randomization was not necessary as there was no treatment applied.
Blinding	Blinding was not possible as all samples tested were from affected individuals and there was no treatment applied.

Reporting for specific materials, systems and methods

We require information from authors about some types of materials, experimental systems and methods used in many studies. Here, indicate whether each material, system or method listed is relevant to your study. If you are not sure if a list item applies to your research, read the appropriate section before selecting a response.

Materials & experimental systems

n/a	Involved in the study	
☒	☐	Antibodies
☒	☐	Eukaryotic cell lines
☒	☐	Palaeontology and archaeology
☒	☐	Animals and other organisms
☐	☒	Human research participants
☒	☐	Clinical data
☒	☐	Dual use research of concern

Methods

n/a	Involved in the study	
☒	☐	ChIP-seq
☒	☐	Flow cytometry
☒	☐	MRI-based neuroimaging

Human research participants

Policy information about studies involving human research participants

Population characteristics	All cases in this cohort met internationally accepted inclusion criteria for diagnosis of cerebral palsy, including their condition

Population characteristics	being considered permanent and non-progressive at >4 years of age, as determined by a neurologist or paediatric rehabilitation specialist. Cases were otherwise clinically unselected, with the exception of exclusion of post-neonatal causes of CP. Mean age at initial recruitment was 8.4 years (2.0-18.2 years).
Recruitment	Cases were mostly recruited through Botox clinics in children's hospitals around Australia.
Ethics oversight	Ethical approval for the Biobank and genetic investigations of the Biobank cohort were given by the Adelaide Women's and Children's Health Network (WCHN) Human Research Ethics Committee (records HREC/12/WCHN/61 and HREC/15/WCHN/148).

Note that full information on the approval of the study protocol must also be provided in the manuscript.
